# Guiding Evidence-Based Classification in Para Sporting Populations: A Systematic Review of Impairment Measures and Activity Limitations

**DOI:** 10.1007/s40279-024-02132-y

**Published:** 2024-11-22

**Authors:** Taylor M. Wileman, Marnee J. McKay, Daniel A. Hackett, Timothy J. Watson, Jennifer Fleeton, Ché Fornusek

**Affiliations:** 1 School of Health Sciences, Faculty of Medicine and Health, The University of Sydney, Sydney, NSW Australia; 2 School of Behavioural and Health Sciences, Faculty of Health Sciences, Australian Catholic University, Sydney, NSW Australia

## Abstract

**Background:**

As the focus of classification shifts towards an evidence-based approach, it is crucial to establish a robust system that relies on valid and reliable measures of impairment to ensure legitimate and competitive opportunities for all Para athletes. However, the lack of methods that possess the necessary measurement properties for assessing impairments in Para sporting populations presents significant challenges to developing an evidence-based classification system.

**Objective:**

This review aimed to identify and evaluate measures of impairment and activity limitation measures that have been used to assess eligible impairments in Para sport athletes for potential use in evidence-based classification.

**Methods:**

Six electronic databases (MEDLINE, Embase, SPORTDiscus, CINAHL, Scopus, Web of Science) were searched from their earliest record to December 2023.

**Results:**

Fifty-one articles were identified, with twenty-one studies focusing on physical impairment measures. Isometric and grip strength emerged as effective measures. Coordination measures, such as tapping tasks, showed variations with performance. Additionally, six studies focused on intellectual impairments, revealing differences between impaired and non-impaired athletes through generic cognitive tests. Vision impairment measures, including visual acuity and visual field assessments, displayed varying associations with performance across sports.

**Conclusions:**

Although research on evidence-based classification in Para sport is limited, this review provides valuable insights for sports in developing a testing battery that adheres to evidence-based protocols. Ongoing research efforts by sport governing bodies to prioritise research in this area will improve our understanding of the impairment–performance relationship, leading to better decision making and increased credibility in Para sport classification systems.

**Supplementary Information:**

The online version contains supplementary material available at 10.1007/s40279-024-02132-y.

## Key Points

**Table:** 

There is a need to examine the test–retest reliability of physical impairment measures developed for use in evidence-based classification in Para sport athletes.
Cognitive-motor dual tasking measures may be more effective distinguishing between groups of athletes with and without an intellectual impairment when compared to tests in isolation.
Future research is needed to consider the influence of factors such as training, fatigue and how susceptible each measure is to intentional misrepresentation.

## Introduction

Classification systems play a crucial role in legitimising success in Paralympic sport as they determine participation eligibility and minimise the impact of eligible impairments on the result of competition [1, 2]. Each Paralympic sport has its own class profiles that categorise athletes according to how the extent and nature of their impairment impacts their ability to perform sport-specific skills [3]. Further to classification systems being sport-specific, the International Paralympic Committee (IPC) adopted the Athlete Classification Code, which mandates the development of evidence-based classification methods in Para sports (sport directly governed by the IPC or one of its member organisations) [2, 3]. This was implemented to ensure athletes are allocated to a class based on empirical evidence rather than a subjective evaluation through expert opinion [4].

Although the transition to or implementation of an evidence-based classification system can be complicated by the unique intellectual and physical demands of each sport, the IPC’s position stand offers guidelines for conducting evidence-based classification research [5]. It is essential that sports and researchers develop evidence-based classification systems by specifying eligible impairment types for the sport, developing valid measures of impairment, developing sport-specific measures of performance, and assessing the strength of association between performance and impairment measures [2]. Further research in these areas will not only increase the transparency and strength of classification for existing sports but also new sports seeking to join the Paralympic Movement.

There are ten impairment types that can compete in Para sports (athetosis, hypertonia, ataxia, short stature, leg length difference, impaired muscle power, impaired range of movement, limb deficiency, and visual and intellectual impairments) [5]. It is at the discretion of each sport to determine what impairment types are eligible to compete [5]. However, they must ensure that appropriate methods for assessing eligible impairments are implemented. In accordance with the IPC Position Stand, several measurement properties are required for impairment measures to be considered valid [5]. These essential properties include being reliable, ratio scaled, parsimonious, sport specific, impairment specific and resistant to the effects of training [2, 6]. The use of measurement methods that comply with this criterion will provide the evidence to encourage decision making, providing athletes, coaches, stakeholders and spectators with confidence in the classification system developed for the sport.

Currently, assessments of physical impairments in Para sport typically involve clinical measures such as manual muscle testing to evaluate strength and goniometry to measure range of motion (ROM). Additionally, observed movements of ‘coordination’ or use of pre-established scales (i.e. Modified Ashworth Scale) are used to assess hypertonia, ataxia and athetosis [7–16]. While expert classifiers perform these assessments, measures of strength via manual muscle testing and coordination assessed through observation or scales do not comply with the IPC’s requirements for evidence-based classification. Specifically, the ordinal data collected from these assessments hinder the ability to explore the relationship between impairment and performance [17–20]. Furthermore, these methods are non-instrumented, which contributes to their poor reliability [21]. For athletes with intellectual impairments, eligibility determination includes Intelligence Quotient (IQ) measures. However, the resulting score is a broad composite measure, with only a limited subset of cognitive abilities assessed relevant for understanding the relationship between impairment and sports performance [22]. Similarly, for athletes with vision impairments, relying solely on visual acuity and visual field assessments alone might not be enough [6], emphasising the need for additional measures to understand how vision impacts sports performance. Addressing these challenges requires ongoing research to develop evidence-based approaches that are valid and reliable.

In addition to the establishment of valid impairment measures, standardised sport-specific tests (referred to as measures of activity limitation) need to be developed to quantify the relationship between impairment and performance [2, 5]. The difficulty associated with determining sport performance varies considerably between sports. For example, the measurement of performance in sports such as powerlifting is relatively straightforward as it relies on a single quantifiable outcome (i.e. better performance is achieved by lifting a heavier weight) [6]. However, swimming performance is more complex to assess because performance determination is dependent on multiple activities (i.e. dive, swim stroke, tumble turn). However, given that overall performance in swimming can be quantified by time, the relative importance of each activity on performance can be measured [6]. Evaluating performance in team sports such as cerebral palsy (CP) football is even more complex as unlike swimming and powerlifting there is no single quantifiable measure of performance. While identifying the performance determinants in CP football (i.e. dribbling, passing, speed) is relatively simple, determining the relative importance of these activities for individual player performance is challenging.

As the focus of classification shifts to an evidence-based approach, a greater insight into the relationship between impairment types and impairment-specific activity limitations across sports is needed. To date, one review regarding evidence-based classification has been published. This study summarised methods for assessing upper body strength, coordination and ROM impairments that could be used in the development of an evidence-based system for wheelchair sports [24]. However, its relevance to other Para sports is limited as it did not consider measures related to lower limb impairment, vision and intellectual impairment, or measures of activity limitation. Therefore, the purpose of this review was to systematically identify measures of impairment and activity limitation measures that have been used to assess eligible impairments in Para sport athletes for potential use in evidence-based classification. Furthermore, this review assesses the validity and/or reliability of the included physical and intellectual impairment measures, while also examining associations between impairment measures and sports performance across physical, intellectual and vision impairment groups.

## Methods

This systematic review was prepared in accordance with the Preferred Reporting Items for Systematic Reviews and Meta Analyses (PRISMA) statement [25].

### Search Strategy

Electronic database searches were performed in MEDLINE, Embase, SPORTDiscus, CINAHL, Scopus and Web of Science from their earliest record to December 2023. The search strategy was developed using three umbrella terms: type of assessments (e.g. strength, coordination, activity limitation), impairment types (e.g. intellectual, visual, physical) and sport (e.g. archery, athletics, boccia, swimming, football). Please refer to the Electronic Supplementary File 1 for the full search strategy. All records were downloaded to EndNote X9 (Clarivate Analytics, Philadelphia, PA) and then uploaded to Covidence (Veritas Health Innovation, Melbourne, Australia) where duplicates were removed, and the screening process completed.

### Study Inclusion and Exclusion

Studies were included if they met the following criteria: (1) literature published as a full-text article in English, (2) assessed eligible impairments in Para sport athletes for the purpose of classification research, using methods to evaluate impairment or activity limitation applicable for use in an evidence-based classification and (3) included participants aged ≥ 12 years (from all the International Federations recognised by the IPC that stipulated eligibility around age, 12 years was the minimum age found eligible to compete in IPC competitions or World Para sanctioned competitions).

Studies were excluded if they used a functional classification system (i.e. no direct measure of impairment) or assessed injury, concussion, return to sport, pain, fatigue, rehabilitation, surgery, electrical stimulation, orthosis, fitness measures, physiological, metabolic and training responses. Review papers, unpublished studies, conference proceedings and studies that focused solely on non-disabled populations when developing impairment or activity limitation measures were also excluded.

### Eligibility Screening

Titles and abstracts of all the retrieved articles were independently assessed by three reviewers (TMW, CF, JF), in which two of whom screened half each (CF, JF). Full-text articles were retrieved for the remaining articles and independently reviewed by the same three authors with reasons for exclusion recorded. During each stage, differences were assessed, and a consensus reached by discussion with the corresponding author (MM).

### Data Extraction

For each of the included studies, the following data were extracted: author/s and publication year, study objective, sample size, impairment types, testing batteries, methods of assessment, outcome measures and results. One reviewer (TMW) extracted and recorded all data on a customised spreadsheet, which was validated by a second reviewer (TJW).

### Quality Assessment

The methodological quality of included studies was independently assessed by the same two reviewers (TMW, TJW) using the Joanna Briggs Institute Critical Appraisal Checklist for Analytical Cross-Sectional Studies [26]. Any discrepancies were resolved through discussion. Studies were characterised as: (1) low risk of bias if ‘yes’ scores were at least 70%, (2) moderate risk of bias if ‘yes’ scores were between 50 and 69% and (3) high risk of bias if ‘yes’ scores were below 50% [27–29].

## Results

### Identification and Selection of Studies

**Fig. 1:**
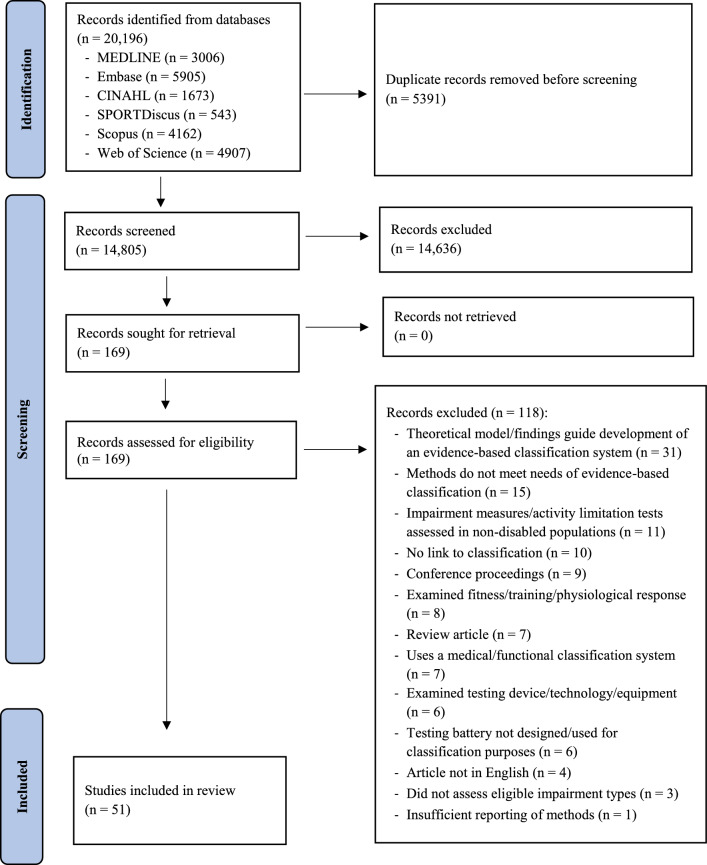
Preferred Reporting Items for Systematic Reviews and Meta Analyses (PRISMA) flowchart for identification and inclusion of relevant studies

The initial search strategy produced 20,196 records. After duplicates were removed, 14,805 studies remained of which 14,636 were excluded based on the inclusion and exclusion criteria during the initial screening of titles and abstracts. One hundred and sixty-nine articles were included for a full-text review with 118 excluded, leaving a total of 51 articles for review (Fig. 1).

### Summary of Included Studies

#### Impairment Measures

Of the fifty-one studies included, thirty-five focused on impairment measures. The physical impairment measures were further categorised into strength, coordination and ROM for ease of analysis. Thirteen studies examined methods for measuring strength impairment [18, 30–41], six investigated coordination impairment [19, 42–46] and five addressed ROM impairment [35, 37, 43, 47, 48]. Studies investigating strength impairment examined a total of 467 participants (373 disabled athletes and 94 non-disabled participants). Coordination impairment was assessed in a total of 543 participants (427 disabled athletes and 116 non-disabled participants). For studies examining ROM impairment, a total of 206 participants were assessed (126 disabled athletes and 80 non-disabled participants). Additionally, six studies examined intellectual impairment measures [49–54], involving a total of 1247 participants (820 disabled athletes and 427 non-disabled participants). Vision impairment measures were assessed in eight studies [55–62], with a total of 534 Para athletes included. Extracted data are shown for studies assessing strength impairments (Table 1), coordination impairments (Table 2), ROM impairments (Table 3), intellectual impairments (Table 4) and vision impairments (Table 5).

#### Activity Limitation Measures

Nineteen studies explored activity limitation measures [23, 31, 45, 56, 63–77], with a total of 1776 participants assessed (1534 disabled athletes and 242 non-disabled participants). Data extracted from studies focusing on activity limitation measures are presented in Table 6. Please refer to the Electronic Supplementary File 2 for detailed descriptions of all assessment methods (impairment and activity limitations measures).

### Strength Impairment Measures

Table 1 outlines the methods identified for measuring strength impairments, which include isometric strength, grip strength and trunk stability. Most studies focused on wheelchair sports with three studies focusing on ambulant Para sport athletes. Regarding the analysis methods employed by each of the studies, seven examined the validity of the method to measure strength impairment [18, 30–33, 38, 40], three investigated the reliability of strength impairment measures [18, 34, 38] and ten explored the relationship between strength impairment and performance [18, 31–37, 39, 41].

**Table 1:** Strength impairment measures

Study	Population	Sample size	Assessments Equipment (E), outcome measure (OM)	Key findings
Altmann et al. [30]	WC basketball and WC rugby players with ≥ 1 year experience	*N* = 34 (sex NR, 18–59 y)	(i) Static sitting balance *E:* Chair mounted on AMTI forceplate *OM:* Sway area in mm^2^ (ii) Dynamic sitting balance *E:* Chair mounted on AMTI forceplate *OM:* Excursion of COP displacement in mm (iii) Isometric trunk strength *E:* Load cell *OM:* Force in Newtons	*Static sitting balance* No significant effect of TIC on stable (*p* = 0.528) or unstable surface (*p* = 0.236) *Dynamic sitting balance* Main effect of TIC on left oblique forward–right oblique backward direction (*p* = 0.12) Larger excursion in TIC 1.5 compared with TIC 0.5 and 1.0 *Isometric Trunk Strength* Significant effect of TIC in all directions (*p* < 0.001) Left lateral strength greater in TIC 1.0 and 1.5 than TIC 0 Right and forward strength greater in TIC 0.5, 1.0 and 1.5 compared with TIC 0
Altmann et al. [31]	WC basketball and WC rugby players with ≥ 1 year experience	*N* = 27 (M, 37.4 ± 10.2 y)	**Impairment** (i) Isometric trunk strength *E:* Load cell *OM:* Force in Newtons **Activity limitation** (i) WC tilt *E:* Tape measure attached to pulley system *OM:* Difference between max and initial height (mm) (ii) Acceleration *E*: Cheetah LMT attached to WC *OM*: Time to push 1 m (s) (iii) Sprint momentum *OM:* Average velocity of first 2-m multiplied by body mass (kg m s^−1^)	*Lateral isometric force and WC tilt* Significant moderate correlation between left and right mean isometric force and left and right mean tilt height (*r* = 0.50, *p* = 0.007) Significant difference in tilt height between cluster 1, 2 and 3 *Forward isometric force and acceleration test* Significant moderate correlation (*r* = 0.59, *p* = 0.001) Significant difference between clusters 1 and 4 *Forward isometric force and sprint momentum* Strong significant correlation (*r* = 0.79, *p* = 0.0001) Significant difference between clusters 1 and 4, 1 and 3, 2 and 3
Beckman et al. [32]	Physically active runners who were competitive or regularly competed in a sport where running speed was a determinant of performance	*N* = 41 (M) RBI: n = 13 24.3 ± 9.4 y NDR: n = 28 23.1 ± 4.1 y	**Impairment** (i) Leg flexor strength *E:* Load cell *OM:* Force in Newtons (ii) Leg extensor strength *E:* Load cell *OM:* Force in Newtons (iii) Plantarflexor strength *E:* Load cell *OM:* Force in Newtons **Performance** 60-m maximal sprint (i) Acceleration phase: 0–15 m (ii) Top speed phase: 30–60 m *E:* Cheetah iRex *OM:* Time (s)	Strength scores were significantly lower for RBI when compared with NDR on more affected side for plantarflexion (1072 vs 1508N), leg flexion (176 vs 243N) and leg extension (993 vs 1661N) RBI were significantly slower than NDR in the acceleration (3.18 s ± 0.33 vs 2.76 s ± 0.19) and top speed phase (4.31 s ± 0.64 vs 3.76 s ± 0.27)
Connick et al. [33]	International WC track athletes with an official classification (T51–T54)	*N* = 32 (M, 32.2 ± 9.0 y)	**Impairment** (i) Left and right arm extension *E:* Load cell *OM:* Force in Newtons (ii) Arm extension and trunk flexion *E:* Load cell *OM:* Force in Newtons (iii) Isolated trunk flexion *E:* Load cell *OM:* Force in Newtons (iv) Grip strength *E:* Wall-mounted isometric wrist dynamometer *OM:* Force in Newtons **Performance** (i) Maximal 15-m sprint from standstill (ii) Top speed reached from 150 m to finish line (absolute) *E:* Laser device *OM:* Top speed measured in m s^−1^	Significant moderate-to-strong correlations found between all impairment measures and 0–15 m speed (*r* = 0.54–0.83) and absolute top speed (*r* = 0.61–0.88) Isolated trunk flexion had the weakest correlation, dominant arm extension had the strongest correlation *Cluster analysis* 4 clusters deemed optimal Significant difference between clusters 3 and 4 for all impairment measures Significant difference in both 0–15 m speed and absolute top speed for clusters 1 and 2, 2 and 3 Significant difference between clusters 3 and 4 for absolute top speed
Domínquez-Díez et al [34]	(i) WC basketball, WC slalom and paratriathlon athletes (ii) Non-disabled WC athlete coaches and adapted physical education teachers (CG)	*N* = 22 (Sex NR) WC athletes: *n* = 16 ANI: *n* = 5 (32 ± 10 y) IMP: *n* = 11 (36 ± 11 y) CG: *n* = 6 (30 ± 4 y)	**Impairment** Isometric propulsion strength test *E:* Load cell *OM:* Force in Newtons **Performance** (i) WC change of direction ability *E:* Timing gates *OM:* Time (s) (ii) Linear WC sprint test *E:* Timing gates *OM:* Time (s)	*Intra-session reliability* Peak force values displayed excellent relative intra-session reliability for all groups (0.90 < ICC < 0.99) for the pushing action of the isometric propulsion strength test *Isometric propulsion and WC performance* ANI group had the lowest scores for peak pushing force (*p* < 0.05; ES = − 1.47/ − 1.67) and linear WC sprint test (*p* < 0.01; ES = 1.40/1.91) compared with IMP and CG ANI exhibited poorer performance in WC change of direction compared with IMP group (*p* < 0.01; ES = 1.79*)* No significant differences found between measures for IMP and CG No correlation found between impairment and performance measures for ANI and IMP groups
Hogarth et al. [18]	(i) Para swimmers who had a National or International classification, undertaking planned training regimes and competing at a national or international level (ii) Non-disabled participants who were recreationally active	*N* = 72 (41 M, 31 F) Para swimmers: *n* = 42 Hypertonia: *n* = 23 (17 M, 26.5 ± 7.0 y; 6 F, 19.8 ± 4.1 y) IMP: *n* = 19 (9 M, 31.5 ± 7.7 y; 10 F, 29.2 ± 10.2 y) Non-disabled: *n* = 30 (15 M, 24 ± 4 y; 15 F, 23 ± 5 y)	**Impairment** (i) Shoulder flexion *E:* Load cell *OM:* Force in Newtons (ii) Shoulder extension *E:* Load cell *OM:* Force in Newtons (iii) Hip flexion *E:* Load cell *OM:* Force in Newtons (iv) Hip extension *E:* Load cell *OM:* Force in Newtons **Performance** 10-m maximal freestyle swim *E:* Video camera *OM:* Speed measured in m.s^−1^	*Para swimmers with hypertonia* Dominant shoulder flexion (*r* = 0.66, *p* = < 0.01) and dominant hip flexion (*r* = 0.44, *p* = 0.05) significantly correlated with swim speed *Para swimmers with IMP* No significant correlations between strength scores and swim speed *Entire cohort of Para swimmers* Dominant shoulder flexion only strength score that did not significantly correlate with swim speed (*r* = 0.15, *p* = 0.35) 96% male and 94% female Para swimmers were successfully classified using random forest algorithms *Reliability (non-disabled only)* ICC = 0.85–0.97; CV = 6.4–9.1%; SEM 8.6–17.4N for all strength measures
Hyde et al. [35]	WC rugby, WC basketball and seated throw athletes (athletics) who had an eligible impairment, were currently training and competing in their respective Paralympic sport	*N* = 10 (8 M, 2 F; 32 ± 10 y)	(i) Throwing arm push test *E:* Load cell *OM:* Force in Newtons (ii) Push/pull synergy test *E:* Load cell *OM:* Force in Newtons (iii) Trunk flexion test *E:* Load cell *OM:* Force in Newtons (iv) Grip strength *E:* Grip strength dynamometer *OM:* Measured in kg	No significant correlations were found between hand release speed during throwing with and without an assistive device for throwing arm push (*r* = 0.01–0.31), trunk flexion (*r* = 0.50–0.58) Significant correlations were found between hand release speed during throwing with and without an assistive device for grip strength (*r* = 0.59–0.77), push/pull synergy (*r* = 0.81–0.84)
Liljedahl et al. [36]	Para cyclists with leg impairments	*N* = 56 (44 M, 12 F; 31.7 ± 9.4 y)	**Impairment** (i) Isometric leg push/pull *E:* Force transducer *OM:* Peak force measured in N/kg (ii) Dynamic leg push/pull *E:* Bicycle attached to cycle ergometer *OM:* Peak power expressed as W/kg **Performance** (i) 20-s sprint cycle test *E:* Bicycle attached to cycle ergometer *OM:* Mean power output expressed as W/kg (ii) Official time trial results retrieved from Internet *OM:* Speed km/h	MMT measures of hip, knee, ankle extension and flexion are associated with isometric push (*R*^2^ = 0.49, *p* = 0.004), dynamic push (*R*^2^ = 0.35, *p* = 0.002) and dynamic pull (*R*^2^ = 0.28, *p* = 0.041) but not isometric pull (*R*^2^ = 0.21, *p* = 0.075) Significant correlations were found between isometric and dynamic push (*r* = 0.63, *p* < 0.001), isometric and dynamic pull (*r* = 0.54, *p* < 0.001) Isometric and dynamic tests were significantly associated with mean 20 s sprint power: isometric push (*R*^2^ = 0.35, *p* < 0.001), isometric pull (*R*^2^ = 0.16, *p* = 0.003), dynamic push (*R*^2^ = 0.41, *p* < 0.001), dynamic pull (*R*^2^ = 0.32, *p* < 0.001) Isometric and dynamic tests were significantly associated with race speed: isometric push (*R*^2^ = 0.41, *p* = 0.007), isometric pull (*R*^2^ = 0.38, *p* = 0.040), dynamic push (*R*^2^ = 0.50, *p* < 0.001), dynamic pull (*R*^2^ = 0.43, *p* = 0.004)
Liu et al [37]	Para alpine skiing athletes who had a minimum of 6 months experience and an official classification	*N* = 38 (27 M, 11 F; 23.5 ± 5.4 y)	**Impairment** Isometric trunk strength test *E:* Cable connected to wall mounted dynamometer *OM:* Force in Newtons **Performance** Board tilt test (simulated skiing test) *E:* Horizontal test board and two rolling curved boards *OM:* Maximal board tilt angle (◦)	Significant correlation found between forward isometric truck force and board tilt test (*r* = 0.692, *p* < 0.001) Significant correlation found between lateral isometric trunk force and board tilt test (*r* = 0.775, *p* < 0.001)
Mason et al. [38]	(i) WC rugby athletes with impaired arm strength (ii) Able-bodied participants with previous resistance training experience	*N* = 50 (35 M, 15 F) WC rugby: *n* = 20 (M, 31 ± 5 y) Able bodied:* n* = 30 (15 M, 27 ± 4 y; 15 F, 25 ± 5 y)	(i) Shoulder flexion/extension *E:* Load cell *OM:* Force in Newtons (ii) Elbow flexion/extension *E:* Load cell *OM:* Force in Newtons	WC athletes produced significantly less force compared with able-bodied participants for all strength measures In all measures, female able-bodied participants produced significantly less force than male able-bodied participants *Test–retest reliability (Able-bodied participants)* Acceptable levels of reliability (ICCs ≥ 0.97, SEM ≤ 19.3 N and CV ≤ 8.4%)
Mason et al. [39]	WC rugby athletes with no trunk function	*N* = 57 (53 M, 4 F; 33 ± 7 y)	**Impairment** (i) Shoulder flexion/extension *E:* Load cell *OM:* Force in Newtons (ii) Elbow flexion/extension *E:* Load cell *OM:* Force in Newtons iii) Push and pull test *E:* Load cell *OM:* Force in Newtons **Performance** 10-m maximal sprint *E:* Timing gates *OM*: Time	All strength measures significantly correlated with 2-m and 10-m sprint times (*r* = ≥ − 0.43; *p* ≤ 0.0005) *Cluster analysis (female data excluded)* 3 cluster structure: 19% athletes assigned to different cluster 4 cluster structure: 40% athletes assigned to different cluster 3 cluster structure found to be a valid structure when compared to 4 cluster and existing IWRF system: mean silhouette coefficient 0.64, moderate strength (ES ≥ 1.0) and performance differences (ES ≥ 1.1)
Rosso et al. [40]	Elite Paralympic cross country sit skiers	*N* = 15 (10 M, 5 F; 30 ± 6 y)	Balance perturbations *E:* Motion capture system, motorised plate, electromechanical servo actuator *OM:* Delay (ms) between onset of sledge acceleration and shoulder acceleration; delay (ms) between onset of shoulder acceleration and time when trunk inverted the motion; trunk angle (◦) before perturbation; trunk ROM (◦) 150 ms after onset of shoulder acceleration; trunk ROM (◦) when trunk inverted the motion	k-means analysis divided athletes into 2 clusters (high and low impact of impairment) *Temporal variables* Delay between onset of shoulder acceleration and time when trunk inverted the motion was significantly longer for those with high impact of impairment for both forward (*r* = 0.77, *p* = 0.003) and backward (*r* = 0.64, *p* = 0.01) perturbations *Kinematic variables* Trunk angle before perturbation and trunk ROM when trunk inverted the motion were significantly greater for those with high impact of impairment compared with those with low impact of impairment in both forward (*r* = 0.59, *p* = 0.02) and backward directions (*r* = 0.74, *p* = 0.004)
Vanlandewijck et al. [41]	International WC track athletes with normal arm strength	*N* = 13 (10 M, 3 F; 25.6 ± 6.6 y)	**Impairment** (i) Arm isometric strength *E:* Load cell *OM:* Force in Newtons (ii) Trunk isometric strength *E:* Load cell *OM:* Force in Newtons, relative trunk strength (ratio of trunk/arm strength) **Performance** (i) WC acceleration track test *E:* Cheetah LMT *OM:* Distance (m) covered at 1, 2, 3 s (ii) WC acceleration ergometer test *E:* WC ergometer, camera *OM:* Distance (m) covered at 1, 2, 3 s	Participants will full trunk function had significantly greater relative trunk strength (*p* = 0.02) than those with partial trunk function No significant difference found for distance covered on ergometer or track between participants with full or partial trunk function Correlation between trunk strength and WC acceleration on track were low (*r* = 0.27–0.32) and non-significant Correlation between trunk strength and WC acceleration on ergometer were non-significant but almost double that of track performance (*r* = 0.41–0.54)

*ANI* athletes with neurological impairment, *CG* control group, *CV* coefficient of variation, *F* female, *ICC* intraclass correlation coefficient, *IMP* impaired muscle power, *IWRF* International Wheelchair Rugby Federation, *M* male, *max* maximum, *MMT* manual muscle testing, *NDR* non-disabled runners, *NR* not reported, *ROM* range of motion, *RBI* runners with brain injury, *s* seconds, *SEM* standard error of the mean, *TIC* Trunk Impairment Classification, *WC* wheelchair, *y* years

#### Isometric Strength

Isometric strength using a load cell were the most reported measures. The effectiveness of such measures in evaluating strength impairment is supported by multiple studies. A cluster analysis of various isometric strength tests successfully identified wheelchair track racing athletes with comparable amounts of activity limitation [33]. In a comparison between wheelchair rugby players and able-bodied participants, wheelchair rugby players were found to generate less force across all isometric strength measures providing evidence for the discriminant validity of the testing battery [38]. Similarly, a novel battery of three lower limb isometric tests were able to distinguish between runners with a brain injury and non-disabled runners [32]. Furthermore, a study by Hogarth et al. [18] evaluated the predictive validity of strength tests in identifying individuals with strength impairment using a random forest algorithm and found high classification accuracy for both male (96%) and female (94%) Para swimmers with physical impairments. Although various isometric strength measures have demonstrated high test–retest reliability in non-disabled comparison groups (intraclass correlation coefficient [ICC]: 0.85–0.97 [18], 0.97–0.99 [38]), the reliability of such measures in Para sport athletes is yet to be determined.

In relation to associations with sport performance, Para alpine skiers exhibited a significant association between isometric trunk strength and a simulated skiing test [37]. Similarly, a significant association was found between isometric trunk strength and wheelchair propulsion acceleration performance in wheelchair rugby players [31], while combined arm-trunk strength, trunk flexion and arm extension strength correlated with top speed in elite wheelchair racers [33]. However, an earlier study found no significant relationship between trunk strength and acceleration performance in wheelchair track athletes [41]. Studies assessing upper limb strength in wheelchair rugby players found all isometric strength measures significantly correlated with sprint times [39]. Dominant shoulder and hip flexion strength significantly correlated with swim speed in Para swimmers with hypertonia, while non-dominant and dominant shoulder extension strength were the only measures found to significantly correlate with swim speed in Para swimmers with impaired muscle power [18]. Two studies assessed isometric strength in the lower limbs. One revealed a significant association between isometric leg strength measures, race speed and sprint power in Para cyclists [36]. However, the other study concluded that performance in runners with a brain injury was more affected by strength imbalances between limbs rather than the severity of impairment [32]. It is important to note that participants in this study had mild impairments, suggesting the need to investigate athletes with more severe impairments to determine if strength is a limiting factor in the running performance of athletes with brain injuries.

#### Grip Strength

A cluster analysis of wheelchair track racers supported the validity of grip strength as a measure of strength impairment because variations in grip strength were observed between different classes [33]. Grip strength was shown to significantly correlate with 0–15 m speed (*r* = 0.70) and absolute top speed (*r* = 0.79) in elite wheelchair racers [33]. Additionally, correlations with grip strength and hand release speed were also found during seated throwing (*r* = 0.59–0.77), which suggests a potential loss of strength among Para athletes participating in wheelchair sports [35]. However, the limited number of studies evaluating grip strength as a measure of impairment makes it challenging to establish its relationship with performance.

#### Trunk Stability

Regarding the assessment of trunk stability, no significant differences in static balance were found in wheelchair basketball and rugby players when using an ordinal scale Trunk Impairment Classification (TIC) system [30]. However, a significant difference was found between isometric trunk muscle strength and TIC scores in all directions [30]. Furthermore, sit skiers with lower levels of impairment exhibited greater trunk stability during perturbations [40]. Nevertheless, the studies did not investigate the reliability of trunk stability or associations with performance.

### Coordination Impairment Measures

The methods identified for assessing coordination impairments are outlined in Table 2. These include tapping tasks, finger-nose test, repetitive movement tests (RMTs), box and ball test, and an upper limb coordination test using accelerometers. Five studies examined the validity of the method to measure impairment [19, 42–44, 46], three studies investigated the reliability of measures [19, 45, 46], and three studies examined the association between impairment and performance [19, 43, 45].

**Table 2:** Coordination impairment measures

Study	Population	Sample size	Assessments Equipment (E), outcome measure (OM)	Key findings
Altmann et al. [42]	(i) WC rugby athletes with coordination impairment (ii) Volunteers without impairments	*N* = 62 (50 M, 12 F) WC Rugby: *n* = 42 (37 M, 5 F, 16–56 y) Volunteers: *n* = 20 (13 M, 7 F; 18–59 y)	(i) Spiral test *E:* Pen, spiral test form, video camera *OM:* Time (s) to complete movement. Penalty: + 3 s each time line was touched, + 5 s each time line was crossed (ii) Finger-nose test *E:* Video camera *OM:* Number of correct repetitions performed in 20 s (iii) Repetitive movement tests *E:* Video camera *OM:* Number of correct repetitions performed in 20 s (iv) Coordination deduction *OM:* Maximum arm score minus allocated arm score	Correlations between spiral test and repetitive movement tests ranged from 0.40 to 0.67 Correlations between finger-nose test and repetitive movement tests ranged from 0.12 to 0.31 Correlations between coordination deduction and repetitive movement tests ranged from 0.31 to 0.53 All repetitive movement tests had high test accuracy with 95.3–100% of all participants correctly classified as having or not having an impairment
Connick et al. [43]	Participants competed regularly in running events or in a sport where running speed was a determinant of performance	*N* = 41 (M) RBI: *n* = 13 24.3 ± 9.4 y NDR: *n* = 28 23.1 ± 4.1 y	**Impairment** (i) Reciprocal unilateral tapping with 5-cm target (ii) Reciprocal unilateral tapping with 12-cm target (iii) Reciprocal bilateral tapping *E:* 17.5 × 12-cm fibreglass printed circuit boards *OM:* Mean movement time (s) **Performance** 60-m maximal sprint (i) 0–15 m acceleration phase (ii) 30–60 m maximal velocity phase *E:* Cheetah LMT *OM:* Time	RBI mean movement times were significantly slower than NDR for all coordination measures No significant correlation between any of the coordination measures and sprint performance for RBI and NDR
Hogarth et al. [19]	(i) Para swimmers with a brain injury who had a national or international classification, undertaking planned training regimes (ii) Non-disabled participants who were recreationally active	*N* = 51 (31 M, 20 F) Para swimmers: *n* = 21 (16 M, 28.2 ± 6.8 y; 5 F, 20.0 ± 4.5 y) Non-disabled: *n* = 30 (15 M, 23.5 ± 4.1 y; 15 F, 23.3 ± 4.5 y)	**Impairment** (i) Bilateral upper limb tapping (ii) Dominant and non-dominant upper limb tapping (iii) Bilateral lower limb tapping (iv) Dominant and non-dominant lower limb tapping *E:* Custom made wireless tapping pads with 19.5-cm × 10-cm target, video camera *OM:* Mean movement time (ms), lower limb symmetry score (ratio of dominant limb tapping to non-dominant limb tapping) **Performance** *(Para swimmers only)* 10-m maximal freestyle swim *E:* Video camera *OM:* Speed measured in m s^−1^	Non-disabled participants had faster mean tapping times and higher symmetry scores than Para swimmers for all tasks Bilateral upper limb (*r* = − 0.21, *p* = 0.37), upper limb symmetry (*r* = − 0.36, *p* = 0.15) and lower limb symmetry (*r* = − 0.15, *p* = 0.55) were the only measures that did not significantly correlate to swim performance All other measures had moderate to large correlations (*r* = 0.54–0.72) with maximal swim speed 96% of all participants were successfully classified using random forest algorithm *Reliability (non-disabled only)* Upper limb: ICCs were 0.85, 0.91, 0.85, 0.42; CV% were 5.4, 4.2, 5.2, 4.1; SEM were 5, 8, 11, 0.04 ms for bilateral, dominant, non-dominant, upper limb symmetry, respectively Lower limb: ICCs were 0.93, 0.93, 0.91, 0.54; CV% were 5.4, 4.6, 5.4, 4.0; SEM were 8, 14, 16, 0.04 ms for bilateral, dominant, non-dominant, lower limb symmetry, respectively
Maia et al. [44]	(i) Para athletes with hypertonia who had a national or international classification within their sport (ii) Non-disabled participants free from injury	*N* = 38 (26 M, 12 F) Para athletes: *n* = 19 (14 M, 26.6 ± 5.5 y; 5 F, 22.2 ± 6.1 y) Non-disabled: *n* = 19 (12 M, 24.8 ± 5.7 y; 7 F, 30.6 ± 3.9 y)	Motor coordination test *E:* Triaxial accelerometer, digital metronome, video camera *OM:* Smoothness of movement: total number of acceleration peaks over final 20 s Rhythm error: discrepancy between expected and actual timing at end of each arm cycle Movement accuracy: mean absolute distance (in zones) from the reference zone (zone touched with first touch)	Significantly higher number of acceleration peaks found for Para-athletes compared with non-disabled participants during cyclic movements at 30 bpm and 120 bpm (*p* < 0.001) Significantly greater discrepancy in timing reported for Para-athletes compared with non-disabled participants during cyclic movements at 30 bpm (*p* = 0.008) and 120 bpm (*p* < 0.001) No significant difference found for absolute accuracy between Para-athletes and non-disabled participants at either movement frequency 89% of Para-athletes classified successfully using random forest model
Reina et al. [45]	CP footballers from 25 national teams	*N* = 259 (sex NR, 25.46 ± 6.15 y)	**Impairment** (i) Rapid heel-toe contacts E: Tapping platform (35 × 20-cm) OM: Time (s) to complete 25 correct cycles **Performance** Match load E: GPS units OM: Mean/maximum velocity (km h^−1^), total distance covered (m min^−1^), distance covered at different intensities (m min^−1^), accelerations, decelerations (number min^−1^)	FT1 players recorded the slowest performances and FT3 players the fastest Significant differences were found between sport classes for dominant heel-toe contacts (F (2209) = 3.87, *p* = 0.022) Significant correlation between non dominant heel toe and low intensity running during match play (*r* = − 0.51, *p* = 0.029) *Intra-session reliability* ICCs for dominant heel-toe contact was 0.84 and 0.91 for non-dominant leg, indicating good-to-excellent reliability
Roldan et al. [46]	(i) Recreational boccia players classified as BC1 or BC2 competing in regional or national competition (ii) Adults without any physical impairments	*N* = 73 boccia players with CP (42 M, 31 F) BC1: *n* = 33 (34.01 ± 16.43 y) BC2: *n* = 40 (33.97 ± 14.29 y) *N* = 19 non-disabled controls (sex NR, 27.89 ± 7.08 y)	(i) Box and block test (ii) Box and ball test *E:* Box and blocks/boccia balls, video camera, stopwatch *OM:* Number of blocks/balls moved in a minute Tapping tasks: (i) Discrete horizontal finger tapping test (ii) Discrete vertical finger tapping test *E:* Metal plates (30 × 20-cm) with target area (18 × 5 cm) *OM:* Average score of 10 tapping cycles (s) (iii) Discrete vertical tapping test with ball *E:* Metal plates (14 × 17-cm) that worked on spring system *OM:* Average score of 10 tapping cycles (s) (iv) Continuous vertical tapping test with ball *E:* Metal plates (14 × 17-cm) that worked on spring system *OM:* Number of contacts made in a minute	Non-disabled controls performed significantly better on all tests compared with boccia players A large significant correlation was found between the box and block tests and box and ball test (*r* = 0.80, *p* < 0.01) Moderate to very large negative correlations were found between discrete tapping tasks, box and block test and box and ball test (− 0.30 < *r* < − 0.75; 0.05 > *p* < 0.01) A large significant correlation was found between the continuous tapping test, box and block test and box and ball test (0.59 < *r* < 0.66; 0.05 > *p* < 0.01) Significant differences found between boccia classes for box and block test, box and ball test and continuous vertical tapping test Both tapping tests with ball displayed good intrasession reliability; discrete (ICC = 0.87), continuous (ICC = 0.88)

*CP* cerebral palsy, *F* female, *M* male, *NDR* non-disabled runners, *NR* not reported, *RBI* runners with brain injury, *s* seconds, *WC* wheelchair, *y* years

#### Tapping Tasks

Tapping tasks were the most reported coordination measure and were evaluated in four studies across different Para sports (swimming, boccia, athletics, CP football). In terms of their validity for measuring impairment, 96% of non-disabled and Para swimmers were successfully classified using a random forest algorithm [19]. Additionally, a continuous vertical tapping test was able to differentiate athletes from two boccia sport classes [46].

Three of the four studies investigated the association between tapping tasks and performance with differing results observed. One study found no significant relationship between three tapping tasks and sprint performance in runners with a brain injury [43]. The lack of association may be attributed to the mild impairments of participants or the impairment-specific designs of the assessments [43]. However, a significant moderate-to-strong relationship using the same tapping equipment was found between bilateral lower limb, unilateral upper/lower limb tapping tasks and maximal freestyle swim speed in Para swimmers with brain injuries [19]. Similarly, Reina et al. [45] found a significant association between non-dominant rapid heel toe reciprocal tapping, and distance covered at low-intensity running in CP footballers during match play.

In terms of reliability, the intra-session reliability of heel toe reciprocal tapping was found to be strong in CP footballers, with ICC values of 0.84 and 0.91 for dominant and non-dominant legs, respectively, indicating good test reproducibility [45]. Similarly, in boccia players both tapping tasks with a ball exhibited good intra-session reliability (discrete tapping test: ICC 0.87, continuous tapping test: ICC 0.88) [46]. However, the test–retest reliability of tapping tasks was only evaluated in a single study and assessed in a non-disabled comparison group [19].

#### Spiral, Finger-Nose and Upper Limb RMT, Accelerometers, and Box and Ball Test

To assess the construct validity of newly developed RMT for measuring arm coordination impairment in wheelchair rugby, the clinically validated spiral and finger-nose tests were used [42]. The moderate correlations (*r* = 0.40–0.67) between the spiral test and RMT supported the RMT’s construct validity [42]. Furthermore, the high-test accuracy (95.3–100%) of the RMT in correctly classifying participants as having or not having an impairment provided additional evidence of its validity [42].

Similarly, to enhance the objectivity of Para swimming classification, the use of accelerometers to measure rhythm error, movement smoothness and accuracy in athletes with hypertonia was investigated [44]. A random forest algorithm successfully classified 89% of Para swimmers and 100% of non-disabled participants, suggesting that measures of rhythm error and movement smoothness could be utilised to infer impairment in swimmers with hypertonia [44]. Additionally, the box and block/ball test was able to discriminate between non-disabled participants and boccia athletes from two different classification classes [46]. However, these measures have only been evaluated in individual studies, with none exploring the reliability of these measures or associations with performance.

### ROM Impairment Measures

Table 3 outlines the methods for measuring ROM impairment, which include an inclinometer, goniometer, segmometer and motion analysis system. Three studies examined the validity of the method to measure impairment [43, 47, 48], one study examined reliability [48] and two studies examined associations with performance [37, 43].

**Table 3:** ROM impairment measures

Study	Population	Sample size	Assessments Equipment (E), outcome measure (OM)	Key findings
Bjerkefors et al. [47]	(i) Para kayakers competing at national or international level with ≥ 1 year experience (ii) Elite able-bodied kayakers	*N* = 51 (17 F, 34 M) Para-kayakers: *n* = 41 (28 M, 13 F; 35 ± 9 y) Able-bodied: *n* = 10 (6 M, 24 ± 3 y; 4 F, 20 ± 1 y)	Kinematic analysis of kayak performance *E:* Kayak ergometer, motion capture system, force transducer *OM:* ROM or max/min peak angle were recorded for shoulder, elbow, wrist, trunk, hip, knee and foot	Significantly higher stroke frequency found in able-bodied (*p* = 0.02) and KL3 athletes (*p* = 0.046) when compared with KL1 athletes (most impaired) Able-bodied athletes demonstrated significantly larger trunk, pelvis and lower limb angles than Para kayakers Able-bodied athletes and least impaired Para kayakers displayed greater trunk flexion and smaller trunk extension than more impaired athletes (KL1) Greater shoulder ROM and extension found in more impaired Para-athletes compared with least impaired/able-bodied athletes
Connick et al. [43]	Participants competed regularly in running events or in a sport where running speed was a determinant of performance	*N* = 41 (M) RBI:* n* = 13 24.3 ± 9.4 y NDR: *n* = 28 23.1 ± 4.1 y	**Impairment** (i) Maximum thigh flexion and heel pull distance *E:* Inclinometer, segmometer *OM:* Angle b/w thigh and horizontal. Distance on non-tested leg b/w wall and position of heel on tested leg (ii) Maximum thigh extension *E:* Inclinometer *OM:* Angle b/w tested thigh and vertical (iii) Dorsiflexion lunge *E:* Inclinometer *OM:* Angle b/w tested tibia and horizontal (iv) Backward stepping lunge *E:* Segmometer *OM:* Distance b/w most anterior phalanx of back foot and heel of front foot **Performance** 60-m maximal sprint (i) 0–15 m acceleration phase (ii) 30–60 m maximal velocity phase *E:* Cheetah LMT *OM:* Time (s)	RBI had lower ROM compared with NDR in all measures but only found to be significant for dorsiflexion lunge and heel pull distance on best and worse legs Five ROM measures significantly correlated to RBI sprint performance (convergent validity) but did not significantly correlate to NDR sprint performance (divergent validity): maximum thigh flexion (best leg), heel pull distance (best and worse legs), maximum thigh extension (best leg), dorsiflexion lunge (best leg) RBI had significantly lower acceleration (3.2 s ± 0.3 s vs 2.8 s ± 0.2 s) and top speed times (4.3 s ± 0.6 s vs 3.8 s ± 0.3 s) compared with NDR
Hyde et al. [35]	WC rugby, WC basketball and seated throw athletes (athletics) who had an eligible impairment, were currently training and competing in their respective Paralympic sport	*N* = 10 (8 M, 2 F; 32 ± 10 y)	Kinematic analysis of seated throwing performance *E:* Motion capture system *OM:* ROM measured in degrees. Angular velocity measured in m s^−1^	Kinematic variables were not affected by seat configuration Use of assistive pole: greater hand release speed, greater maximum shoulder external-rotation velocity during cocking phase, less elbow flexion
Liu et al. [37]	Para alpine skiing athletes who had a minimum of 6 months experience and an official classification	*N* = 38 (27 M, 11 F; 23.5 ± 5.4 y)	**Impairment** Trunk ROM (forward flexion, backward extension) *E:* Video camera, goniometer *OM:* ROM measured in degrees **Performance** Board tilt test (simulated skiing test) *E:* Horizontal test board and two rolling curved boards *OM:* Maximal board tilt angle (^◦^)	Significant correlation found between trunk ROM and board tilt test (*r* = 0.811, *p* < 0.001)
Nicholson et al. [48]	(i) Para swimmers who had a national or international classification, undertaking planned training regimes and competing at a national or international level (ii) Non-disabled participants who were recreationally active	*N* = 66 (37 M, 29 F) Para swimmers: *n* = 24 Hypertonia: *n* = 11 (9 M, 2 F; 27 ± 5.7 y) IMP: *n* = 13 (8 M, 5 F; 30 ± 6.4 y) Non-disabled: *n* = 42 (20 M, 22 F; 23.2 ± 4.5 y)	(i) Bilateral shoulder flexion *E:* Inclinometer *OM:* ROM measured in degrees (ii) Bilateral shoulder abduction *E:* Inclinometer *OM:* ROM measured in degrees (iii) Elbow flexion and extension *E:* Inclinometer, goniometer *OM:* ROM measured in degrees (iv) Lower limb streamline (hip, knee, ankle extension) *E:* Inclinometer *OM:* ROM measured in degrees (v) Hip and knee flexion *E:* Inclinometer, goniometer *OM:* ROM measured in degrees (vi) Shoulder internal and external rotation *E:* Inclinometer *OM:* ROM measured in degrees (vii) Prone shoulder extension *E:* Inclinometer *OM:* ROM measured in degrees (viii) Prone shoulder horizontal abduction *E:* Inclinometer *OM:* ROM measured in degrees (ix) Prone shoulder flexion *E:* Inclinometer *OM:* ROM measured in degrees (x) Trunk functional reach (forwards, backwards, sideways) *E:* Tape measure *OM:* Distance between starting and finishing position of acromion	ROM was significantly different between Para swimmers and non-disabled participants for most of the tests Swimmers with hypertonia were found to have significantly greater ROM than swimmers with IMP for right hip flexion, left and right knee flexion, left shoulder internal rotation, functional reach (all directions) *Intra-examiner reliability (non-disabled only):* ICCs ranged from 0.66 to 0.98, SEM ranged from 1.18 to 6.29^◦^ For all measures other than left elbow extension (*p* = 0.04), lower limb streamline left ankle (*p* < 0.01) and left knee flexion with goniometer (*p* = 0.04), no significant difference were found between trials *Inter-examiner reliability (non-disabled only)* ICCs ranged from 0.62 to 0.98, SEM ranged from 0.73 to 6.52^◦^ Significant differences were found between examiners for: Right elbow flexion and extension (goniometer), left elbow flexion (inclinometer and goniometer), left hip flexion, left knee flexion (goniometer), left and right shoulder internal rotation

*b/w* between, *F* female, *IMP* impaired muscle power, *M* male, *NDR* non-disabled runners, *RBI* runners with brain injury, *ROM* range of motion, *s* seconds, *WC* wheelchair, *y* years

#### Inclinometer, Goniometer and Segmometer

Regarding the assessment of ROM using an inclinometer and goniometer, Para swimmers exhibited significantly reduced ROM compared with non-disabled participants [48]. However, the reliability of the measures was only assessed in the non-disabled comparison group, with inter-rater reliability shown to be better when using an inclinometer (ICC range 0.75–0.98) compared with a goniometer (ICC range 0.21–0.79) [48]. Furthermore, measures of ROM using an inclinometer and segmometer were found to be valid, as five ROM measures were found to significantly correlate with sprint performance in runners with a brain injury (convergent validity) but not with sprint performance in non-disabled runners (divergent validity) [43].

#### Motion Analysis System

Kinematic analysis using a motion analysis system showed significant differences between non-disabled and Para kayakers in trunk/pelvis rotation, hip flexion ROM, knee flexion ROM and ankle flexion ROM [47]. A significant association was also found between these variables and power output when paddling [47]. However, no significant differences for seat configuration (with and without a pole) on any of the kinematic variables during seated throwing were found [35].

### Intellectual Impairment Measures

Table 4 outlines the methods identified for measuring intellectual impairments. These include a series of generic cognitive tests, reaction time assessments, executive function measures, a multiple object tracking test (MOT) and cognitive-motor dual tasking.

**Table 4:** Intellectual impairment measures

Study	Population	Sample size	Assessments Equipment (E), outcome measure (OM)	Key findings
Pineda et al. [49]	(i) High-level athletes with II who participated in sports such as athletics, basketball, cycling, swimming, table tennis and tennis (ii) CG of athletes, matched by age, sex, sport practiced, and lifetime accumulated training hours	*N* = 58 Athletes with II: *n* = 29 (24 M, 5 F; 25.4 ± 6.0 y) CG: *n* = 29 ( 24 M, 5 F; 24.3 ± 6.2 y)	Cognitive postural dual tasking (i) Recognition task *E:* Computer with images presented on white background for 0.5 s *OM:* Proportion of correct responses (%) (ii) Postural task *E:* Rocking board laid on force plate *OM:* COP trajectory (mm)	CG athletes performed significantly better than athletes with II in both single cognitive performance test and dual task cognitive performance (*p* < 0.001) Athletes with II prioritised maintaining balance during multi-tasking, decreasing their cognitive performance
Van Biesen et al. [50]	(i) Athletes with II who competed in athletics, basketball, cycling, soccer, swimming, and tennis (ii) Belgian tertiary education students	*N* = 206 Athletes with II: *n* = 103 (70 M, 33 F; 24.4 ± 5.8 y) Students: *n* = 103 (70 M, 33 F; 21.1 ± 2.4 y)	(i) Simple Reaction Time *E:* Computer, keyboard *OM:* Mean reaction time (ms) from 12 trials (ii) Choice Reaction Time *E:* Computer, keyboard *OM:* Number of responses in 30 s (iii) Adapted Flanker Task *E:* Computer, keyboard *OM:* Number of responses in 30 s	Moderate negative correlation between IQ and: Simple Reaction Time (*r* = − 0.34, *p* < 0.001) Choice Reaction Time (*r* = − 0.46, *p* < 0.001) Adapted Flanker Task (*r* = − 0.49, *p* < 0.001) Athletes with II displayed significantly slower reaction times across all three assessments when compared to students (*p* < 0.001) Significant main effect was found for task, with reaction time increasing with increasing cognitive load *F*(1, 204) = 1610.7, *p* < 0.001, *η*^2^ = 0.94 Significant main effect was found between groups, with athletes with II performing worse in all three tasks compared to students *F*(1, 204) = 163.59, *p* < 0.001, *η*^2^ = 0.44
Van Biesen et al. [51]	Well-trained table tennis players with ID	*N* = 88 (59 M, 29 F; 27.5 ± 8.4 y)	**Impairment** GCT: (i) Simple Reaction Time *E:* Computer, keyboard *OM:* Mean reaction time (ms) from 12 trials (ii) Complex Reaction Time *E:* Computer, keyboard *OM:* Mean reaction time (ms) from 28 trials (iii) Simple Visual Search, Complex Visual Search *E:* Computer, touch screen *OM:* Mean reaction time (ms) from 12 trials (iv) Corsi Memory Test *E:* Computer, touch screen *OM:* Best average score from 5 successive trials (v) Tower of London *E:* Computer, touch screen *OM:* Number of correct items (max 18) (vi) Block Design Test *E:* 9 × 3D cubes *OM:* Depends on correct items and speed (max 72) (vii) Matrix Reasoning Test *E:* NR *OM:* Number of correct items (max 35) (viii) Finger Tapping *E:* Computer, keyboard *OM:* Max number of taps per 10 s **Performance** (i) Tactical Proficiency Test (Tactic 1) *E:* Video camera *OM:* Average score obtained over 12 sets (ii) Tactical Proficiency test (Tactic 2) *OM:* Score obtained from 12 rallies	No significant correlation between IQ score, GCT or training history *Tactic 1* Backward stepwise multiple regression model was not significant, with simple reaction time found to be the only significant predictor (β = − 0.18, *p* < 0.05) *Tactic 2* Backward stepwise regression model showed that 18% of the variation in tactical proficiency could be explained by the block design and simple reaction time test
Van Biesen et al. [52]	(i) Well trained athletes in the sports of athletics, basketball, swimming, and table tennis with ID (ii) Well trained athletes in the sports of athletics, basketball, swimming, and table tennis without ID	*N* = 630 Athletes with ID: *n* = 468 (317 M, 151 F; 24.1 ± 6.3 y) Athletes without ID: *n* = 162 (107 M, 55 F; 23.3 ± 5.1 y)	GCT: (i) Simple reaction time *E:* Computer, keyboard *OM:* Mean reaction time (ms) from 12 trials (ii) Complex reaction time *E:* Computer, keyboard *OM:* Mean reaction time (ms) from 28 trials (iii) Simple Visual Search, Complex Visual Search *E:*Computer, touchscreen *OM:* Mean reaction time (ms) from 12 trials (i) Corsi Memory Test *E:* Computer touch screen *OM:* Best average score from 5 successive trials (i) Tower of London *E:* Computer, touch screen *OM:* Number of correct items (max 18) (i) Block Design Test *E:* NR *OM:* Depends on correct items and speed (max 72) (ii) Matrix Reasoning Test *E:* NR *OM:* Number of correct items (max 35) (i) Finger Tapping *E:* Computer, keyboard *OM:* Max number of taps per 10 s	After adjusting for psychomotor speed, athletes without ID scored significantly higher than athletes with ID in all GCT except Complex Visual Search (*p* < 0.05) *Test–retest reliability (Athletes with ID)* High reliability was found for Matrix Reasoning (*r* = 0.81) and Block Design tests (*r* = 0.88) Acceptable reliability was found for Simple Reaction Time (*r* = 0.71), Complex Reaction Time (*r* = 0.66), Simple Visual Search (*r* = 0.67) and Corsi Memory Test (*r* = 0.76) Low reliability found for Tower of London (*r* = 0.48) and Complex Visual Search (*r* = 0.25)
Van Biesen et al. [53]	(i) Athletes with II who competed in sports of athletics, basketball, swimming, soccer, cycling and tennis (ii) Athletes without II (CG)	*N* = 206 Athletes with II: *n* = 103 (70 M, 33 F; 24.4 ± 5.8 y) CG: *n* = 103 (70 M, 33 F; 22.0 ± 2.4 y)	Adapted Multiple Object Tracking Test *E:* Computer *OM:* Total number of correct trials (max 15)	Significantly worse performance for athletes with II (*p* < 0.001) with moderate effect size compared with CG when controlling for age Significant main effect was observed between groups when performing Multiple Object Tracking Test while balancing on one leg. The CG demonstrated significantly better performance (*F*(1, 199) = 131.67, *p* < 0.001, *η*^2^ = 0.40) Athletes with II exhibited greater performance decreases on the Multiple Object Tracking Test when balancing on one leg compared with CG (*F*(1, 199) = 10.43, *p* < 0.001, *η*^2^ = 0.05)
Van Biesen et al. [54]	(i) Athletes with ID who have competed at international level (ii) Athletes without ID who have competed at local or national level	*N* = 59 Athletes with ID: *n* = 29 (20 M, 9 F; 25.5 ± 5.5 y) Athletes without ID: *n* = 30 (21 M, 9 F; 23.8 ± 5.4 y)	Executive function (i) Adapted Flanker Test *E:* Computer, keyboard *OM:* Number of correct and incorrect taps made in 30 s (ii) Color Trails Test *E:* Testing sheet *OM:* Time (s) to complete (iii) Updating World Span Task *E:* NR *OM:* Point given for each correctly recalled item in correct sequence (max 30) Intellectual Function (IQ) (i) Wechsler Adult Intelligence Scale-III (Dutch version)	Significant difference was found in executive function performances between groups (*p* < 0.001), with athletes with ID exhibiting lower performance scores Stronger relationship between IQ and Flanker Test in ID athletes compared to athletes without ID (*p* = 0.008) No significant difference between the relationship of IQ and other executive function measures between athletes with and without ID (*p* = 0.337*)* The relationship between Flanker Test and Updating World Span Task is stronger for athletes with ID than those without ID (*p* = 0.042) The relationship between Flanker Test and Color Trail Test 1 is stronger for athletes with ID than those without (*p* = 0.023)

*3D* three-dimensional, *CG* control group, *ID* intellectual disability, *II* intellectual impairment, *IQ* Intelligence Quotient, *GCT* generic cognitive tests, *max* maximum, *NR* not reported, *s* seconds, *y* years

#### Generic Cognitive Tests

Generic cognitive tests refer to a subset of tests that examine speed, short-term memory, executive function, fluid reasoning, visual processing and psychomotor performance [51, 52]. After controlling for differences in psychomotor speed, athletes without impairment performed significantly better than those with impairments in all generic cognitive tests except for the Complex Visual Search [52]. High test–retest reliability was found in a subsample of athletes with intellectual impairment for the Matrix Reasoning (*r* = 0.81) and Block Design (*r* = 0.88) tests [52]. However, tests such as the Tower of London and Complex Visual Search demonstrated low test–retest reliability [52], suggesting a need for reconsideration in current classification procedures. In relation to performance, the Block Design and Simple Reaction Time test accounted for 18% of tactical proficiency when evaluating service, return and rally skills among table tennis players with intellectual impairment [51].

#### Reaction Time and Executive Function Measures

In a study investigating the relationship between intelligence and reaction time, a significant moderate negative correlation was found, suggesting that athletes with higher IQ scores appear to have faster reaction times [50]. When compared with a control group, athletes with intellectual impairment showed significantly slower reaction times and demonstrated poorer performances across all assessed measures [50]. Similarly, executive function measures, including the adapted Flanker test, Color Trails Test and Updating World Span task, showed significant differences between athletes with and without intellectual impairment, with impaired athletes demonstrating lower performance scores [54]. Comparing these measures to IQ scores, a significant difference was observed in the Flanker test for athletes with impairment, while no significant differences were found between the other two measures and IQ scores in both groups [54].

#### MOT and Cognitive-Motor Dual Tasking

The MOT revealed significant performance differences (after adjusting for age) between athletes with impairment and a control group [53]. When incorporated into a dual task scenario with single leg balance, athletes with impairment displayed a significantly greater decline in performance [53]. In a later study investigating cognitive-motor dual tasking, athletes with intellectual impairment displayed lower cognitive performance in both single and dual task conditions compared with a control group [49]. They were found to prioritise balance during multitasking, leading to a decrease in cognitive performance [49].

### Vision Impairment Measures

The methods identified for assessing vision impairments are outlined in Table 5. This section is dedicated to exploring the relationship between impairment measures and associations with performance in athletes with vision impairment, aligning with one of the research models outlined in the joint position stand on sports-specific classification of athletes with vision impairment [78].

**Table 5:** Vision impairment measures

Study	Population	Sample size	Assessments Vision test (VT), outcome measure (OM)	Key findings
Allen et al. [55]	Elite VI shooting athletes	*N* = 25 (16 M, 9 F; 49 ± 11.6 y)	**Impairment** (i) VA *VT:* ETDRS *OM:* Letter by letter scoring (logMAR units) *VT:* BRVT *OM:* NR (ii) Contrast Sensitivity *VT:* Pelli–Robson chart *OM:* Each correctly named letter scored 0.05 logCS *VT:* Mars chart *OM:* Contrast level of the final number minus 0.04 logCS for every incorrectly named number **Performance** 10-m air rifle competition scores (prone and standing) *OM:* Score after qualifying round	*VA* No relationship b/w VA and shooting scores in prone (τ correlation: − 0.22, *p* = 0.14) or standing (τ correlation: − 0.11, *p* = 0.49) *Contrast sensitivity* No significant difference in either prone shooting performance [Pelli Robson: t(23) = 0.72, p = 0.48; Mars: t(23) = 1.00, *p* = 0.33] or standing shooting performance [Pelli Robson: t(20) 0.50, *p* = 0.62; Mars t(20) 1.41, *p* = 0.17] b/w groups with measurable and no measurable contrast sensitivity No significant difference in performance scores of athletes with congenital or acquired vision loss in prone or standing performance
Fortin-Guichard et al. [56]	International level VI swimmers who compete in 100-m freestyle	*N* = 45 (49.8% F; 20.8 ± 6.8 y)	**Impairment** (i) VA *VT:* BRVT *OM:* logMAR units (ii) Contrast Sensitivity *VT:* Mars chart *OM:* Contrast level of the final number minus 0.04 logCS for every incorrectly named number (iii) Light sensitivity *VT:* Mars chart using Brightness Acuity Tester *OM:* Difference in logCS b/w lighting conditions (iv) Depth perception *VT*: Modified Howard–Dolman test *OM*: Mean absolute value from 6 trials (v) Visual search *VT:* Custom made visual search test *OM:* Response time (difficult level completed) (vi) Motion perception *VT:* Custom made computer-based test *OM:* Threshold coherence level (%) **Performance** (i) Best race time *OM:* Fastest time (ii) Start time *OM:* Time from start to 15-m flags (iii) Clean swim velocity *OM:* Average speed (m/s) b/w 15–45 m and 55–95 m (iv) Turn time *OM:* Time taken b/w 45-50 m (v) Finish time *OM:* Time taken to swim through final 5 m (vi) Mean lateral position in lane *OM:* Average absolute distance (cm) from centre of lane	VA was best predictor of total race time (*r* = 0.40, *p* < 0.01), relationship not linear Decision tree analysis suggested two classes for legitimate competition in VI swimming, with a cutoff b/w 2.6 and 3.5 l ogMAR No significant associations found b/w visual function and performance following split into two classes (all |*r*s|< 0.11 and *p*s > 0.54)
Krabben et al. [57]	Elite VI Judokas	*N* = 53 (38 M, 15 F; 27.9 ± 8.8 y)	**Impairment** (i) VA *VT:* BRVT *OM:* LogMAR units (ii) Contrast sensitivity *VT:* MARS Letter Contrast Sensitivity Test *OM:* Score in logCS (calculated as contrast of final number identified correctly minus a correction for single mistakes made prior) (iii) Light sensitivity *VT:* Mars Letter Contrast Sensitivity Test (with and without presence of bright light using Brightness Acuity Tester) *OM:* Percentage loss b/w first and second test (iv) Depth perception *VT:* Custom made modified version of Howard–Dolman test *OM:* Mean absolute error (mm) across four trials (v) Motion perception *VT:* Custom-made computer based test *OM:* NR (vi) Visual search *VT:* Custom made visual search test *OM:* Mean response time (vii) Central visual field *VT:* Berkeley Central Visual Field Test *OM:* Percentage of stimuli seen **Performance** Match result data collected from International Blind Sports Federation website (competitions 2015–20) *OM:* Win/fight ratio	*Vision test battery and performance* Significant correlation found b/w VA and judo performance (*r* = − 0.33, *p* = 0.02) No significant correlation found b/w performance and: Contrast sensitivity (*r* = − 0.09, *p* = 0.69) Depth perception (*r* = 0.21, *p* = 0.30) Motion perception (*r* = − 0.06, *p* = 0.80) Light sensitivity (*r* = − 0.02, *p* = 0.98) Visual search (*r* = − 0.13, *p* = 0.55) Visual field (*r* = 0.19, *p* = 0.40) *Splitting on basis of visual function and performance* Decision tree analysis suggested using cutoff point of approximately 2.6 logMAR No correlations found b/w any of vision tests and performance within two resulting subgroups
Krabben et al. [58]	Elite VI Judokas	*N* = 296 (sex NR, Age > 18 y)	**Impairment** (i) VA *VT:* BRVT *OM:* logMAR units (ii) Visual field *VT:* NR *OM:* degrees radius **Performance** Win ratio of fights won for competitions b/w 2012–18	VA was significantly correlated with judo performance (*r* = − 0.32, *p* < 0.001) No significant relationship b/w visual field and judo performance (*r* = 0.30, *p* = 0.15) Decision tree analysis suggested using cutoff point of approximately 2.5 logMAR
Latham et al. [59]	Elite VI shooting athletes with international competition experience	*N* = 23 (14 M, 9 F; 49 ± 12 y)	**Impairment** (i) VA *VT:* ETDRS chart and/or BRVT *OM:* LogMAR units (ii) Contrast sensitivity *VT:* Mars Chart *OM:* LogCS units (iii) Visual field *VT:* Humphrey Field Analyser *OM:* Points scored within 0–100 for full visual field, central visual field and peripheral visual field **Performance** Competition scores (prone and standing) *OM:* Score per shot at end of qualifying round	Shooting performance in prone or standing was not associated with visual field function (*p* > 0.05) Having measurable visual field function beyond 30^◦^ did not impact athletes’ ability to achieve competitive shooting scores in either prone (*p* = 0.65) or standing events (*p* = 0.47)
Myint et al. [60]	Elite VI shooting athletes	*N* = 10 (sex, age NR)	**Impairment** (i) VA *VT:* ETDRS LogMAR letter chart (DVA) *OM:* Letter by letter scoring measured in LogMAR units *VT:* BRVT *OM:* logMAR units *VT:* SLOAN two-sided ETDRS Format Near Point Test LogMAR reading card (NVA) *OM:* logMAR units (ii) Contrast Sensitivity *VT:* Pelli-Robson chart *OM:* LogCS units (iii) Visual field *VT:* Henson 9000 Field *OM:* Mean defect (dB) **Performance** 10-m air rifle competition scores (prone and standing) *OM:* Score after qualifying round	*DVA* No significant correlation found with standing (*τ*: 0.36, *p* = 0.15) and prone competition score (*τ*: − 0.15, *p* = 0.65) *NVA* No significant correlation found with standing (*τ*: − 0.35, *p* = 0.28) and prone competition score (*τ*: 0.36, *p* = 0.15) *Contrast sensitivity* No significant correlation found with standing (*τ*: − 0.47, *p* = 0.08) and prone competition score (*τ*: 0.33, *p* = 0.34) *Visual field* No significant correlation found with standing (*τ*: 0.09, *p* = 0.72) and prone competition score (*τ*: − 0.55, *p* = 0.09)
Stalin et al. [61]	Para Nordic and Para alpine skiers	*N* = 41 (26 M, 15 F) Para Nordic: *n* = 26 (18 M, 8 F; 26.0 ± 6.3 y) Para alpine: *n* = 15 (8 M, 7 F; 28.1 ± 11.6 y)	**Impairment** *Measures same as those reported by Stalin *et al*. *[62] (i) Static VA (ii) Contrast sensitivity (iii) Glare sensitivity iv) Glare recovery (v) Light sensitivity (vi) Dynamic VA (vii) Translational and radial motion perception (viii) Visual field **Performance** Recalculated World Para Nordic and Para alpine skiing points using raw race times across the season	*Nordic skiers* Clusters 1 and 2 showed larger visual fields (*p* = 0.004) and static VA (*p* = 0.041) compared with cluster 3 *Alpine skiers* The top performing clusters in slalom, giant slalom, super G displayed significantly better average static visual acuities (*p* = 0.019, *p* = 0.019, *p* = 0.039, respectively) than the worst performing cluster The cluster with better performance in slalom had significantly larger visual field (*p* = 0.038) The cluster with better downhill performance demonstrated significantly better dynamic VA (*p* = 0.029)
Stalin et al. [62]	Para Nordic and Para alpine skiers	*N* = 41 (26 M, 15F) Para Nordic: *n* = 26 (18 M, 8 F; 26.0 ± 6.3 y) Para alpine: *n* = 15 (8 M, 7 F; 28.1 ± 11.6 y)	**Impairment** (i) Static VA *VT:* ETDRS and/or BRVT *OM:* logMAR (ii) Contrast sensitivity *VT:* Quick contrast sensitivity procedure on adaptive sensory technology platform *OM:* logCS (iii) Glare sensitivity *VT:* Static VA in presence of bright binocular source in line of sight *OM:* change in logMAR from baseline static VA (iv) Glare recovery *VT:* Static VA 1 min after glare source removed *OM:* change in logMAR from baseline static VA (v) Light sensitivity *VT:* Static VA test in presence of bright light *OM:* change in logMAR from baseline static VA (vi) Dynamic VA *VT:* Computer program with single moving tumbling E letter *OM:* logMAR using a per letter scoring system (vii) Translational and radial motion perception *VT:* Random dot kinematograms *OM:* Threshold calculated by averaging last six reversals (%) (viii) Visual field *VT:* Arc perimeter following standardised protocol *OM:* Modified AMA scoring method (%) **Performance** Recalculated World Para Nordic and Para alpine skiing points using raw race times across the season	No significant correlations found b/w vision impairment measures and performance after Bonferroni-Holm corrections Before corrections were applied, significant correlations found b/w: Para Nordic skiing performance and visual field (*τ*_b_: − 0.37, *p* = 0.011) Downhill Para alpine skiing performance and static VA (*τ*_b_: 0.54, *p* = 0.046) Giant slalom Para alpine skiing performance, static VA (*τ*_b_: 0.50, *p* = 0.010) and contrast sensitivity (*τ*_b_: − 0.46, *p* = 0.017) Super G Para alpine skiing performance, static VA (*τ*_b_: 0.57, *p* = 0.007), contrast sensitivity (*τ*_b_: − 0.51, *p* = 0.017) and translational motion perception (*τ*_b_: 0.49, *p* = 0.041) Slalom Para alpine skiing performance and visual field (*τ*_b_: − 0.49, *p* = 0.013) Static VA was significant predictor of giant slalom, super G and slalom performance (Para alpine skiing)

*b/w* between, *BRVT* Berkeley Rudimentary Vision Test, *DVA* distance visual acuity, *ETDRS* Early Treatment Diabetic Retinopathy Study, *F* female, *M* male, *min* minute, *NVR* near visual acuity, *VA* visual acuity, *VI* vision impairment, *y* years

#### Visual Acuity

Visual acuity measures did not display significant associations with shooting performance in both standing and prone events, suggesting that one class is necessary for competition [55, 60]. Regarding judo performance, visual acuity emerged as the strongest predictor and remained the only visual function associated with performance when accounting for correlations between visual acuity and other visual functions [57]. Initial findings, prior to Bonferroni–Holm corrections, indicated significant associations between static visual acuity and Para alpine skiing performance in the downhill, giant slalom and super G events [62]. In swimming, visual acuity demonstrated significant associations with best race time, start time, finish time and mean lateral position in the lane [56]. These associations were observed both with the exclusion of missing values and inclusion of dummy values [56]. Furthermore, visual acuity emerged as the primary predictor of total race time in swimming [56]. Static visual acuity significantly predicted Para alpine skiing performance in the giant slalom, super G and slalom events [62].

#### Visual Field

In the assessment of visual field, judo performance within a subsample of 25 Judokas showed no significant associations [58]. Similarly, judo performance did not show any significant associations with visual field, both before and after applying cutoff points following a decision tree analysis, where the data were split into two groups (i.e. ≤ 2.6 logMAR or > 2.6 logMAR) [57]. Investigations into the relationship with shooting performance found no significant correlation between visual field function and performance in prone or standing events, emphasising that the presence or absence of peripheral vision did not impact an athlete’s ability to shoot competitively in either event [59, 60]. Before applying Bonferroni–Holm corrections, a significant association was found between visual field and Para Nordic skiing performance, as well as with Para alpine skiing performance in the slalom event [62]. Furthermore, a cluster analysis revealed that clusters with better performances demonstrated significantly larger visual fields in Para Nordic skiing and the slalom event in Para alpine skiing [61].

#### Contrast and Light Sensitivity

No significant difference between groups with and without measurable contrast sensitivity in both prone and standing performance were found [55], while another study investigating contrast sensitivity and shooting performance showed no significant correlation between prone and standing events, suggesting that there is no need for more than one class [60]. Similarly, no significant associations were found between contrast sensitivity, light sensitivity and judo performance, both before and after the application of cutoff points from the decision tree analysis [57]. Initially, giant slalom and super G Para alpine skiing performance significantly correlated with contrast sensitivity, but after Bonferroni–Holm corrections, no significant associations were found [62]. In swimming performance, significant correlations were found between contrast sensitivity, finish time and mean lateral position in the lane, with both missing values excluded, and dummy values included [56]. The inclusion of dummy values also revealed a significant association between light sensitivity, start time, finish time and mean lateral position in the lane [56].

#### Depth and Motion Perception

There was no significant association found between depth and motion perception and judo performance, before and after applying cutoff points [57]. This lack of association may be due to the initial contact between athletes at the start of the match [57]. Prior to Bonferroni–Holm corrections, a significant relationship was found between translational motion perception and super G Para alpine skiing performance [62].

### Activity Limitation Measures

Table 6 outlines the studies that have primarily focused on the evaluation of sport-specific activities that could be used in evidence-based classification to quantify the extent of activity limitation resulting from impairment, addressing step 3b of the research guidelines [6]. For ease of analysis and to account for the sport-specific nature of the tests, this section has been categorised by sport, with twelve studies focusing on CP football, two on wheelchair rugby, three on Para swimming, one on athletics and one on table tennis.

**Table 6:** Activity limitation measures

Study	Population	Sample size	Assessments Equipment (E), outcome measure (OM)	Key findings
Altmann et al. [23]	WC rugby and WC basketball athletes with ≥ 1 year experience	*N* = 55 (sex NR, 34 ± 10 y)	(i) 10-m sprint (ii) Turn test *E:* Infrared sensors *OM:* Time (s) (iii) Tilt test *E:* Tape measure attached to pulley system *OM:* Difference between maximum and initial height (mm) (iv) Acceleration test *E: Cheetah LMT system* *OM*: Time (s) to cover 1 m, 2 m, 3 m, 4 m (v) Hitting *OM:* Maximum velocity after 2 m multiplied by mass of body and WC	Strongest effects of trunk impairment were found during impulse of a hit, tilt test and time to cover first 2 m in acceleration test Impact of arm impairment was found to be larger than trunk impairment for turn test, 10-m sprint and time to cover 4 m in acceleration test Significant difference in 10-m sprint times, turn test and time to complete 1 m and 2 m in acceleration test between TIC scores 0 and 1.5 (*p* < 0.05). No significance in turn test between TIC scores for athletes without severe arm impairment Significant difference in both left and right tilt test between TIC scores 0 and TIC score 0.5, 1.0 and 1.5 (*p* < 0.05) Significant difference in sprint momentum between TIC scores 0 and TIC score 1.0 and 1.5 (*p* < 0.05)
Altmann et al. [31]	WC basketball and WC rugby players with ≥ 1 year experience	*N* = 27 (M, 37.4 ± 10.2 y)	(iv) WC tilt *E:* Tape measure attached to pulley system *OM:* Difference between maximum and initial height (mm) (v) Acceleration *E*: Cheetah LMT attached to WC *OM*: Time to push 1 m (s) (vi) Sprint momentum *OM:* Average velocity of first 2 m multiplied by body mass (kg m s^−1^)	*Lateral isometric force and WC tilt* Significant moderate correlation between left and right mean isometric force and left and right mean tilt height (*r* = 0.50, *p* = 0.007) Significant difference in tilt height between cluster 1, 2 and 3 *Forward isometric force and acceleration test* Significant moderate correlation (*r* = 0.59, *p* = 0.001) Significant difference between clusters 1 and 4 *Forward isometric force and sprint momentum* Strong significant correlation (*r* = 0.79, *p* = 0.0001) Significant difference between clusters 1 and 4, 1 and 3, 2 and 3
Daniel et al. [63]	CP football players	*N* = 35 (M; 24.8 ± 6.3 y) FT5: *n* = 5 (21.6 ± 2.8 y) FT6: *n* = 8 (24.5 ± 4.0 y) FT7: *n* = 19 (25.2 ± 7.3 y) FT8: *n* = 3 (28.0 ± 11.1 y)	(i) Ball dribbling in a straight line *E:* Stopwatch *OM:* Time (s) (ii) Ball dribbling with short COD *E:* Stopwatch *OM:* Time (s) (iii) Ball dribbling with long COD *E:* Stopwatch *OM:* Time (s) (iv) Ball dribbling in a square *E:* Stopwatch *OM:* Time (s)	*Test reliability and reproducibility* All the tests showed good-to-excellent reliability (ICC = 0.86–0.97) *Construct validity* Strong correlations found across tests between sessions (*r* = 0.88–0.97; *p* < 0.001) Strong correlations found across tests within sessions (Session 1: *r* = 0.85–0.94, *p* = < 0.001; Session 2: *r* = 0.72–0.93, *p* < 0.001) *Between-group differences (Session 2 only)* Pair comparisons showed a significant difference between FT6 and FT8 players for ball dribbling with short COD Large effect sizes found for FT8 players when compared with other sport classes for all tests (d_g_ = 0.82–1.75)
Fortin-Guichard et al. [56]	International-level VI swimmers who compete in 100-m freestyle	*N* = 45 (49.8% F; 20.8 ± 6.8 y)	(i) Mean lateral position in lane (ability to swim in straight line) *E:* Video camera *OM:* Average absolute distance from centre of the lane (cm)	Significant correlation found between mean lateral position in lane and VA (*r* = 0.71,* p* < 0.001), contrast sensitivity (*r* = − 0.60, *p* < 0.001) when excluding missing values Significant correlation found between mean lateral position in lane and VA(*r* = 0.71, *p* < 0.001), contrast sensitivity (*r* = − 0.54,* p* < 0.001), light sensitivity (*r* = 0.41, *p* < 0.01), depth perception (*r* = 0.47, *p* < 0.01) and visual search (*r* = 0.53, *p* < 0.001) when including dummy values VA alone is not sufficient to predict mean lateral position, as depth position contributes to the quality of the prediction, even though a decrease in correct classification was observed No significant correlation between visual function measures and mean lateral position for participants with VA ≤ 3.5 logMAR Strong indication that light perception might influence how well participants with VA > 3.5 logMAR maintain their lateral position in lane (*p* = 0.06)
Henríquez et al. [64]	(i) CP football players from national Spanish team (ii) CG of non-disabled football players	*N* = 67 (36 M, 31F) CP footballers: *n* = 28 (18 M, 27.4 ± 7.2 y; 10 F, 27.8 ± 12.2 y) CG: *n* = 39 (18 M, 26.7 ± 6.0 y; 21 F, 21.2 ± 4.2 y)	(i) 10-m sprint *E:* Timing gates *OM:* Time (s) (ii) 505 agility test *E:* Timing gates *OM:* Time (s)	Players from all groups displayed significant differences between their non-dominant and dominant legs in COD outcomes and deficits (*p* < 0.05, *d*_g_ = − 0.40 to − 1.46). These asymmetries did not show a significant difference between sex with or without impairment Male individuals had faster sprint times than females in both CP (*p* < 0.01, *d*_g_ = − 2.26, large) and CG (*p* < 0.05, *d*_g_ = 2.28, large) Male individuals with CP demonstrated faster directional COD speed and slower COD deficit than female individuals with CP (*p* < 0.01, *d*_g_ = − 1.68 to − 2.53, large) Significant correlation between 10-m sprint speed and dominant 505 COD for both male and female CP groups and female CG (*p* < 0.01)
Hogarth et al. [65]	Competitive swimmers who had an eligible physical impairment or were without a physical impairment	*N* = 80 (50 M, 30 F) Para swimmers: *n* = 70 *Limb deficiency*: *n* = 29 (15 M, 20.3 ± 4.2 y; 14 F, 21.3 ± 5.2 y) *Hypertonia*:* n* = 24 (20 M, 25.3 ± 6.4 y; 4 F, 21.5 ± 7.5 y) *IMP*: *n* = 17 (9 M, 34.8 ± 5.3 y; 8 F, 30.6 ± 11.7 y) Able bodied: *n* = 10 (6 M, 21.5 ± 4.5 y; 4 F, 19.3 ± 2.4 y)	**Activity limitation** Tethered swim *E:* Load cell *OM:* Maximum tether force in Newtons, average tether force in Newtons, fatigue index **Performance** 10-m maximal freestyle swim *E:* Video camera *OM:* Swim speed m.s^−1^, stroke rate min^−1^, stroke length (m)	Able-bodied swimmers had higher absolute and normalised tether forces than Para swimmers Moderate positive correlations found between absolute and normalised maximum tether forces and sport class (Para swimmers) [*τ* = 0.55, *p* < 0.001] Non-linear relationship between maximal swim speed and tether force in participant cohort (adjusted *R*^2^ = 0.78–0.80, *p* < 0.001) Para swimmers with limb deficiency showed a stronger relationship between tether force and maximum swim speed (adjusted R^2^ = 0.78–0.82, *p* < 0.001) than did swimmers with hypertonia (adjusted *R*^2^ = 0.54–0.73, *p* < 0.001) and IMP (adjusted *R*^2^ = 0.61–0.70, *p* < 0.001)
Hogarth et al. [66]	Para swimmers with an eligible physical impairment	*N* = 132 (81 M, 51 F) *HAA* (16 M, 10 F; 23.6 ± 13.9 y) *IMP* (16 M, 10 F; 24.9 ± 14 y) *IPROM* (5 M, 7 F; 21.8 ± 5.9 y) *Limb deficiency* (40 M, 17 F; 21.1 ± 6.7 y) *Short stature* (4 M, 7 F; 18.5 ± 4.4 y)	**Activity limitation** Passive drag *E:* Load cell, towing rig *OM:* Normalised passive drag (N kg^−1^) **Performance** 100-m race times obtained from public website OM: Swim speed (m s^−1^)	Strong negative correlation (*ρ* = − 0.77, *p* < 0.001) between normalised passive drag force and 100-m race speed for combined cohort 62% of variance in 100-m freestyle performance in combined cohort explained normalised passive drag force Physical impairment type was found to affect the relationship between passive drag and 100-m freestyle race speed when included in linear regression (*R*^2^ = 0.65, *χ*^2^ = 11.5, *p* = 0.025)
Nogueira et al. [67]	Elite CP footballers	*N* = 60 (sex NR, 26.2 ± 1.66 y)	(i) Side stepping *E:* NR *OM:* Time (s) to complete 25 cycles (ii) Split jumps *E:* NR *OM:* Time (s) to complete 25 cycles	No significant differences found between classification classes and both tests (split jump: *p* = 0.155, side stepping: *p* = 0.351) Excellent internal consistency found for both tests (side stepping: ICC 0.82–0.93; split jumps: ICC 0.83–0.94) Tests showed good reproducibility (side stepping: ICC 0.66; split jumps: ICC 0.83) Both tests produced excellent objectivity (similar results found between evaluators)
Peña-González et al. [68]	International Para footballers from 23 national teams	*N* = 180 (sex NR, 26.1 ± 6.3 y)	(i) Modified agility test *E:* Photocell system *OM:* Time (s) (ii) Dribbling speed test *E:* Photocell system *OM:* Time (s)	*Within-session reliability* – Test reliability was excellent for modified agility test (ICC_2-1_ = 0.91, SEM = 5.75%) and dribbling speed test (ICC_2-1_ = 0.92, SEM = 4.66%) for entire CP group *Criterion-related validity* – Correlations between modified agility test and dribbling speed test for overall group and sport classes high to very high (*r* = 0.60–0.80, *p* < 0.01) Regression analysis indicated that 18.2% of sport class variance could be explained by the two tests – Significant differences were found between both tests between FT1 and FT2, FT1 and FT3 (*p* < 0.01)
Reina et al. [69]	(i) International Para footballers (ii) Football players with national competition experience	*N* = 136 (Sex NR, 23.7 ± 6.6 y) Para footballers: *n* = 99 FT5: *n* = 8 (23.2 ± 6.4 y) FT6: *n* = 12 (27.1 ± 8.9 y) FT7: *n* = 51 (24.8 ± 6.2 y) FT8: *n* = 14 (26.5 ± 7.6 y) CG: *n* = 37 (19.6 ± 3.4 y)	(i) Illinois agility test *E:* Electronic timing system *OM:* Time (s) (ii) Modified agility test *E:* Electronic timing system *OM:* Time (s)	A significant positive correlation found between the Illinois and modified agility test (*r* = 0.74, *p* < 0.001) Significant correlations found between both tests and classes FT6 (*r* = 0.84, *p* < 0.01), FT7 (*r* = 0.52, *p* < 0.001), FT8 (*r* = 0.641, *p* < 0.01) CG completed both the tests faster than Para footballers No significant difference found between Para football sport classes and Illinois agility test Significant differences found between FT8 and other Para football sporting classes for the modified agility test: FT5 (*p* = 0.005; *d* = 1.32, large), FT6 (*p* = 0.034; *d* = 1.01, large), FT7 (*p* = 0.026; *d* = 1.04, large) *Intra-session reliability* ICCs ranged from 0.76 to 0.96 for both Para footballers and CG for both tests
Reina et al. [70]	(i) International Para footballers classified according to the IFCPF classification rules (ii) CG without an impairment or injury	*N* = 113 (sex NR) Para footballers: *n* = 82 (25.2 ± 6.8 y) CG: *n* = 31 (19.5 ± 3.3 y)	(i) Illinois agility test *E:* Electronic timing system *OM:* Time to complete (ii) Stop and go test *E:* Sensing mats, electronic timing system *OM:* Time to complete (iii) 40-m sprint *E:* Infrared photocells *OM:* Time (s)	Significant correlations found between all tests for Para footballers except between: Illinois agility test with ball and stop and go test without ball and stop and go test without ball and 10-m sprint time Significant differences and large effect sizes found when Para footballers performed tests with and without dribbling the ball (0.53 < *ηp*2 < 0.97; *p* < 0.05) Higher coefficient of variation found for FT5, FT6 and FT7 players in trials requiring players to dribble ball Significant and large practical differences were found between groups for all tests (*ηp*2 = 0.35–0.62, large; *p* < 0.01)
Reina et al. [71]	(i) International Para footballers classified according to IFCPF classification rules (ii) CG without an impairment or injury	*N* = 171 (sex NR) Para footballers: *n* = 132 (25.8 ± 6.7 y) CG: *n* = 39 (19.4 ± 3.3 y)	(i) Vertical jump (CMJ) *E:* Force platform *OM:* Height jumped (m) (ii) Horizontal jumps: SBJ, four bounds for distance, triple hop for distance *E:* Tape measure *OM:* Distance from start line to landing heel. Normalised to standing height	*Intra-session reliability* Good-to-excellent relative intrasession reliability scores found for all measures for both groups (ICC: 0.78–0.97, SEM < 10.5%) *Criterion-related validity* Large significant correlations found between CMJ and horizontal jumps for Para footballers (0.59 < *r* < 0.73, *p* < 0.01) *Between-group differences* CG performed longer/higher jumps compared to Para footballers. Significant differences and moderate-to-large effect sizes found (0.85 < ES < 5.54, *p* < 0.01)
Reina et al. [72]	International-level Para footballers from 5 national teams	*N* = 48 (M, 23 ± 7 y)	**Activity limitation** (i) One leg stance, tandem walk *E:* Stopwatch *OM:* Time (s) (ii) Split jumps, side stepping *E:* Contact mat *OM:* Time (s) to complete 25 cycles (iii) SBJ, triple hop, four bounds for distance *E:* Tape measure *OM:* Distance (m) from start line to heel strike. Normalised to standing height (iv) CMJ *E:* Leg stiffness device *OM:* Height (cm) (v) 20 m sprint, modified and 505 agility test *E:* Timing gates *OM:* Time (s) **Performance** Match load from GPS *OM:* Max velocity (km h^−1^), distance covered (m min^−1^), number of accelerations/decelerations (number min^−1^), player load (AU min^−1^), peak metabolic power (watt min^−1^)	*Activity limitation* Significant difference between sporting classes and performance in static balance, split jumps, side stepping, SBJ, triple hop, four bounds, CMJ, modified agility test, sprint times at 10 m, 15 m and 20 m (*p* < 0.05) *Performance* Significant differences were found between classes for maximum velocity, high acceleration and moderate deceleration FT8 class covered significantly more distance than FT5/6 class (*p* < 0.05) *Activity limitation and performance relationship* Low to moderate for all players
Reina et al. [45]	CP footballers from 25 national teams	*N* = 259 (sex NR, 25.46 ± 6.15 y)	**Activity limitation** (i) Split jumps *E:* Contact mat *OM:* Time (s) to complete 25 repetitions (ii) Side stepping *E:* Contact mat *OM:* Time (s) to complete 25 repetitions **Performance** Match load *E:* GPS units *OM:* Mean/maximum velocity (km h^−1^), total distance covered (m min^−1^), distance covered at different intensities (m min^−1^), accelerations, decelerations (number min^−1^)	*Activity limitation measures* FT1 players recorded the slowest performances and FT3 players the fastest *Activity limitation and match performance* Significant correlation between side stepping, distance covered at low intensity running (*r* = − 0.14, *p* = 0.028), moderate accelerations (*r* = 0.21, *p* < 0.001) and high accelerations (*r* = − 0.20, *p* < 0.001) Significant correlation between split jumps, moderate (*r* = − 0.16, *p* = 0.005) and high accelerations (*r* = − 0.18, *p* = 0.006) *Intra-session reliability* ICCs ranged from 0.83 to 0.87 for activity limitation tests indicating good-to-excellent reliability
Roldan et al. [73]	International Para footballers	*N* = 69 (sex NR, 25.4 ± 7.6 y)	(i) Tandem walk *E:* Stopwatch *OM:* Time (s) to complete 5 m (ii) Rapid heel-toe contacts *E:* Tapping platform (35 × 20-cm) *OM:* Time (s) to complete 25 cycles (iii) CMJ *E:* NR *OM:* Height (m) (iv) Standing broad jump, triple hop for distance *E:* Tape measure *OM:* Distance (m) (v) 20-m sprint, modified agility test, 505 agility test with ball *E:* Photocell system *OM:* Time (s)	*Within-session reliability* No significant differences found between the two trials performed for each activity limitation test (*p* > 0.05, *d* = 0.00 to 0.35) Excellent reliability found for triple hop and sprint time between 10 and 20 m (ICC_2,1_ = 0.91–0.93; SEM = 0.11–0.31) Low reliability scores found for sprint time between 5 and 10 m (ICC_2,1_ = 0.44, SEM = 0.19)
Sarabia et al. [74]	International CP football players	*N* = 21 (M; 25.5 ± 6.2 y) Bilateral spasticity or diplegia: *n* = 4 (28.6 ± 8.1 y) Ataxia or athetosis: *n* = 6 (24.2 ± 7.2 y) Unilateral spasticity or hemiplegia: *n* = 11 (25.2 ± 5.2 y)	(i) Side-step *E:* Tape measure *OM:* Distance (m) (ii) Rapid heel-toe placement *E:* Stopwatch, contact mat *OM:* Time (s) (iii) Split jumps, running in place *E:* Contact mat *OM:* Time (s) to complete 25 cycles (iv) Side stepping *E:* Contact mat *OM:* Time (s) to complete 15 cycles (v) Tandem walk *E:* Stopwatch *OM:* Time (s) to complete 10 steps and 5-m (vi) One-leg stance *E:* Stopwatch *OM:* Time (s) (vii) CMJ *E:* Optojump *OM:* Height (cm) (viii) SBJ, triple hop for distance, four bounds for distance *E:* Tape measure *OM:* Distance (m). Normalised to standing height (ix) Modified agility test, stop and go test, 40-m sprint *E:* Timing gates *OM:* Time (s) (x) Hexagon agility test *E:* Stopwatch *OM:* Time (s) to complete 3 revolutions (xi) 10-m speed skip *OM:* Timing gates *E:* Time (s) between 10 and 20 m	Only five tests included in decision trees: side step, triple hop, side-stepping, tandem walk and speed skip *Overall* Decision tree: 8 decision nodes, 24 branches and 17 leaves. Included side step, triple hop for distance and side stepping tests (precision = 67.0%) Moderate-to-high accuracy (MCC = 0.516, AUC-ROC = 0.825) *Spastic diplegia* Decision tree: 2 decision nodes, 6 branches and 5 leaves. Included side step and tandem walk tests (precision = 89.1%) Moderate-to-high accuracy (MCC = 0.715, AUC-ROC = 0.874) *Ataxia/athetosis* Decision tree: 5 decision nodes, 15 branches and 11 leaves. Included side step, speed skip, triple hop for distance and side-stepping tests (precision = 86.5%) Moderate-to-high accuracy (MCC = 0.693, AUC-ROC = 0.875) *Spastic hemiplegia* Decision tree: 2 decision nodes, 6 branches and 5 leaves. Included side step and side stepping tests (precision = 90.9%) Moderate-to-high accuracy (MCC = 0.766, AUC-ROC = 0.980)
Van Biesen et al. [75]	(i) TTP with ID (ii) TTP without ID	*N* = 88 TTP with ID: *n* = 71 (41 M, 27 ± 8 y; 30 F, 28 ± 8 y) TTP without ID: *n* = 17 (12 M, 24 ± 12 y; 5 F, 20 ± 10 y)	Standardised tactical proficiency test focused on service return *E:* Video camera *OM:* Expressed in points (maximum score 40)	No significant correlation between IQ and total score for TTP with ID Tactical proficiency scores for all service variations were significantly lower in TTP with ID compared with TTP without ID (*p* < 0.001)
Van Biesen et al. [76]	(i) Elite middle-distance and long-distance runners with mild II (ii) Runners without impairment (CG)	*N* = 67 Runners with II: *n* = 34 (22 M, 12 F; 24.4 ± 4.5 y) CG: *n* = 33 (27 M, 6 F; 31.4 ± 11.2 y)	Running pacing test (400 m) *E:* Cones, whistle *OM:* Split times (s)	Significant main effect between groups, with II runners found to deviate more from target time than CG (*p* < 0.001) A significant trial-group interaction effect (*p* < 0.05) revealed that the ability to self-regulate pace during the final 200 m improved for CG (Trial 1: 1.7 ± 1.0 s, Trial 2: 0.9 ± 0.8 s), whereas II runners deviated more in trial 2 (4.4 ± 4.3 s) than trial 1 (3.2 ± 3.9 s)
Yanci et al. [77]	(i) CP football players with an eligible impairment (ii) CG with no impairment	*N* = 123 (sex NR) Para footballers: *n* = 87 (25.1 ± 6.75 y) CG: *n* = 36 (19.51 ± 3.40 y)	(i) Stop and go test *E:* Sensing mats, photocells *OM:* Time (s) (ii) Turning and dribbling test, Illinois agility test *E:* Photocells *OM:* Time (s)	All tests performed significantly better by CG compared with players with bilateral spasticity, athetosis/ataxia, and unilateral spasticity (*p* < 0.01) Players with unilateral spasticity and minimum impairment performed significantly better on all tests when compared with the group of players with bilateral spasticity and athetosis/ataxia (*p* < 0.01) No significant difference found between test performance and competition history for CP football players No significant relationship found between test performance, years of football experience, weekly strength/football training sessions for CP footballers (*p* = 0.12–0.95)

*AUC-ROC* area under the receiver operating characteristic curve, *CG* control group, *CP* cerebral palsy, *CMJ* countermovement jump, *COD* change of direction, *F* female, *HAA* hypertonia athetosis ataxia, *ID* intellectual disability, *II* intellectual impairment, *IMP* impaired muscle power, *IPROM* impaired passive range of motion,* M* male, *MCC* Matthews’ Correlation Coefficient, *NR* not reported, *s* seconds, *SBJ* standing broad jump, *TIC* Trunk Impairment Classification, *TTP* table tennis player, *VA* visual acuity, *VI* vision impairment, *WC* wheelchair,* y* years

#### CP Football (Physical)

The studies focusing on CP football have assessed activity limitations through a variety of balance, coordination, horizontal/vertical jumps, change of direction and acceleration/sprint tests. It should be noted that the classification classes for CP football have recently been upgraded from FT5–FT8 (FT5: diplegia, asymmetric diplegia, double hemiplegic or dystonic, FT6: athetosis, dystonic, ataxic, or mixed cerebral palsy or related neurological conditions, FT7: hemiplegic, FT8: minimal impairment criteria) [79] to FT1–FT3 where athletes are allocated to a class based on their activity limitation (1 = severe involvement, 2 = moderate involvement, 3 = minimal involvement) and eligible impairment (A = bilateral spasticity, B = athetosis/dystonia [dyskinesia] or ataxia, C = unilateral spasticity) [13].

Static balance was assessed through one leg stance on both dominant and non-dominant leg [72, 74], while dynamic balance was assessed through a 5-m tandem walk test [72–74] and side-step test [74]. Significant differences were found between sporting classes for static balance while no significant differences were found between sporting classes in dynamic balance [72]. In relation to match play performance, FT8 players who displayed better results in dynamic balance were found to perform more moderate accelerations [72].

The coordination measures used to assess activity limitation were split jumps [45, 67, 72, 74], side stepping [45, 67, 72, 74], hexagon agility test [74] and running in place [74]. Despite FT1 players recording the slowest and FT3 players the fastest time for both split-jump and side-stepping measures [45, 67], no significant differences were found between classification classes. However, an earlier study that employed the old classification system found significant differences between sporting classes for both split-jump and side-stepping measures [72]. Significant associations were found between split-jump and side-stepping measures and moderate/high accelerations determined from a GPS unit during match play [45]. Split-jump and side-stepping tasks displayed good levels of intra-session reliability, with ICC values of 0.87 and 0.83 respectively [45]. However, the test–retest reliability of side stepping was only found to be moderate (ICC: 0.66), while split jumps demonstrated a good level of test–retest reliability (ICC: 0.83) [67].

The jump tests used to assess activity limitation were vertical jump [71–73], standing broad jump [71–73], four bounds for distance [72, 73] and triple hop for distance [71–74]. In the study examining all four jump measures, good-to-excellent intra-session reliability scores were found for both CP footballers and the control group [71]. Test–retest reliability was also found to be high for vertical jump in CP footballers (ICC: 0.89) [73]. The association between jump measures and performance was evaluated in a single study, with significant differences found between sporting classes for all jump measures [72].

Finally, the change of direction and speed measures used were the modified agility test [68, 69, 72–74], Illinois agility test [69, 70, 77], 505 agility test [64, 72, 73], dribbling speed test [68], turning and dribbling test [77], stop and go test [70, 74, 77], ball dribbling in various patterns [63] and sprints over varying distances [64, 70, 72–74]. Regarding the modified agility test, three studies reported a significant difference between classes [68, 69, 72]. The modified agility test also showed high within-session reliability for the overall CP football group (ICC: 0.82) [69, 73], (ICC: 0.91) [68] and the control group (ICC: 0.76) [69]. Good-to-excellent reliability was also found when players were broken up into their respective sport classes, (ICC: FT1 = 0.94, FT2 = 0.87 and FT3 = 0.84) [68]. The Illinois agility test displayed excellent within-session reliability for the overall CP group (ICC: 0.96) [69] as well as in another group of CP footballers with (ICC: 0.95) and without (ICC: 0.84) the ball [70]. Significant differences and large effect sizes were found when CP footballers and a control group performed the Illinois agility test with and without the ball, with statistically significant and large practical differences found between CP football groups (FT5–FT8) [70]. Similarly, significant differences were found between players with no CP, bilateral spasticity, athetosis/ataxia and unilateral spasticity when performing the Illinois agility test with a ball [77]. In the study by Daniel et al. [63], good-to-excellent test–retest reliability was reported for all ball dribbling tests (ICC: 0.86–0.97). The dribbling speed test showed excellent within-session reliability for the overall group (ICC: 0.92) and good-to-excellent reliability for sport classes (ICC: FT1 = 0.95, FT2 = 0.84, FT3 = 0.88) [68]. Significant differences in dribbling test performance were found between FT1 and FT2 and FT3 classes [68]. Similarly, in other tests requiring players to dribble a ball (i.e. stop and go test, turning and dribbling test), significant differences were found between players with no CP and bilateral spasticity, athetosis/ataxia and unilateral spasticity [77]. When considering linear sprint ability, significant differences were found during a 40-m sprint with and without the ball between FT5 and FT8, FT6 and FT8, FT7 and FT8 classes [70]. A 20-m sprint test without the ball also found significant differences between classes [72].

#### Wheelchair Rugby (Physical)

In wheelchair rugby players, activity limitation was also assessed through a series of sprint, acceleration and change of direction tests [23, 31]. Significant differences were found between TIC scores 0 and 1.5 for 10-m sprint times, turn test and time to complete 2 m in acceleration test [23]. However, the explained variance by arm impairment for a 10-m sprint time and turn test was larger than trunk impairment [23].

#### Para Swimming (Physical and Vision)

The studies examining activity limitation in Para swimming for athletes with a physical impairment used a load cell to quantify propulsive forces [65] and passive drag [66]. Both studies examined association with performance with one study examining maximal freestyle swim speed through a 10-m calibrated zone [65], while the other obtained the participants’ best 100-m freestyle swim times from a public website [66]. A linear regression found physical impairment types affected the relationship between passive drag and 100-m freestyle swim speed [66]. Physical impairment types were also found to influence the relationship between tether force and maximal freestyle swim speed as Para swimmers with limb deficiency displayed stronger relationships than Para swimmers with hypertonia and impaired muscle power [65].

For Para swimmers with vision impairments, activity limitation was assessed by evaluating their ability to swim in a straight line, as determined from their mean lateral position in the lane from video footage [56]. Visual acuity along with depth perception was found to help in predicting high and low performing swimmers based on their mean lateral position in the lane [56]. However, despite this, the percentage of correct classifications decreased slightly from 89.5 to 86.9% [56].

#### Athletics (Intellectual)

A novel 400-m pacing running test was used to explore potential differences between athletes with intellectual impairment and those without [76]. The results indicated that athletes with intellectual impairment significantly deviated from the target time compared to non-impaired runners, irrespective of the presence of feedback [76].

#### Table Tennis (Intellectual)

A semi-standardised test assessing tactical proficiency through service returns has been used to determine differences between athletes with and without an intellectual disability [75]. Tactical proficiency scores for all service returns were significantly lower in table tennis players with an intellectual disability [75].

### Quality Assessment

Quality assessment of the included studies is displayed in Table 7. Overall ‘yes’ scores ranged from 62.5% to 87.5% indicating a low-to-moderate bias. Half the studies did not provide a specific inclusion criterion (Q1). The participants and settings (Q2) were generally well described. However, 12 studies failed to report sex. Although we can assume that the evaluation of individuals’ disability was valid and reliable, Q3 was deemed unclear because the studies did not specify how the disability was diagnosed. However, they all provided a distinct diagnosis of the health condition and/or breakdown of sporting classes (Q4). The studies effectively identified and addressed confounding factors such as level of impairment (Q5 and Q6). All studies used valid and reliable outcome measures, as well as appropriate statistical analysis (Q7 and Q8).

**Table 7:** Risk of bias assessed by the Joanna Briggs Institute critical appraisal tool for included studies

Study	Q1	Q2	Q3	Q4	Q5	Q6	Q7	Q8	Total (% score yes)	Risk of bias
Allen et al. [55]	N	Y	U	Y	Y	Y	Y	Y	75	Low
Altmann et al. [30]	Y	N	U	Y	Y	Y	Y	Y	75	Low
Altmann et al. [23]	Y	N	U	Y	Y	Y	Y	Y	75	Low
Altmann et al. [31]	Y	Y	U	Y	Y	Y	Y	Y	87.5	Low
Altmann et al. [42]	N	Y	U	Y	Y	N	Y	Y	62.5	Moderate
Beckman et al. [32]	N	Y	U	Y	Y	Y	Y	Y	75	Low
Bjerkefors et al. [47]	Y	Y	U	Y	Y	Y	Y	Y	87.5	Low
Connick et al. [43]	N	Y	U	Y	Y	N	Y	Y	62.5	Moderate
Connick et al. [33]	Y	Y	U	Y	Y	Y	Y	Y	87.5	Low
Daniel et al. [63]	Y	Y	U	Y	Y	Y	Y	Y	87.5	Low
Domínquez-Díez et al. [34]	N	Y	U	Y	Y	Y	Y	Y	75	Low
Fortin_Guarchard [56]	Y	Y	U	Y	Y	Y	Y	Y	87.5	Low
Henríquez et al. [64]	Y	Y	U	Y	Y	N	Y	Y	75	Low
Hogarth et al. [18]	Y	Y	U	Y	Y	U	Y	Y	75	Low
Hogarth et al. [19]	N	Y	U	Y	Y	Y	Y	Y	75	Low
Hogarth et al. [65]	Y	Y	U	Y	Y	Y	Y	Y	87.5	Low
Hogarth et al. [66]	Y	Y	U	Y	Y	Y	Y	Y	87.5	Low
Hyde et al. [35]	Y	Y	U	Y	N	N	Y	Y	62.5	Moderate
Krabben et al. [57]	N	Y	U	Y	Y	Y	Y	Y	75	Low
Krabben et al. [58]	N	N	U	Y	Y	Y	Y	Y	62.5	Moderate
Latham et al. [59]	N	Y	U	Y	Y	Y	Y	Y	75	Low
Liljedahl et al. [36]	Y	Y	U	Y	Y	N	Y	Y	75	Low
Liu et al. [37]	N	Y	U	Y	Y	Y	Y	Y	75	Low
Maia et al. [44]	Y	Y	U	Y	Y	U	Y	Y	75	Low
Mason et al. [38]	N	Y	U	Y	Y	N	Y	Y	62.5	Moderate
Mason et al. [39]	N	Y	U	Y	Y	N	Y	Y	62.5	Moderate
Myint et al. [60]	N	N	U	Y	Y	Y	Y	Y	62.5	Moderate
Nicholson et al. [48]	N	Y	U	Y	Y	U	Y	Y	62.5	Moderate
Noguiera et al. [67]	N	N	U	Y	Y	Y	Y	Y	62.5	Moderate
Peña-González et al. [68]	N	N	U	Y	Y	Y	Y	Y	62.5	Moderate
Pineda et al. [49]	Y	Y	U	Y	N	N	Y	Y	62.5	Moderate
Reina et al. [69]	N	N	U	Y	Y	Y	Y	Y	62.5	Moderate
Reina et al. [70]	N	N	U	Y	Y	Y	Y	Y	62.5	Moderate
Reina et al. [71]	N	N	U	Y	Y	Y	Y	Y	62.5	Moderate
Reina et al. [72]	Y	Y	U	Y	Y	Y	Y	Y	87.5	Low
Reina et al. [45]	Y	N	U	Y	Y	Y	Y	Y	75	Low
Roldan et al. [46]	Y	Y	U	Y	Y	Y	Y	Y	87.5	Low
Roldan et al. [73]	Y	N	U	Y	Y	Y	Y	Y	75	Low
Rosso et al. [40]	N	Y	U	Y	Y	Y	Y	Y	75	Low
Sarabia et al. [74]	N	Y	U	Y	Y	Y	Y	Y	75	Low
Stalin et al. [61]	N	Y	U	Y	Y	Y	Y	Y	75	Low
Stalin et al. [62]	N	Y	U	Y	Y	Y	Y	Y	75	Low
Van Biesen et al. [50]	Y	Y	U	Y	Y	Y	Y	Y	87.5	Low
Van Biesen et al. [51]	N	Y	U	Y	Y	Y	Y	Y	75	Low
Van Biesen et al. [52]	Y	Y	U	Y	Y	Y	Y	Y	87.5	Low
Van Biesen et al. [53]	Y	Y	U	Y	Y	Y	Y	Y	87.5	Low
Van Biesen et al. [54]	Y	Y	U	Y	Y	Y	Y	Y	87.5	Low
Van Biesen et al. [75]	N	Y	U	Y	Y	U	Y	Y	62.5	Moderate
Van Biesen et al. [76]	Y	Y	U	Y	N	N	Y	Y	62.5	Moderate
Vanlandewijck et al. [41]	N	Y	U	Y	Y	Y	Y	Y	75	Low
Yanci et al. [77]	Y	N	U	Y	Y	Y	Y	Y	75	Low

Q1. Were the criteria for inclusion in the sample clearly defined? Q2. Were the study subjects and the setting described in detail? Q3. Was the exposure measured in a valid and reliable way? Q4. Were objective standard criteria used for measurement of the condition? Q5. Were confounding factors identified? Q6. Were strategies to deal with confounding factors stated? Q7. Were the outcomes measured in a valid and reliable way? Q8. Was appropriate statistical analysis used?

*N* no, *Q* question, *U* unclear, *Y* yes

## Discussion

Classification is a critical aspect in Para sports, requiring a robust and valid system that ensures fairer competition in determining who is eligible to compete. This review focused on impairment and activity limitation measures used to assess eligible impairments in Para sport athletes for the purpose of classification research, with the goal of identifying potential measures for evidence-based classification. Our findings highlight the heterogeneity of measures so far evaluated, which may be attributed to the eligibility of specific impairments for participation in certain Para sports or the challenge of identifying/developing measures with the necessary measurement properties to assess eligible impairments. Despite this, isometric strength and tapping tasks were the most reported measures used across sports to evaluate strength and coordination impairments respectively. The complexity of evaluating cognitive aspects is evident in the variety of measures identified for intellectual impairments, while research on vision impairments has acknowledged the importance of assessing additional measures of visual function.

### Strength Impairment Measures

The assessment of strength in current classification practices relies heavily on manual muscle testing [7–12, 15, 16, 80]. However, recent literature has found isometric strength to be the most valid method for inferring loss of muscle strength in classification procedures [20]. This is because isometric strength measures are mostly resistant to the effects of training [20, 81] and have been found to be the best measure of voluntarily maximal contractile force compared to isotonic and isokinetic measures [20]. To maintain the validity of isometric measures in classification, it is important to evaluate their relevance to sport performance with factors such as selection and the number and position of joints taken into consideration [20].

Of the studies assessing isometric strength, one assessed the use of single-joint measures on swimming performance with low-to-moderate correlations found [18]. However, given isometric strength was only assessed at the shoulder and hip, it is evident that this study failed to account for all the joints relevant to swimming performance. For example, these assessments do not describe strength impairments associated with knee flexion and ankle plantarflexion, which are important for starts, turn performance or generating propulsion during kicking. In addition, previous research evaluating strength measures using single joints has found little relationship with functionality [82], which may offer a valid explanation as to why low-to-moderate correlations were found. To account for greater variance in performance, isometric strength measures incorporating the use of multiple joints may be more effective as stronger relationships with athletic performance have been established [83].

Research that evaluated isometric strength using a combination of single-joint and multi-joint measures primarily found significant moderate-to-strong correlations with sprint performance in wheelchair rugby [39] and wheelchair track athletes [33]. However, the combination of arm extension and trunk flexion as a measure of isometric strength in wheelchair track racers was found to be significantly stronger with top speed during wheelchair propulsion than isolated trunk strength alone [33]. Given that testing batteries used for classification procedures should be parsimonious multi-joint measures should be used wherever possible [5]. This will minimise the number of assessments needed to be performed and reduce the effect of fatigue, which may be a confounding factor in current classification procedures [84]. A comprehensive testing battery can still be achieved provided the joint contributions and positions assessed are specific to the sport. However, further research is needed to identify which joint contributions accurately demonstrate activity limitation in Para sports when assessing strength impairments using isometric load cell measures, as well as establish the reliability of these measures in Para sport athletes.

In addition to measures of isometric strength, grip strength, [33, 35, 84] static and dynamic trunk balance [30, 40] were also identified as methods for assessing strength impairments. Grip strength is an important aspect in sports that require grasping and the application of force [85], with significant associations found with performance measures in seated throwers [35] and wheelchair racers [33]. Dynamic trunk balance has been found to be effective in distinguishing between athletes with varying levels of trunk strength in wheelchair rugby [30] and sit skiing [40]. While dynamic trunk balance may play an important role in sport-specific tasks relevant to wheelchair sports (i.e. lunging in fencing and ball striking in tennis), the reliability of such measures for use in evidence-based classification is yet to be established.

### Coordination Impairment Measures

The current assessment of impairment types that affect motor coordination (i.e. hypertonia, ataxia, athetosis) in Para sports that include these impairments falls short of possessing the necessary measurement properties required for evidence-based classification. Therefore, researchers have explored the implementation of instrumented tapping tasks as a novel solution to overcome the shortcomings of the subjective evaluations currently utilised [17, 19, 43, 46, 86]. Tapping tasks are considered an effective impairment measure for use in evidence-based classification due to their ability to generate a ratio-scaled measure and evaluate muscle actions over multiple joints [17]. The main benefit of using multi-joint coordination measures is their close alignment with the muscle movements performed in the sport, increasing the likelihood of a strong association with activity limitations [5]. For example, to evaluate the primary muscle actions associated with running performance, the tapping task should mirror the cyclic nature of running. As such, reciprocal bilateral lower limb tapping would be deemed an appropriate evaluation method as it captures the combined impact of the muscle movements needed for successful performance in the activity of interest.

A variety of tapping tasks have been found to be valid and reliable in non-disabled populations [17, 19, 86]. However, research exploring the use of such tasks in Para sports is limited [19, 43, 45, 46]. While the assessment methods used in the studies reviewed were effective in distinguishing Para athletes from non-disabled participants, variations were observed in the relationship between these measures and athletic performance within Para sport athletes [19, 43, 45]. These conflicting results raise new questions such as whether the association between impairment and performance is constrained by factors such as the nature of the movement (discrete or continuous), the type of the movement (unilateral or bilateral), dominance of the limb, size of the target, severity of the impairment or the testing position. Further investigation is necessary to establish the effect of these factors on the use of tapping tasks as a means of measuring coordination impairments in various Para sports.

Despite tapping tasks being the most reported measure, other studies utilised accelerometers [44] and clinical measures (i.e. spiral, finger to nose and RMTs) [42] to evaluate coordination impairments. These evaluations offer a practical and accessible alternative to instrumented assessments that often necessitate specialised and costly equipment. Requiring only basic equipment such as a stopwatch, video camera, and pen and paper, these measures provide a practical and convenient solution for evaluating coordination impairments. This approach is likely to simplify logistics, lower costs and ease the burden on participating athletes.

### ROM Impairment Measures

Currently, many Para sports examine ROM impairments through passive ROM using a goniometer [7–12]. However, the studies reviewed focused on active ROM, which is likely a better indicator of activity limitations [43, 48]. Nevertheless, the lack of standardisation of ROM measures used in classification has made it difficult to determine the reliability of such methods. The time-consuming nature of measuring each joint and the limited information obtained from a single measurement [43] mean limited evidence exists to quantify the relationship with sport performance. Further research is needed to examine both passive and active ROM techniques and their association with performance.

One of the alternative approaches for assessing ROM impairments identified in this review was the use of a motion analysis system [35, 47]. However, the high cost, specialised laboratory requirements, limited capture volume and long post processing times associated with this technology [87] may make it an impractical solution for measuring ROM impairments. Furthermore, as classification procedures typically occur in the field away from laboratories, the feasibility of using motion capture for classification purposes may be limited. As a result, future research could focus on exploring the validity and reliability of portable devices such as inertial measurement units for examining ROM impairments.

### Intellectual Impairment Measures

Intelligence Quotient testing is a critical component of determining eligibility for athletes with intellectual impairments [22]. However, the resulting IQ score encompasses a wide range of cognitive abilities, with only a subset relevant to sports performance [6, 22]. Therefore, it is important to assess an athlete’s cognitive profile by measuring their sport intelligence through generic cognitive tests, rather than relying solely on a general measure of IQ.

Generic cognitive tests currently play a vital role in the classification process as they measure various cognitive abilities impacting sport performance and have been found to be effective in distinguishing between athletes with and without intellectual impairment [50–52]. However, these cognitive abilities present differently across sports, highlighting the need for further research to determine their relationship with sport performance. The identified low test–retest reliability for specific measures related to visual processing and executive function has prompted the exploration of additional measures, such as the Color Trails and MOT tests [53, 54]. Another alternative approach being explored for the assessment of intellectual impairment is the use of cognitive-motor dual tasking [49, 53]. This method could create a more realistic testing environment, reflecting the dynamic and complex nature of sports, where individuals perform multiple tasks simultaneously [22]. For example, a basketball player must dribble the ball while scanning the court for teammates and opponents, which requires the allocation of cognitive resources to both motor skills and game awareness. Although there is limited research on the use of cognitive-motor dual tasking in classification procedures, athletes with an intellectual impairment have demonstrated poorer performances in dual tasks compared with athletes without an impairment [49, 53]. Further research is necessary to establish its effectiveness as a means of measuring intellectual impairments in Para sport athletes for use in evidence-based classification.

### Vision Impairment Measures

Current classification procedures for assessing vision impairments primarily rely on the measurement of two types of visual function: visual acuity and visual field [6, 78, 88]. There has been a recent shift in Para sport research for vision impairments, driven by expert consensus, to adhere to evidence-based classification guidelines [4, 89–92]. Many sports that allow individuals with vision impairments to compete have emphasised the importance of considering additional aspects of visual function that may influence sports performance. These measures, including contrast sensitivity, light sensitivity, and depth and motion perception, have demonstrated varying relationships with performance across sports [56, 57, 61, 62].

Examining the relationship between impairment and performance in athletes with vision impairment requires consideration of various factors. Testing conditions play a significant role in enhancing the ecological validity of measures, particularly considering the significant impact of light on certain vision impairments [6]. For example, the assessment of vision impairment typically occurs indoors under standard light conditions; however, during skiing, athletes may encounter fog, reducing the relative contrast of visual information [62]. The subjective nature of the current measures highlights the importance of exploring more objective measures, as reliance on athletes’ honest responses makes the assessments susceptible to intentional misrepresentation [55, 88, 89]. Additionally, the relative age at which the impairment was acquired may serve as a mediating factor in the relationship between impairment and performance [6, 78]. Despite only one study examining differences between athletes with congenital and acquired vision loss and finding no significant differences [55], further consideration in classification procedures is needed.

Although only a small body of research exists, this section of the review is limited by its focus on studies corresponding to just one of the three research models outlined in the joint position stand [78]. Although additional studies aligning with the other two research models exist (i.e. simulation and component-analysis model in non-disabled athletes), they fall outside the scope of this review [93–97]. However, these studies are not without their limitations. For example, employing simulation techniques in fully sighted athletes might underestimate the relationship between impairment and performance, as they have developed skills essential to the sport without the influence of impairment [6]. Therefore, a combination of all three research models is required for the development of a comprehensive evidence-based classification system.

### Activity Limitation Measures

The measures used to evaluate activity limitations vary depending on the sport and impairments being assessed [5]. To develop standardised sport-specific measures that can evaluate how an athlete’s impairment impacts their performance, researchers must identify the activities that have the greatest influence on the athlete’s performance in that sport [5].

Jump measures have been recognised as a suitable test of activity limitation [5] and are often used to evaluate athletic performance in sports due to their ease, efficiency and associations with various performance measures (i.e. strength, linear speed and change of direction ability) [98]. Various jump measures have been evaluated for use in CP football but given these players exhibit differing levels of impairment (i.e. unilateral/bilateral spasticity, ataxia, dyskinesia, hemiplegia), horizontal jump measures (i.e. standing broad jump, four bounds for distance, triple hop for distance) could be more effective in distinguishing between impairment types [71]. These measures not only reflect strength and power during the jump, but also coordination and stability between segments during the landing phase to prevent forward movement. Additionally, they are closely related to other performance determinants of CP football such as a change of direction [99] and sprint ability [69].

Multiple studies have explored the use of change of direction and sprint tests as determinants of performance in CP football and their potential application in evidence-based protocols [64, 68–70, 72, 77]. These measures are valuable assessments of activity limitation due to their ability to distinguish between sporting classes and controls [68, 69, 72]. Similarly, various measures, such as acceleration, turn test and tilt test have been used to evaluate activity limitations in wheelchair rugby [23, 31]. While these measures appear promising for evaluating activity limitations in various Para sport athletes, additional measurements are required to make informed decisions due to the intricate nature of Para team sports.

Additionally, measures of activity limitation and their associations with performance during competition cannot be regarded as one-dimensional. This is because these activities vary depending on the role players assume in the team, making it difficult to draw inferences between the impact of impairment on performance during match play [100]. As such, the influence of factors such as a player’s position, team tactics and line-ups on performance remains unclear. It is also important to consider both the physical and technical variables associated with each sport to gain a better understanding of the impairment and performance relationship. Conducting scientific research during competition may be valuable for understanding the impact of impairment on performance as athletes are likely to exert maximum effort.

### Limitations and Future Research Directions

Although a comprehensive search was conducted across six online databases and manual searches of reference lists of included studies were performed, it is possible that relevant studies may have been missed by not searching grey literature. Nevertheless, we made a concerted effort to identify the most relevant databases and search terms related to the topic. Despite this, the review’s focus on studies specifically published for classification purposes may have led to the exclusion of other relevant studies that validate tests suitable for evidence-based classification but were conducted for other purposes. If not explicitly stated in the study, we differentiated between measures of impairment and activity limitation according to the definitions outlined in the IPC position stand [5]. However, we recognise that in some cases, measures of impairment may also serve as measures of activity limitation. Despite variations in study quality, all studies employed appropriate statistical analysis and assessed outcomes in a valid and reliable way. A meta-analysis was not feasible due to the heterogeneity of studies.

Future research should aim to reduce the dependence on non-disabled populations as the primary reference in classification procedures. Although normative performance ranges are crucial for the meaningful interpretation of future research involving athletes with impairments, the test–retest reliability of newly developed, physical impairment measures in Para athletes needs to be established. The limited sample sizes and evaluation of certain measures in single studies emphasise the necessity of examining larger Para sporting populations with differing levels of impairment to improve the credibility of evidence-based classification outcomes. The testing environment where these measures take place also needs to be considered to improve the ecological validity of measures. Although outside the scope of this review, future research should consider the influence of factors such as training and fatigue on impairment measures, as well as how susceptible the measure is to intentional misrepresentation.

## Conclusions

The development of evidence-based classification systems is a crucial step in ensuring legitimate and competitive opportunities for Para athletes. Therefore, it was important to identify and investigate effective methods for measuring impairment and activity limitation, as well as their associations with performance. Although research in this area is limited, the current base of evidence can aid existing and new sports seeking to join the Paralympic Movement in formulating a testing battery that aligns with evidence-based protocols. Continued efforts by sport governing bodies to prioritise research in this area will further enhance the understanding of the impact of impairments on sport performance, ultimately leading to better decision making and increased credibility of classification systems in Para sport.

## Supplementary Information

Below is the link to the electronic supplementary material.

Supplementary file1 (PDF 163 KB)

Supplementary file2 (PDF 495 KB)

## Declarations

### Availability of data and material

The data used to inform this review have been disclosed in the article or in its accompanying Electronic Supplementary Material.
