# Supplementary material for: Guiding Evidence-Based Classification in Para Sporting Populations: A Systematic Review of Impairment Measures and Activity Limitations
Source: Sports Med. 2024 Nov 22;55(2):341–91. doi: 10.1007/s40279-024-02132-y (PMC11946994; doi:10.1007/s40279-024-02132-y)
Supplement: Supplementary file 1 — Supplementary file1 (PDF 163 KB) [file 40279_2024_2132_MOESM1_ESM.pdf]

Electronic Supplementary Material – Online Resource 1

Search Strategy

CONTENTS

1A Search Strategy MEDLINE 2

2A Search Strategy Embase 4

3A Search Strategy Web of Science 6

4A Search Strategy SPORTDiscus with Full Text 7

5A Search Strategy CINAHL 8

6A Search Strategy Scopus 9

1A Search Strategy MEDLINE

Ovid MEDLINE(R) ALL <1946 to December 29, 2023>

1	Exercise Test/ (69209)
2	exercise test*.mp. (81232)
3	strength test*.mp. (7328)
4	isometric test*.mp. (283)
5	speed test*.mp. (1054)
6	agility test*.mp. (545)
7	exp "Range of Motion, Articular"/ (60804)
8	"range of motion*".mp. (86328)
9	balance test*.mp. (3376)
10	power test*.mp. (755)
11	coordination test*.mp. (379)
12	activity limitation*.mp. (4017)
13	functional test*.mp. (8476)
14	tapping task*.mp. (1218)
15	Visual Acuity*.mp. (122505)
16	Visual fields*.mp. (35362)
17	Visual function*.mp. (15972)
18	mental processes*.mp. or exp Mental Processes/ (1227216)
19	cognition*.mp. or cognition/ (263769)
20	intelligence*.mp. or intelligence tests/ (130599)
21	Neuropsychological Tests/ or neuropsychological test*.mp. (111744)
22	Executive function/ or executive function*.mp. (44460)
23	Psychomotor performance/ or psychomotor performance*.mp. (69570)
24	1 or 2 or 3 or 4 or 5 or 6 or 7 or 8 or 9 or 10 or 11 or 12 or 13 or 14 or 15 or 16 or 17 or 18 or 19 or 20 or 21 or 22 or 23 (1721005)
25	exp Intellectual Disability/ or intellectual disability*.mp. (116702)
26	intellectual impairment*.mp. (1719)
27	cognitive impairment*.mp. (95646)
28	visual impairment*.mp. (15719)
29	vision impairment*.mp. (3064)
30	vision disorders*.mp. (31251)
31	physical impairment*.mp. (3021)

32 brain impairment*.mp. (493)

33 Brain Injuries/ or brain injury*.mp. (110647)

34 Spinal Cord Injuries/ or spinal cord injury*.mp. (58709)

35 hemiplegia/ or paraplegia/ or quadriplegia/ (31767)

36 hemiplegia*.mp. (17137)

37 paraplegia*.mp. (22658)

38 quadriplegia*.mp. (10294)

39 coordination impairment*.mp. (216)

40 exp Ataxia/ or ataxia*.mp. (53118)

41 exp Muscle Hypertonia/ or hypertonia*.mp. (15395)

42 muscle hypertonia*.mp. (1193)

43 Athetosis/ or athetosis*.mp. (1695)

44 Cerebral Palsy/ or cerebral palsy*.mp. (32688)

45 short stature*.mp. (14156)

46 exp Dwarfism/ or dwarfism*.mp. (20085)

47 impaired muscle power*.mp. (9)

48 impaired range of motion*.mp. (39)

49 leg length difference*.mp. (167)

50 limb deficienc*.mp. (602)

51 25 or 26 or 27 or 28 or 29 or 30 or 31 or 32 or 33 or 34 or 35 or 36 or 37 or 38 or 39 or 40 or 41 or 42 or 43 or 44 or 45 or 46 or 47 or 48 or 49 or 50 (574775)

52 exp disabled persons/ or amputees/ or para-athletes/ (74271)

53 disabilit*.tw. (246859)

54 disab*.mp. (415718)

55 amputee*.mp. (8139)

56 exp Sports/ (219882)

57 (Archery or Athletics or Badminton or basketball or Boccia or Canoe or Cycling or Equestrian or fencing or football or Goalball or Judo or Powerlifting or Rowing or rugby or soccer or Shooting or swimming or Table tennis or Taekwondo or Triathlon or volleyball or tennis or Alpine skiing or Biathlon or Cross-country skiing or ice hockey or Snowboard or curling).tw. (166117)

58 Sports for Persons with Disabilities/ (327)

59 "sport for the disabled*".mp. (24)

60 ("sport for the disabled*" or para-sport* or para-athletic* or wheelchair sports* or para-swim* or para sport* or para athletic* or para swim*).mp. (323)

61 56 or 57 or 58 or 59 or 60 (337346)

62 24 and 51 and 61 (3006)

2A Search Strategy Embase

(1947-1973, 1974 – December 29, 2023)

1	Exercise Test/ (66089)
2	exercise test*.mp. (89584)
3	strength test*.mp. (9318)
4	isometric test*.mp. (368)
5	speed test*.mp. (1622)
6	agility test*.mp. (596)
7	exp "Range of Motion, Articular"/ (65723)
8	"range of motion*".mp. (88832)
9	balance test*.mp. (4781)
10	power test*.mp. (949)
11	coordination test*.mp. (566)
12	activity limitation*.mp. (6053)
13	functional test*.mp. (14026)
14	tapping task*.mp. (1604)
15	Visual Acuity*.mp. (180299)
16	Visual fields*.mp. (9500)
17	Visual function*.mp. (22876)
18	mental processes*.mp. or exp Mental Processes/ (4747003)
19	cognition*.mp. or cognition/ (393045)
20	intelligence*.mp. or intelligence tests/ (174289)
21	Neuropsychological Tests/ or neuropsychological test*.mp. (84354)
22	Executive function/ or executive function*.mp. (77795)
23	Psychomotor performance/ or psychomotor performance*.mp. (26519)
24	1 or 2 or 3 or 4 or 5 or 6 or 7 or 8 or 9 or 10 or 11 or 12 or 13 or 14 or 15 or 16 or 17 or 18 or 19 or 20 or 21 or 22 or 23 (5221641)
25	exp Intellectual Disability/ or intellectual disability*.mp. (650281)
26	intellectual impairment*.mp. (41507)
27	cognitive impairment*.mp. (152344)
28	visual impairment*.mp. (74520)
29	vision impairment*.mp. (4091)
30	vision disorders*.mp. (1457)
31	physical impairment*.mp. (4397)

32 brain impairment*.mp. (707)

33 Brain Injuries/ or brain injury*.mp. (192410)

34 Spinal Cord Injuries/ or spinal cord injury*.mp. (81086)

35 hemiplegia/ or paraplegia/ or quadriplegia/ (70419)

36 hemiplegia*.mp. (27608)

37 paraplegia*.mp. (41640)

38 quadriplegia*.mp. (22900)

39 coordination impairment*.mp. (316)

40 exp Ataxia/ or ataxia*.mp. (106349)

41 exp Muscle Hypertonia/ or hypertonia*.mp. (58992)

42 muscle hypertonia*.mp. (7363)

43 Athetosis/ or athetosis*.mp. (2016)

44 Cerebral Palsy/ or cerebral palsy*.mp. (52340)

45 short stature*.mp. (28758)

46 exp Dwarfism/ or dwarfism*.mp. (11606)

47 impaired muscle power*.mp. (11)

48 impaired range of motion*.mp. (57)

49 leg length difference*.mp. (197)

50 limb deficienc*.mp. (744)

51 25 or 26 or 27 or 28 or 29 or 30 or 31 or 32 or 33 or 34 or 35 or 36 or 37 or 38 or 39 or 40 or 41 or 42 or 43 or 44 or 45 or 46 or 47 or 48 or 49 or 50 (1321114)

52 exp disabled persons/ or amputees/ or para-athletes/ (57233)

53 disabilit*.tw. (355585)

54 disab*.mp. (523314)

55 amputee*.mp. (9452)

56 exp Sports/ (222167)

57 (Archery or Athletics or Badminton or basketball or Boccia or Canoe or Cycling or Equestrian or fencing or football or Goalball or Judo or Powerlifting or Rowing or rugby or soccer or Shooting or swimming or Table tennis or Taekwondo or Triathlon or volleyball or tennis or Alpine skiing or Biathlon or Cross-country skiing or ice hockey or Snowboard or curling).tw. (187641)

58 Sports for Persons with Disabilities/ (470)

59 "sport for the disabled*".mp. (42)

60 ("sport for the disabled*" or para-sport* or para-athletic* or wheelchair sports* or para-swim* or para sport* or para athletic* or para swim*).mp. (370)

61 56 or 57 or 58 or 59 or 60 (340778)

62 24 and 51 and 61 (5905)

3A Search Strategy Web of Science

((ALL=("exercise test*" OR "strength test*" OR "isometric test*" OR "speed test*" OR "agility test*" OR "range of motion*" OR "balance test*" OR "power test*" OR "coordination test*" OR "activity limitation*" OR "functional test*" OR "tapping task*" OR "visual acuity*" OR "visual fields*" OR "visual function*" OR "cognition*" OR "mental processes*" OR "executive function*" OR "intelligence*" OR "intelligence tests*" OR "neuropsychological tests*" OR "psychomotor performance*")) AND ALL=("intellectual disability*" OR "intellectual impairment*" OR “cognitive impairment*” OR "visual impairment*" OR “vision impairment*” OR "vision disorders*" OR "physical impairment*" OR "brain impairment*" OR "brain inj*" OR "spinal cord inj*" OR "hemiplegia*" OR "paraplegia*" OR "quadriplegia*" OR "coordination impairment*" OR "ataxia*" OR "muscle hypertonia*" OR "hypertonia*" OR "athetosis*" OR "cerebral palsy*" OR "short stature*" OR "dwarfism*" OR "impaired muscle power*" OR "impaired range of motion*" OR "leg length difference*" OR "limb deficienc*" OR "disabled persons*" OR "amputee*" OR "para-athletes" OR "disabilit*" OR "disab*")) AND ALL=("sport*" OR "archery*" OR "athletics*" OR "badminton*" OR "basketball*" OR "boccia*" OR "canoe*" OR "cycling*" OR "equestrian*" OR "fencing*" OR "football*" OR "goalball*" OR "judo*" OR "powerlifting*" OR "rowing*" OR "rugby*" OR "soccer*" OR "shooting*" OR "swimming*" OR "table tennis*" OR "taekwondo*" OR "triathlon*" OR "volleyball*" OR "tennis*" OR "skiing*" OR "biathlon*" OR "ice hockey*" OR "snowboard*" OR "curling*" OR "sports for persons with disabilities*" OR "sport for the disabled*" OR "para-sport*" OR "para-athletic*" OR "wheelchair sports*" OR "para-swim*" OR "para sport*" OR "para athletic*" OR "para swim*")

4A Search Strategy SPORTDiscus with Full Text

	Query	Limiters/Expanders	Results
S1	(MH "Exercise Test+") OR "exercise test*" OR "strength test*" OR "isometric test*" OR "speed test*" OR "agility test*" OR (MH "Range of Motion") OR "range of motion*" OR "balance test*" OR "power test*" OR "coordination test*" OR "activity limitation*" OR "functional test*" OR "tapping task*" OR "visual acuity*" OR "Visual fields*" OR "visual function*" OR "cognition*" OR "intelligence*" OR "intelligence test*" OR "neuropsychological test*" OR "executive function*" OR "psychomotor performance*"	Search modes: Boolean/phrase	69,976
S2	(MH "Intellectual Disability+") OR "intellectual disability*" OR "intellectual impairment*" OR "cognitive impairment*" OR (MH "Brain Injuries+") OR "brain injury*" OR (MH "Spinal Cord Injuries+") OR "spinal cord injury*" OR (MH "Hemiplegia") OR "hemiplegia*" OR (MH "Paraplegia+)" OR "paraplegia*" OR (MH "Quadriplegia+") OR "quadriplegia*" OR "coordination impairment*" OR (MH "Ataxia+") OR "ataxia*" OR (MH "Muscle Hypertonia+") OR "muscle hypertonia*" OR "hypertonia*" OR "athetosis*" OR (MH "Cerebral Palsy") OR "cerebral palsy*" OR "short stature*" OR (MH "Dwarfism+") OR "dwarfism" OR "impaired muscle power*" OR "impaired range of motion*" OR "leg length difference*" OR "limb deficienc*" OR "disabled persons*" OR (MH "Amputees") OR "amputee*" OR "para-athletes*" OR "disabilit*" OR "disab*" OR "physical impairment*" OR "vision impairment*" OR "visual impairment*" OR "vision disorder*"	Search modes: Boolean/phrase	89,012
S3	(MH "Sports+") OR (MH "Sports for Persons With Disabilities+") OR "archery*" OR "athletics*" OR "badminton*" OR "basketball*" OR "boccia*" OR "canoe*" OR "cycling*" OR "equestrian*" OR "fencing*" OR "football*" OR "goalball*" OR "judo*" OR "powerlifting*" OR "rowing*" OR "rugby*" OR "soccer*" OR "shooting*" OR "swimming*" OR "table tennis*" OR "taekwondo*" OR "triathlon*" OR "volleyball*" OR "tennis*" OR "alpine skiing*" OR "biathlon*" OR "cross-country skiing" OR "ice hockey*" OR "snowboard*" OR "curling*" OR "sport for the disabled*" OR "para-sport*" OR para-athletic*" OR "wheelchair sports*" OR "para-swim*" OR "para sport*" OR "para athletic*" OR "para swim*"	Search modes: Boolean/phrase	699,071
S4	S1 AND S2 AND S3	Search modes: Boolean/phrase<br><br>Limiters - publication type: academic journal	543

5A Search Strategy CINAHL

	Query	Limiters/Expanders	Results
S1	(MH "Exercise Test+") OR "exercise test*" OR "strength test*" OR "isometric test*" OR "speed test*" OR "agility test*" OR (MH "Range of Motion") OR "range of motion*" OR "balance test*" OR "power test*" OR "coordination test*" OR "activity limitation*" OR "functional test*" OR "tapping task*" OR "visual acuity*" OR "Visual fields*" OR "visual function*" OR "cognition*" OR "intelligence*" OR "intelligence test*" OR "neuropsychological test*" OR "executive function*" OR "psychomotor performance*"	Search modes: Boolean/phrase	298, 238
S2	(MH "Intellectual Disability+") OR "intellectual disability*" OR "intellectual impairment*" OR "cognitive impairment*" OR (MH "Brain Injuries+") OR "brain injury*" OR (MH "Spinal Cord Injuries+") OR "spinal cord injury*" OR (MH "Hemiplegia") OR "hemiplegia*" OR (MH "Paraplegia+)" OR "paraplegia*" OR (MH "Quadriplegia+") OR "quadriplegia*" OR "coordination impairment*" OR (MH "Ataxia+") OR "ataxia*" OR (MH "Muscle Hypertonia+") OR "muscle hypertonia*" OR "hypertonia*" OR "athetosis*" OR (MH "Cerebral Palsy") OR "cerebral palsy*" OR "short stature*" OR (MH "Dwarfism+") OR "dwarfism" OR "impaired muscle power*" OR "impaired range of motion*" OR "leg length difference*" OR "limb deficienc*" OR "disabled persons*" OR (MH "Amputees") OR "amputee*" OR "para-athletes*" OR "disabilit*" OR "disab*" OR "physical impairment*" OR "vision impairment*" OR "visual impairment*" OR "vision disorder*"	Search modes: Boolean/phrase	382,217
S3	(MH "Sports+") OR (MH "Sports for Persons With Disabilities+") OR "archery*" OR "athletics*" OR "badminton*" OR "basketball*" OR "boccia*" OR "canoe*" OR "cycling*" OR "equestrian*" OR "fencing*" OR "football*" OR "goalball*" OR "judo*" OR "powerlifting*" OR "rowing*" OR "rugby*" OR "soccer*" OR "shooting*" OR "swimming*" OR "table tennis*" OR "taekwondo*" OR "triathlon*" OR "volleyball*" OR "tennis*" OR "alpine skiing*" OR "biathlon*" OR "cross-country skiing" OR "ice hockey*" OR "snowboard*" OR "curling*" OR "sport for the disabled*" OR "para-sport*" OR para-athletic*" OR "wheelchair sports*" OR "para-swim*" OR "para sport*" OR "para athletic*" OR "para swim*"	Search modes: Boolean/phrase	110,786
S4	S1 AND S2 AND S3	Search modes: Boolean/phrase<br><br>Limiters - publication type: academic journal	1769

6A Search Strategy Scopus

((TITLE-ABS-KEY("sport*" OR "archery*" OR "athletics*" OR "badminton*" OR "basketball*" OR "boccia*" OR "canoe*" OR "cycling*" OR "equestrian*" OR "fencing*" OR "football*" OR "goalball*" OR "judo*" OR "powerlifting*" OR "rowing*" OR "rugby*" OR "soccer*" OR "shooting*" OR "swimming*" OR "table tennis*" OR "taekwondo*" OR "triathlon*" OR "volleyball*" OR "tennis*" OR "skiing*" OR "biathlon*" OR "ice hockey*" OR "snowboard*" OR "curling*") OR TITLE-ABS-KEY("sports for persons with disabilities*" OR "sport for the disabled*" OR "para-sport*" OR "para-athletic*" OR "wheelchair sports*" OR "para-swim*" OR "para sport*" OR "para athletic*" OR "para swim*"))) AND ((TITLE-ABS-KEY("exercise test*" OR "strength test*" OR "isometric test*" OR "speed test*" OR "agility test*" OR "range of motion*" OR "balance test*" OR "power test*" OR "coordination test*" OR "activity limitation*" OR "functional test*" OR "tapping task*" OR "visual acuity*" OR "visual field*" OR “visual function*” OR "executive function*" OR "cognition*" OR “intelligence*” OR “intelligence tests*” OR "mental processes*" OR "neuropsychological test*" OR "psychomotor performance*") AND TITLE-ABS-KEY("intellectual disability*" OR "intellectual impairment*" OR "cognitive impairment*" OR "visual impairment*" OR "vision impairment*" OR "vision disorders*" OR "physical impairment*" OR "brain impairment*" OR "brain inj*" OR "spinal cord inj*" OR "hemiplegia*" OR "paraplegia*" OR "quadriplegia*" OR "coordination impairment*" OR "ataxia*" OR "muscle hypertonia*" OR "hypertonia*" OR "athetosis*" OR "cerebral palsy*" OR "short stature*" OR "dwarfism*" OR "impaired muscle power*" OR "impaired range of motion*" OR "leg length difference*" OR "limb deficienc*" OR "disabled persons*" OR "amputee*" OR "para-athletes" OR "disabilit*" OR "disab*"))) AND (LIMIT-TO (SRCTYPE,"j"))
