# Supplementary material for: Guiding Evidence-Based Classification in Para Sporting Populations: A Systematic Review of Impairment Measures and Activity Limitations
Source: Sports Med. 2024 Nov 22;55(2):341–91. doi: 10.1007/s40279-024-02132-y (PMC11946994; doi:10.1007/s40279-024-02132-y)
Supplement: Supplementary file 2 — Supplementary file2 (PDF 495 KB) [file 40279_2024_2132_MOESM2_ESM.pdf]

Electronic Supplementary Material – Online Resource 2

Methods of Assessment

CONTENTS

2A Methods of assessment for eligible studies regarding Strength Impairment 2

2B Methods of assessment for eligible studies regarding Coordination Impairment 8

2C Methods of assessment for eligible studies regarding ROM Impairment 12

2D Methods of assessment for eligible studies regarding Intellectual Impairment... 16

2E Methods of assessment for eligible studies regarding Vision Impairment 19

2F Methods of assessment for eligible studies regarding Activity Limitation Measures................................ 23

2G Reference List.. 31

2A Methods of assessment for eligible studies regarding Strength Impairment

Study	Test	Method of Assessment	Outcome Measure	Equipment
Altmann et al. [31]	Static sitting balance	Participants seated (hips and knees 90°, ankles neutral), keeping arms across chest while sitting as still as possible for 30s during two conditions; stable surface (5cm foam cushion), unstable surface (inflated cushion – buttocks raised 2cm)	Sway area in mm^2	Chair mounted on AMTI forceplate (model no. OR6-7MA-1000)
	Dynamic sitting balance	Participants seated (hips and knees 90°, ankles neutral) on foam cushion. Maximal trunk inclination assessed in four directions: posterior-anterior, left-right lateral 90° from sagittal plane; right forward-left backward and left forward-right backward 45° from sagittal plane.	Excursion of centre of pressure displacement in mm	
Altmann et al. [31] & Altmann et al. [32]	Isometric trunk strength	Participants seated on height adjustable plinth (hips, thighs and ankles strapped to plinth). Harness connected to load cell placed around chest. Isometric strength assessed during forward, left, right (feet unsupported) [31, 32] and backward directions (feet supported and unsupported) [31]	Force in Newtons	Load cell (model no. K25); RMP Rheinmetall Mess-und Pruftechnik GmbH
Beckman et al. [33]	Leg extensor strength	Participant seated. 120° knee extension, 60° hip flexion. Foot of testing leg attached to testing plate with non-elastic strapping and non-testing leg on ground. Required joint angles determined by marking landmarks and real time video feedback. Both limbs assessed.	Force in Newtons	Isometric strength rig with S type load cell attached.<br>Marking pen<br>Sony Camera<br>Dartfish Software (version 4.0.9.0, Dartfish, Switzerland)
	Leg flexor strength	Participant seated. 120° knee extension, 70° hip flexion. Foot of testing leg on testing plate and non-testing leg on ground. Required joint angles determined by marking landmarks and real time video feedback. Both limbs assessed.		
	Plantarflexor strength	Participant seated. Full knee extension, ankle in neutral to 5° dorsiflexion. Foot of testing leg on testing plate and non-testing leg on ground. Required joint angles determined by marking landmarks and real time video feedback. Both limbs assessed.		

Study	Test	Method of Assessment	Outcome Measure	Equipment
Connick et al. [34]	Left & right arm extension	Participant seated with trunk supported (backrest/strapping). Load cell placed at shoulder height with testing hand positioned 90° shoulder abduction, 45° horizontal shoulder flexion, 120° elbow extension. Position confirmed with goniometer.	Force in Newtons	S-type load cell (Scale Components, Queensland, Australia) attached to strength rig, connected to a Musclelab unit
	Arm extension & trunk flexion	Participant seated. Exerted maximal force while maintaining trunk in 30° flexion, 90° shoulder abduction, 45° horizontal adduction, 120° elbow extension. Real time video feedback determined trunk angle.		S-type load cell (Scale Components, Queensland, Australia) attached to strength rig, connected to a Musclelab unit<br>Video Camera (Sony DCR-HC19E PAL, Tokyo, Japan)<br>Dartfish software (Dartfish ProSuite v. 4.0, Fribourg, Switzerland)
	Isolated trunk flexion	Participant seated with chest harness attached to load cell. Positioned in 45° forward trunk flexion, arms across chest. Real time video feedback determined trunk angle. Instructed to move trunk towards knees while maintaining trunk position.		
	Combined pronation & grip strength	Participant seated in own wheelchair. Elbow 90°, shoulder in neutral.		Wall mounted isometric wrist dynamometer (Baseline Evaluation Instruments, New York, USA)
Domínguez-Díez et al. [35]	Isometric propulsion strength test	Participant seated in own wheelchair. Load cell connected to webbing above wheels' axis. Wheelchair held in place to prevent wheels from turning. Elbow flexion 90° for push action and 120° for pull action. Trunk flexion between 70-80° to the horizontal. Angles confirmed with goniometer. Real time video feedback determined correct positioning during trials.	Force in Newtons	Strain gauge load cell (Globus Iso Control, Codogné, Italy)<br>Goniometer (Lafayette model 01135)<br>LabVIEW (v.2010, National Instruments, Austin, USA)

Study	Test	Method of Assessment	Outcome Measure	Equipment
Hogarth et al. [18]	Shoulder flexion	Participant seated. Trunk supported with backrest/strapping. Hand placed in cuff with arm facing forward in neutral position, elbow, and shoulder also in neutral. Both limbs assessed.	Force in Newtons	S type load cell attached to custom made aluminium frame
	Shoulder extension	Participant seated. Trunk supported with backrest/strapping. Cuff positioned at shoulder height. Palm placed downwards in cuff, elbow neutral, 90° shoulder flexion. Position confirmed with digital inclinometer. Both limbs assessed.		
	Hip flexion	Participant in supine on plinth. Ankle placed in cuff, arms across chest, hip, and knee in horizontal. Both limbs assessed.		
	Hip extension	Participant in supine on plinth. Ankle placed in cuff, so hip was in 15° flexion with knee and ankle in neutral. Position confirmed with digital inclinometer. Both limbs assessed.		
Hyde et al. [36]	Throwing arm push test	Participants seated with backrest. Hip and knee secured with straps at 90°. Arm positioned parallel to the floor with 90° shoulder flexion, 45° horizontal shoulder flexion and 120° elbow extension	Force in Newtons	S type load cell (Scale Components, Slacks Creek, QLD, Australia)
	Push/pull synergy test	Participants seated. No backrest. Hip and knee secured with straps at 90°. Shoulder flexion 90°, 45° horizontal shoulder flexion, 120° elbow extension. Non-dominant hand gripped pole. Dominant hand on load cell.		
	Trunk flexion test	Participants seated and strapped to seat. Load cell attached to box on floor. Both hands on load cell, 45° trunk flexion, 120° elbow extension. Required to maintain position and push down on load cell using trunk muscles.		

Study	Test	Method of Assessment	Outcome Measure	Equipment
Hyde et al. [36]	Grip strength	Participant seated. Elbow flexed at 90° with dynamometer held against trunk. Both hands assessed.	Measured in kg	Handheld dynamometer (Smedlay's Dynamometer, Fabrication Enterprises, White Plains NY, USA)
Liljedahl et al. [37]	Isometric leg push/pull	Participants seated upright with arms across chest. Belt fastened across hips and non-tested foot on the floor. Tested leg in 50° knee flexion. Push test measured the positive force applied to footrest. Pull test (foot strapped to footrest) measured negative forced applied to footrest. Both limbs assessed.	Peak force measured in N/kg	Custom built strength setup with 3D piezoelectric force transducers (type 9347B; Kistler Instruments AG, Switzerland) connected to an amplifier (type 9865E; Kistler Instruments AG).
	Dynamic leg push/pull	Participants seated. Both tests performed from a dead start. Tested leg clipped to pedal with non-tested leg positioned with hip extended and toes placed on a box adjacent to rear wheel. Dynamic push participant pushed down on the pedal to the bottom position (180°). The dynamic pull was performed by pulling the pedal from the bottom to top position. The starting resistance was 100N until participant no longer improved peak power. If participant could not overcome 100N it was lowered. Both limbs assessed.	Peak power expressed as W/kg	Personal road bicycle mounted to a cycling ergometer (Cyclus2; RBM Electronics, Germany).
Liu et al. [38]	Isometric trunk strength	Participants seated. Hips, thighs, and ankles strapped to seat. No backrest, feet unsupported. Harness connected to dynamometer positioned around chest. Arms across chest. Isometric strength assessed during forward, left, and right directions.	Force in Newtons	Height adjustable dynamometer (range: 600N, graduation: 0.2N. OCS-0060L, Angxuan) attached to wall.

Study	Test	Method of Assessment	Outcome Measure	Equipment
Mason et al. [39], Mason et al. [40]	Shoulder flexion/extension	Participants seated. Elbow flexed 90°, upper arm in neutral. Cuff positioned around distal part of humerus. Both limbs assessed.	Force in Newtons	Isometric strength rig, with S type load cell extended from frame attached to participant via Velcro cuff. Force data collected through Musclelab unit.
	Elbow flexion/extension	Participants seated. Elbow flexed 90◦, upper arm in neutral. Cuff positioned in centre of wrist joint. Both limbs assessed.		
Mason et al. [40]	Push test	Wheels attached to high friction rubber. Load cell connected to bar behind backrest of wheelchair. Cable connecting wheelchair to load cell parallel to ground. Asked to pull at position on wheel where they believed greatest force would be generated.	Force in Newtons	Isometric strength rig, with S type load cell extended from frame attached to participant via Velcro cuff. Force data collected through Musclelab unit.
	Pull test	Wheels attached to high friction rubber. Load cell attached to pick bar on front of wheelchair. Cable connecting wheelchair to load cell parallel to ground. Asked to pull at position on wheel where they believed greatest force would be generated.		

Study	Test	Method of Assessment	Outcome Measure	Equipment
Rosso et al. [41]	Balance perturbations	Participants sit ski was fitted to a 94cm x 84cm motorised plate, positioned on two parallel tracks (140cm long). Participants received 12 random perturbations (6 backwards, 6 forwards); maximum acceleration 2.5 $m \cdot s^{-2}$; maximum velocity 0.5 $m \cdot s^{-1}$. Participants instructed to maintain trunk stability and upper limbs in neutral position. Motion analysis system used to calculate trunk angle.	Delay (ms) between onset of sledge acceleration and shoulder acceleration<br>Delay (ms) between onset of shoulder acceleration and time when trunk inverted the motion<br>Trunk angle (°) before perturbation; Trunk ROM (°) 150ms after onset of shoulder acceleration; Trunk ROM (°) when trunk inverted the motion.	Motorised Plate (University of Jyvaskyla, Finland)<br>Electromechanical servo actuator (IndraDyn S MSK; Bosh Rexroth, Lohram Main, Germany)<br>LabVIEW custom-made script (LabVIEW 8.5; National Instruments, Austin, TX)<br>8 VICON Cameras/ Nexus Software (VICON Motion System Ltd. Oxford, UK)
Vanlandewijck et al. [42]	Arm isometric strength	Participants seated. Trunk, pelvis, and thigh strapped to seat with backrest support. Load cell positioned at shoulder height in front of sternum. Both elbows in 60° flexion, palms at same height of rotation centre of glenohumeral joint. Elbow angle confirmed with goniometer.	Force in Newtons	Custom built strength rig with S type load cell attached to Musclelab unit.<br>Goniometer
	Trunk isometric strength	Participants seated. Pelvis and thighs strapped to seat. No backrest. Trunk 30◦ flexion, arms same position as arm strength test. Elbow angle confirmed with goniometer. Real time video feedback determined trunk angle.	Force in Newtons	Custom built strength rig with S type load cell attached to Musclelab unit.<br>Goniometer<br>Video camera (Sony DCR-HC19E PAL, Tokyo, Japan)<br>Dartfish software (Dartfish Prosuite v.4.0, Fribourg, Switzerland)

2B Methods of assessment for eligible studies regarding Coordination Impairment

Study	Test	Method of Assessment	Outcome Measure	Equipment
Altmann et al. [43]	Spiral test	Spiral with 8 turns (width 1cm) printed on A4 sheet of paper. Line drawn between the black lines until middle of spiral was reached. Participants seated.	Time (s) to complete movement. Penalty +3s each line touch, +5s each line cross.	Pen (to draw line)<br>Spiral test form<br>Video camera
	Finger-nose test	Participants asked to touch their nose and 1 x 1cm target on wall (level with nose) with tip of index finger as quickly as possible. Participants seated.	Number of correct repetitions performed in 20s.	Video camera
	Repetitive movement tests (shoulder, elbow, forearm, wrist, fingers)	One side performed at a time. Participants seated.<br>*Shoulder*: elevation from start position to 135° or maximum passive range of movement if less.<br>*Elbow:* elbow flexion to 135° or maximum passive range of movement if less.<br>*Forearm: p*ronation until palm on table<br>*Wrist:* 90° wrist flexion to 90° wrist extension or max passive range of movement if less.<br>*Finger:* finger flexion to full fist or max passive range of movement if less.	Number of correct repetitions performed in 20s.	Video camera
	Coordination deduction	No specific test. Determined from observations by classifiers of wheelchair activities on and off court.	Maximum arm score minus allocated arm score.	N/A
Connick et al. [44]	Reciprocal unilateral tapping with 5cm target	Participants seated. Copper attached to plantar surface of big toe. Boards positioned at 20° to the horizontal. Participants moved big toe between the marked 5cm area as quickly and as accurately as possible. Trial lasted 15s. Both limbs tested.	Mean movement time (s)	Two custom made 17.5cm x 12cm fibreglass printed circuit boards<br>Musclelab software

Study	Test	Method of Assessment	Outcome Measure	Equipment
Connick et al. [44]	Reciprocal unilateral tapping with 12cm target	Participants seated. Copper attached to plantar surface of big toe. Boards positioned at 20° to the horizontal. Participants moved big toe between the marked 12cm area as quickly and as accurately as possible. Trial lasted 15s. Both limbs tested.	Mean movement time (s)	Two custom made 17.5cm x 12cm fibreglass printed circuit boards<br>Musclelab software
	Reciprocal bilateral tapping	Participants seated. Copper attached to plantar surface of big toe. Four boards positioned in 2x2 formation in front of participant (Back boards tilted at 15°, front boards tilted at 30°). Participants tapped right front board with right big toe, left back board with left big toe, right back board with right big toe, front left board with left big toe (one cycle). Trial lasted 15s.		
Hogarth et al. [19]	Bilateral upper limb tapping	Participants seated with backrest. Performed with arms extended, hands in fists and index finger extended. Participants alternatively tapped the target on the two pads with left and right fingers as quickly and as accurately as possible. Trials lasted 15s.	Mean movement time (ms)	Custom made wireless tapping pads with 19.5 x 10cm target<br>Musclelab software<br>Video camera
	Dominant and non-non-dominant upper limb tapping	Participants seated with backrest. Testing arm extended, hand in fists and index finger extended. Non-testing arm rested on thigh. Participants alternated tapping between the two targets as quickly and as accurately as possible. Trials lasted 15s.	Mean movement time (ms)<br><br>Lower limb symmetry score (ratio of dominant limb tapping to non-dominant limb tapping).	
	Bilateral lower limb tapping	Participants seated with backrest. Tapping pads secured to footrest at 30° incline positioned in line with midline of participant. Legs extended, participants alternatively tapped the target with their big toe as quickly and as accurately as possible. Trials lasted 15s.	Mean movement time (ms)	

Study	Test	Method of Assessment	Outcome Measure	Equipment
Hogarth et al. [19]	Dominant and non-dominant lower limb tapping	Participants seated with backrest. Tapping pads secured to footrest at 30° incline positioned in line with midline of participant. Non testing leg relaxed. Testing leg extended. Participants moved big toe to alternate tapping between the two pads as quickly and as accurately as possible. Trials lasted 15s.	Mean movement time (ms)<br><br>Lower limb symmetry score (ratio of dominant limb tapping to non-dominant limb tapping)	Custom made wireless tapping pads with 19.5 x 10cm target<br>Musclelab software<br>Video camera
Maia et al. [45]	Motor coordination test	Participants supine on bed. Triaxial accelerometer attached to dorsal surface of both wrists. Adjustable horizontal bar positioned at 80% of participants maximal active shoulder range of motion. Instructed to alternatively perform flexion/extension of shoulders in vertical plane, palm facing downwards, elbow extended, touching target with back of hand in the same zone. Frequency of movement was controlled with a metronome (30bpm and 120bpm).	*Smoothness of movement*<br>Total number of acceleration peaks over final 20s<br>*Rhythm error*<br>Discrepancy between expected and actual timing at the end of each arm cycle determined from video<br>*Movement accuracy*<br>Mean absolute distance (in zones) from the reference zone (zone touched with first touch)	GENEActiv tri-axial accelerometer (GENEActiv Action, Activinsights Ltd, Cambridgeshire, UK)<br>Digital metronome (Shenzhen Meideal Musical Instruments Co., Ltd, China)<br>Video camera (Sony HDR CX700, Sony Corporation, Japan)
Reina et al. [46]	Rapid heel-toe contacts	Participants seated. Alternate between heel and toe contacts for a total of 25 cycles (one cycle = one heel and one toe contact). Required to perform the test as fast as possible. Only contacts within the area were counted. Both feet assessed.	Time (s) to complete 25 correct cycles	Tapping platform (35 x 20cm) with yellow dot (5cm) used as contact spot.

Study	Test	Method of Assessment	Outcome Measure	Equipment
Roldan et al. [47]	Box and block test	Participants use throwing hand to transfer 25mm blocks from one section of box to another.	Number of blocks moved in a minute	Box and blocks<br>Video camera (Sony HDR-PJ410B)<br>Stopwatch (Casio HS-30W-1V)
	Box and ball test	Participants use throwing hand to transfer boccia balls (274mm, 278g) from one section of box to another	Number of boccia balls moved in a minute	Box and boccia balls<br>Video camera (Sony HDR-PJ410B)<br>Stopwatch (Casio HS-30W-1V)
	Discrete horizontal finger tapping	Wheelchair placed 10cm from plates. Participant's shoulder was aligned with starting plate. Non-throwing arm placed across chest. Metal thimble worn on index finger of dominant hands. On verbal command participants moved index finger between two plates (30cm apart) tapping the target area as quickly as possible for a total of 10 cycles	Average score of 10 tapping cycles (s)	Metal plates (30 x 20cm) with target area (18 x 5cm)
	Discrete vertical finger tapping	Plates arranged in a 'L' Shape (90°) with 30cm between plates. Non-throwing arm placed across chest. Metal thimble worn on index finger of dominant hand. Participants started with finger on the horizontal plate and moved on command to the vertical plate for a total of 10 cycles.		
	Discrete vertical tapping with ball	Participant seated and plates arranged in a 'L' shape with participants shoulder aligned with starting plate. Participants contacted plate with ball for a total of 10 cycles.		Metal plates (14 x 17cm) that worked on spring system
	Continuous vertical tapping with ball	Same setup as discrete task with ball except participants continually tapped alternatively between plates.	Number of contacts made in a minute.	

2C Methods of assessment for eligible studies regarding ROM Impairment

Study	Test	Method of Assessment	Outcome Measure	Equipment
Bjerkefors et al. [48]	Kinematic analysis of kayak performance	Kinematic data were collected for 20 paddle cycles at the highest possible intensity that could be maintained. (One cycle = time from catch to catch for each side). If Para-athletes used adaptive or straps, they were mounted on ergometer to replicate competition setup.	ROM or max/min peak angle were recorded for:<br>- *Shoulder:* flexion/extension, abduction/adduction, external/internal rotation<br>- *Elbow:* flexion/extension<br>- *Wrist:* dorsal/palmar flexion, ulnar and radial deviation.<br>- *Trunk:* flexion/extension, rotation.<br>- *Hip:* flexion/extension.<br>- *Knee:* flexion/extension.<br>- *Foot:* dorsal flexion/ plantar flexion.	Kayak ergometer (Dansprint ergometer; Dansprint ApS, Hvidovre, Denmark)<br>12 camera optoelectronic system (Oqus4; Qualisys AB, Gothenburg, Sweden)<br>Force transducer (Type 9311B; Kistler Instruments AG, Winterthur, Switzerland)
Connick et al. [44]	Maximum thigh flexion & heel pull distance	Participants supine on plinth, legs fully extended, feet against wall, maximally flexing the testing leg at knee and hip. Both limbs assessed.	*Maximum thigh flexion*<br>Angle between tested thigh and horizontal<br>*Heel pull*<br>Distance on non-tested leg between wall and position of heel on tested leg.	Segment angles measured with digital inclinometer (Lafayette Instrument, Lafayette, IN)<br>Segment lengths measured with segmometer (Rosscraft Innovations, Vancouver, CA).
	Maximum thigh extension	Standing on a platform, leaning against wall so testing leg can swing feely but non tested side fully supported. Both limbs assessed.	Angle between tested thigh and vertical	

Study	Test	Method of Assessment	Outcome Measure	Equipment
Connick et al. [44]	Dorsiflexion lunge	Maintaining heel contact on the ground, participant reached maximal dorsiflexion by lowering COM and flexing knee. Both limbs assessed.	Angle between tested tibia and horizontal	Segment angles measured with digital inclinometer (Lafayette Instrument, Lafayette, IN)<br><br>Segment lengths measured with segmometer (Rosscraft Innovations, Vancouver, CA
	Backward stepping lunge	Shank of one leg at 90° while contralateral leg was moved backwards as fast as possible. Both limbs assessed.	Distance between most anterior phalanx of back foot and heel of front foot	
Hyde et al. [36]	Kinematic analysis of seated throw performance	Kinematic data was collected with and without an assistive pole in both a standardised and self-selected condition.<br>Without pole (standardised): seat angle 18°, backrest height 18% of participants sitting height<br>With pole (standardised): seat angle 20°, backrest height 15% of participants seating height<br>With and without pole (self-selected): any variable individually altered by participant<br>Kinematic data collected for:<br>- Start of forward movement: elbow flexion (°)<br>- Cocking of throwing arm: max shoulder internal rotation angular velocity (°/s), trunk extension (°), maximum shoulder external rotation (°), ipsilateral trunk rotation (°)<br>- Arm acceleration: max shoulder internal rotation angular velocity (°/s), max elbow extension velocity (°/s), trunk angular velocity (°/s)<br>- Release velocity: hand speed (m/s), trunk flexion (°), elbow angle (°), contralateral trunk rotation (°)	ROM measured in degrees<br><br>Angular velocity measured in $m.s^{-1}$	Qualisys Motion Capture System (v2.2) (Gottenburg, Sweden)

Study	Test	Method of Assessment	Outcome Measure	Equipment
Liu et al. [38]	Trunk ROM	Participant seated with three belts across hips, knees, and ankles. Hands are placed across chest and markers placed on the acromion and greater trochanter. During the forward flexion test, participants are instructed to flex forward as far as possible (up to 45°) and hold maximal flexion for 3s with the test board secured. In the backward extension test participants instructed to extend back as slowly as possible (up to 45°) and hold maximal extension for 3s with the test board secured.	ROM measured in degrees	Video camera (Sony ILCE-7M2, HD 1080p)<br>Handheld digital goniometer (range: 360°: graduation: 0.05°: Sanliang Corp)
Nicholson et al. [49]	Bilateral shoulder flexion	Participant seated, supported by backrest with feet touching the ground. Strap around trunk. Instructed to raise both arms to achieve best 'streamline position'. Inclinometer placed on lateral aspect of upper arm and mid-way between acromion process and lateral epicondyle.	ROM measured in degrees	Acumar Digital Inclinometer (Lafayette Instrument Co. Lafayette, IN)<br>Universal goniometer (Baseline Evaluation Instruments, White Plains, NY)
	Bilateral shoulder abduction	Participant seated, supported by backrest with feet touching the ground. Strap around trunk. Instructed to raise both arms to achieve best 'streamline position'. Inclinometer placed on posterior aspect of upper arm and mid-way between acromion process and lateral epicondyle.		
	Elbow flexion and extension	Participant seated, feet touching the ground. Elbow flexion: bend elbow as far as possible. Elbow extension: fully straighten. Inclinometer placed on mid-line of posterior forearm after upper arm was aligned to the vertical. Goniometer axis aligned with lateral epicondyle, stationary arm on the lateral midline of the humerus in line with the acromion, moving arm along lateral radius in line with radial styloid process.		
	Lower limb streamline (Hip, knee, ankle extension)	Participants supine on plinth - feet and ankles off the bed. Participants instructed to achieve best 'streamline position' (hip and knee extension, ankle plantarflexion). Measurements performed at thigh, shank, and ankle.		

Study	Test	Method of Assessment	Outcome Measure	Equipment
Nicholson et al. [49]	Hip and knee flexion	Participants supine on bench. Instructed to actively bend the hip and knee towards the chest. Hip flexion measured at thigh mid-point. Knee flexion measured at shank mid-point. Knee flexion (goniometer) axis aligned with lateral knee joint line, stationary arm on lateral midline of femur in line with greater trochanter, moving arm along fibula in line with lateral malleolus.	ROM measured in degrees	Acumar Digital Inclinometer (Lafayette Instrument Co. Lafayette, IN)<br>Universal goniometer (Baseline Evaluation Instruments, White Plains, NY)
	Shoulder internal and external rotation	Supine on plinth, shoulder 90° abduction, 90° elbow flexion, forearm/ wrist neutral position. Inclinometer placed at the mid forearm projection on the posterior surface for internal rotation and interior surface for external rotation.		
	Prone shoulder extension	Prone position over plinth with body and head supported, feet in contact with ground. Non-test arm held by side. Test arm in shoulder extension with elbow in 90° flexion, avoiding trunk rotation. Test repeated with elbow in extension. Inclinometer placed on posterior upper arm at the projection between the acromion and lateral epicondyle.		
	Prone shoulder horizontal abduction	Prone position over plinth with body and head supported, feet in contact with ground. Non test arm by side. Test arm in horizontal shoulder adduction with elbow in 90° flexion, avoiding trunk rotation. Inclinometer placed on posterior upper arm at the projection between the acromion and lateral epicondyle.		
	Prone shoulder flexion	Prone position over plinth with body and head supported, feet in contact with ground. Non test arm by side. Test arm actively moved into shoulder flexion. Inclinometer placed on the posterior upper arm at the projection between the acromion and lateral epicondyle.		
	Trunk functional reach (forward, backwards, sideways)	Participant seated on plinth with feet supported, hips and knees at 90°. Arms across chest, participants flexed trunk forward, backwards, left, and right. The start and finish points of acromion from whiteboard were used as measure of functional reach.	Distance between starting and finishing position of acromion from whiteboard	Portable whiteboard<br>Tape measure

2D Methods of assessment for eligible studies regarding Intellectual Impairment

Study	Test	Cognitive Abilities	Method of Assessment	Outcome Measure	Equipment
Pineda et al. [50]	Cognitive Postural Dual Tasking	NA	Participants tried to remember as many objects as possible while trying to stand as still as possible. They were instructed to keep arms by their side and focus on a black circle displayed on a white background presented on an 89cm screen, situated on the wall 2m away at eye level.	Proportion of correct responses (%)<br>COP trajectory (mm)	Computer<br>Rocking board laid on a force plate
Van Biesen et al. [51], Van Biesen et al. [52], Van Biesen et al. [53]	Simple Reaction Time	Visual processing speed	Participant places index finger of dominant hand on the space bar of keyboard and taps it as soon as a white circle appears in the middle of a black screen at randomised time intervals [52,53].<br>Participant places index finger of dominant hand on right arrow and tap it as soon as arrow appears on screen at randomised time intervals [51].	Mean reaction time (ms)	Computer – keyboard
Van Biesen et al. [52], Van Biesen et al. [53]	Complex Reaction Time	Visual processing speed, attention	Similar to the simple reaction time test, this test involves additional stimuli (triangles and squares). Participant is instructed to tap only when circle appears.	Mean reaction time (ms)	Computer – keyboard
	Simple Visual Search	Processing speed	Participant seated in front of computer screen and rapidly taps the screen with their preferred hand in the middle of the circle when it appears on the screen (randomised time intervals and chosen spots)	Mean reaction time (ms)	Computer- touch screen
	Complex Visual Search	Processing speed, visual processing	Similar to simple visual search test but involves a distracting background (i.e., flickering dots) with the circle gradually becoming more visible	Mean reaction time (ms)	Computer- touch screen
	Corsi Memory	Working memory, spatial memory span	Participants required to reproduce randomly generated block-tapping sequences. Upon completion of the sequence presentation, all squares turned blue, serving as a cue for the participant to commence tapping the blocks in the same order as they were presented. The sequence length started with two blocks and increased by one for each correct recall and decreased by one if any error was made.	Best average score from 5 subsequent trials.	Computer- touch screen

Study	Test	Cognitive Abilities	Method of Assessment	Outcome Measure	Equipment
Van Biesen et al. [52], Van Biesen et al. [53]	Tower of London	Executive Functioning	Participants are required to replicate configurations in a minimal number of moves.	Number of correct items (max 18).	Computer- touch screen
	Block Design	Fluid reasoning/visual processing	Participants are required to replicate 2D block patterns with nine 3D cubes.	Score depends on correct items and speed (max 72).	3D cubes
	Matrix Reasoning	Inductive reasoning, mental rotation, pattern recognition	Participants are required to complete gridded patterns (multiple choice)	Number of correct items (max 35).	NA
	Finger tapping	Control test for psychomotor speed	Participants tap the spacebar on the keyboard with either a single finger or all fingers simultaneously, without maintaining continuous pressure on the key or alternating taps with multiple fingers. Both hands assessed.	Maximum number of taps per 10s.	Computer – keyboard
Van Biesen et al. [51]	Choice Reaction Time	Visual processing speed	Participant rested index finger of dominant hand close to arrow keyboard keys. They were instructed to press the arrow key corresponding to the direction of the arrow that appeared on the screen as quickly as possible. A new arrow appeared immediately after each response.	Total number of responses in 30s. Number of correct and incorrect taps were automatically registered.	Computer - keyboard
Van Biesen et al. [51], Van Biesen et al. [55]	Adapted Flanker Task	Response Inhibition	Participants instructed to rest their dominant hand’s index finger near the keyboard arrow keys. Their task was to solely focus on the central arrow that appears on the screen. They were instructed to respond as fast as possible by matching the arrow key corresponding to the central arrow while disregarding the other four arrows acting as distractors.	Total number of responses in 30s. Number of correct and incorrect taps were automatically registered.	Computer – keyboard

Study	Test	Cognitive Abilities	Method of Assessment	Outcome Measure	Equipment
Van Biesen et al. [54]	Adapted Multiple Object Tracking Test	Visual attention, executive function, working memory	Participants stood at a standardised distance from the screen with computer screen adjusted to eye height and feet placed at hip width apart. The aim was to follow randomly moving green circles among red distractors. In each trial, flashing green circles appeared alongside red distractors. When the participant was ready, the circles turned red and moved independently on the screen for 10s. When the circles stopped, random numbers appeared next to them. Participants were then required to identify the original flashing green target by stating the number or pointing to the circle. Trials increased in difficulty: Trials 1 – 4 had one target, Trials 5 – 8 had two targets, Trials 9 – 12 had three targets and Trials 13-15 had four targets.	Total number of correct trials. Test stopped after three errors made in three different trials or completion of 15th trial (whichever occurred first)	Computer
Van Biesen et al. [55]	Color Trails Test	Mental set-shifting	Color Trails 1:<br>Participants required to draw a line connecting encircled numbers 1 through 25, arranged randomly on the test sheet, in numerical order as quickly as possible.<br>Color Trials 2:<br>All numbers from 1 to 25 were encircled twice on the test sheet, once in a pink circle and once in a yellow circle. Participants were tasked with connecting the 25 numbers, alternating between the two colours.	Time (s) to complete	Color Trails Testing sheet
	Updating World Span Task	Working memory	Participants were orally presented with a list of items which they had to recall based on the criterion 'smallest object/s within each list' and in order of presentation. The working memory load increases from level 1 to level 5, with each level having two trials. The number of items in each trial's word list is twice (trial 1) and two and a half time (trial 2) the number of items to be recalled. Test was terminated when participants earned a score of zero on both trials of a level.	A point was given for each correctly recalled item in the correct sequence, with a minimum score of 0 and maximum score of 30.	NA

2E Methods of assessment for eligible studies regarding Vision Impairment

Study	Measure of Visual Function	Test	Method of Assessment	Outcome Measure
Allen et al. [56], Latham et al. [60], Myint et al. [61], Stalin et al. [62], Stalin et al. [63]	Visual Acuity	Early Treatment Diabetic Retinopathy Study	Participants read characters from a handheld ETDRS LogMAR chart at a distance of 4m. If the participant had difficultly reading the largest characters on the chart, the distance was halved to 2m, and reduced further to 1m if necessary [56, 60, 61]. A distance of 1m alone was used in the following studies [62, 63]	Letter by letter scoring (logMAR units)
Allen et al. [56], Fortin-Guichard et al. [57], Krabben et al. [58], Krabben et al. [59], Latham et al. [60], Myint et al. [61], Stalin et al. [62], Stalin et al. [63]	Visual Acuity	Berkeley Rudimentary Vision Test	The participant is shown three sets of cards from various distances. The participants needs to identify the direction (up, down, left, or right) the openings of the single tumbling E are facing or whether the grating stripes are positioned horizontally or vertically. For those unable to resolve the gratings, the test proceeds with the white field perception and black and white discrimination cards. If participants cannot discriminate black and white, a pen torch is used to check their perception of light.	logMar units
Allen et al. [56], Myint et al. [61]	Contrast Sensitivity	Pelli-Robson Chart	Pelli-Robson chart comprises eight rows, each displaying two triplets of letters. A test distance of 1m was used. Each triplet is of equal contrast and decrease by 0.15 logCS. Participants instructed to read each letter from left to right, and concluded when two letters from a triplet were incorrectly named.	Each correctly named letter scored 0.05 logCS
Allen et al. [56], Fortin-Guichard et al. [57], Krabben et al. [58], Latham et al. [60]	Contrast Sensitivity	Mars Chart	The Mars number chart consists of eight rows of six numbers, with each successive number lower in contrast by 0.04 logCS. Participants asked to read out the numbers with the test stopping when two consecutive numbers were incorrectly named.	Contrast level of the final number minus 0.04logCS for every incorrectly named number

Study	Measure of Visual Function	Test	Method of Assessment	Outcome Measure
Stalin et al. [62], Stalin et al. [63]	Contrast Sensitivity	Quick contrast sensitivity procedure on adaptive sensory technology platform	During a trial at a viewing distance of 1m, three letters were displayed on a screed, with the left and middle letters presented at four and two times the contrast of the right letter, respectively. Contrast sensitivity was measured after completing 25 trials.	Area under the log CSF curve (AULCSF, logCS units)
Fortin-Guichard et al. [57], Krabben et al. [58]	Light Sensitivity	Mars Chart using Brightness Acuity Tester (BAT)	Participants performed Mars test monocularly while looking through the BAT with the light source. The light source was then turned on and Mars test repeated.	Difference in logCS between lighting conditions [57] Percentage loss between first and second test [58]
Stalin et al. [62], Stalin et al. [63]	Light Sensitivity	Static visual acuity measures in presence of bright light	Assessed using static visual acuity measures (Early Treatment Diabetic Retinopathy Study/ Berkeley Rudimentary Vision Test) under increased light levels (approximately. 1900 lux).	Change in logMAR from static visual acuity measures with and without presence of light
Stalin et al. [62], Stalin et al. [63]	Glare Sensitivity & Glare Recovery	Static visual acuity measures in presence/removal of bright light	Glare sensitivity estimated by measuring the static visual acuity (Early Treatment Diabetic Retinopathy Study/ Berkeley Rudimentary Vision Test) of participants immediately after introducing a bright, binocular glare source in the line of sight. Glare recovery was measured by retesting the static visual acuity 1 minute after removing the glare source	Glare sensitivity calculated as the difference between static visual acuity in the presence of glare and static visual acuity at baseline. Glare recovery calculated as the difference between static visual acuity after the removal of glare source and static visual acuity at baseline.

Study	Measure of Visual Function	Test	Method of Assessment	Outcome Measure
Stalin et al. [62], Stalin et al. [63]	Dynamic Visual Acuity	Computer program with a single moving tumbling E letter	A single moving tumbling E presented on a high definition television screen at a distance of 1m. Initial size of the letter presented was 0.60 log units larger than the participants static visual acuity. Five targets were presented per 0.1logMAR step and display time was set to unlimited. The sequence continued until the participant could no longer identify three out of the five targets of the same size.	logMAR using a per letter scoring system
Fortin-Guichard et al. [57], Krabben et al. [58]	Depth Perception	Modified version of Howard-Dolman test	Participants were seated 1.5m from a stationary target (white rod – 20mm diameter). They had to move the other target positioned on another rail until it aligned with the stationery one. The test had two conditions: sliding the target from either end of the rail. The distance between the centre of the two targets was measured (mm). The background of the test was black, and a black barrier blocked the participants lower field of view to remove any visual cues from the base and rail of the targets.	Mean absolute value across all six trials [57]<br>Mean absolute error (mm) across all four trials [58]
Fortin-Guichard et al. [57], Krabben et al. [58]	Visual Search	Custom-made visual search test	Participants were required to identify whether a circle was present among squares in a grid. The easiest level had a 3x3 grid, the intermediate level had an 8x8 grid, and the most difficult level had a 15x15 grid of black shapes on a white background. The full test consisted of 18 trials for each level of difficulty, where a circle was present in 12 of the 18 trials. The order and location of the circle were randomised. Each trial lasted 30 seconds, and participants used the arrow keys on a keyboard to respond (upward for circle present, downward for circle absent). A missed response was marked as incorrect.	Response time for the most difficult level completed where circle was present [57]<br>Mean response times in each of the three difficulty levels where a circle was present were added together [58]

Study	Measure of Visual Function	Test	Method of Assessment	Outcome Measure
Fortin-Guichard et al. [57], Krabben et al. [58]	Motion Perception	Custom-made computer based test using a random dot kinematogram	Participants sat 66cm from a 27-inch Apple Thunderbolt display showing a square envelope with 100 briefly appearing moving dots. Following six familiarization trials, where all dots moved uniformly either upward or downward, participants proceeded to the full protocol upon correctly responding to at least four of these trials. A one-up-two-down staircase procedure with five reversals determined the threshold coherence level for correctly identifying global motion in 66.6% of presentations. Coherence began at 100%, decreasing by 25% before the first reversal. Using the upward and downward arrow keys, participants recorded their forced-choice responses on a keyboard. Each 8s trial required a response, and a missed response was considered incorrect.	Threshold coherence level (%) [57]<br><br>NR [58]
Stalin et al. [62], Stalin et al. [63]	Translational and radial motion perception	Random dot kinematograms	Random dot kinematograms consisting of 100 individual, full contrast, local dots equivalent to the size of the target detail of a 2.00 logMAR letter were used to assess translational (up and down motion) and radial (in and out) motion. The stimuli was displayed on high definition television screens, with each trial lasting 16s. Participants were required to identify the motion direction of the signal dots. The difficulty of the stimulus was adjusted based on the participants performance and testing concluded after eight reversals.	Threshold calculated by averaging last six reversals (%).

2F Methods of assessment for eligible studies regarding Activity Limitation Measures

Study	Test	Method of Assessment	Outcome Measure	Equipment
Altmann et al. [24]	10m sprint	Participants performed a 10m straight-line sprint from standstill.	Time (s)	Two sets of infrared sensors placed at start and 10m mark
	Turn test	Participants performed a 10m straight line sprint, performed a 180° turn as fast as possible and sprinted 10m back to the start line.	Time (s) to complete course	Two sets of infrared sensors placed at start and 10m mark
Altmann et al. [24] & Altmann et al. [32]	Tilt test	Participants seated in everyday wheelchair. Triangular bumpers were used to fix one of the wheels of the chair to the ground. Arms crossed in front of chest and were instructed to try and lift the non-fixed wheel off the ground using only legs and trunk.	Difference between max and initial height (mm).	Tape measure attached to pulley system
	Acceleration test	Participants accelerated from standstill and maintained maximum velocity for 3 – 5m.	Time (s) to cover 1m [32].<br>Time (s) to cover 1m, 2m, 3m, 4m [24].	Cheetah LMT (AMR sports, Queensland, Australia) attached to wheelchair measured displacement.
	Hitting	No specific test performed. Impulse with which athlete can hit opponent was calculated from results of acceleration test.	Maximum velocity after 2m multiplied by mass of body and wheelchair.	N/A
Daniel et al. [64]	Ball dribbling in a straight line	Within a defined area (3m x 22m), participants dribble the ball over 20m, and then subsequently run an additional 2m beyond the finish line. The test is deemed invalid if the ball crosses outside the specified area during the test.	Time (s) to complete	Stopwatch (Casio HS-3V, Tokyo, Japan)

Study	Test	Method of Assessment	Outcome Measure	Equipment
Daniel et al. [64]	Ball dribbling with Short Slalom CODs	Within a defined area (3m x 11m), participants dribble the ball between seven cones placed 1.5m apart and 1m from the start/finish lines. The test is deemed invalid if the ball crosses outside the specified area or if the participants knocks over a cone during the test.	Time (s) to complete	Stopwatch (Casio HS-3V, Tokyo, Japan)
	Ball dribbling with Long Slalom CODs	Within a defined area (8m x 26m), participants dribble the ball between five cones placed 6.4m apart (diagonal distance). The test is deemed invalid if the ball crosses outside the specified area or if the participants knocks over a cone during the test.		
	Ball dribbling in a square	Within a defined area (4m x 4m), the participant dribbles the ball from the bottom left cone and follows a sequence that includes: (1) running diagonally to the front right cone, (2) changes direction to the left and runs to front left cone, (3) changes direction to the right and runs diagonally to the back right cone and (4) changes direction to the left running towards the initial cone where the test commenced. The test is deemed invalid if participant loses control of the ball or knocks over one of the cones during the test.		
Henríquez et al. [65]	10m sprint	Participants instructed to perform 10m linear sprint (standing start) as fast as possible.	Time (s)	Infrared photocells (Witty system; Microgate)
Fortin-Guichard et al. [57]	Ability to swim in straight line (mean lateral position in lane)	Video footage was captured from an elevated vantage point at the end of the pool, allowing for manual digitisation of the swimmer's lateral position within the lane.	Average absolute distance from centre of the lane (cm)	Video camera (Go Pro 3)<br>Kinoeva software

Study	Test	Method of Assessment	Outcome Measure	Equipment
Reina et al. [73], Roldan et al. [74], Henríquez et al. [65]	505 agility test	Run 10m, sprint 5m, turn 180°, sprint 5m. Timing gates positioned at start and finish of the test. Performed without ball [65,73]. Performed with ball [74].	Time (s) to complete course	Timing gates (Globus^{TM}) [73]<br>Infrared photocells (Witty system; Microgate, Bolzano, Italy) [65,74]
Hogarth et al. [66]	Tethered swim	An inelastic cord linked load cell (fixed to pool end wall 0.5m above water level) to belt positioned around participants waist, so they were situated 5m from pool end. Participants performed single maximal effort for 30s using preferred freestyle swim style. Force-time data divided in 6x5s windows.	Maximum tether force in Newtons (Highest average tether force recorded within one 5s window.<br>Average tether force in Newtons (Mean force recorded over 30s).<br>Fatigue index (Decline in mean tether force over 30 s).	Submersible in-line load cell (DDEN-500N; Applied Measurements Ltd, Reading, UK)
Hogarth et al. [67]	Passive drag	Participants towed by electrical mechanical towing rig on surface of water at 1.5m.s^{-1}. Participants attached to inelastic steel cable by holding onto a small handle, belt secured around upper torso, or thin rubber tubing (negligible buoyancy) wrapped around the upper arms depending on impairment. Towing (cable) force was measured by load cell positioned 5 m in front of swimmer. Participants were instructed to hold their breath and be in their most streamlined position for the duration of the tow (25-30m). Four second period in which cable was most stable was used to calculate mean passive drag force.	Normalised passive drag (N.kg^{-1})	Electrical mechanical towing rig<br>Submersible in-line load cell

Study	Test	Method of Assessment	Outcome Measure	Equipment
Nogueira et al. [68], Reina et al. [73], Reina et al. [46], Sarabia et al. [75]	Split jumps	Starting with one foot in front of the other, participants jumped into the air simultaneously switching their feet to land with opposite foot in front (One cycle = left front foot contact to next left front foot contact).	Time (s) to complete 25 cycles	Equipment NR [68]<br><br>Contact mat (Tapeswitch CVP-2335) [46,73,75]
Peña-González et al. [69], Reina et al. [70], Reina et al. [73], Roldan et al. [74], Sarabia et al. [75]	Modified agility test	Participants begin behind start line, sprint forward 5m, side-step 2.5m to right, side-step 5m to left, side-step 2.5m to right and sprint backwards 5m. Timing gates positioned at start/finish line.	Time (s) to complete course	Photocell system (Witty System; Microgate, Bolzano Italy) [69,74]<br><br>Electronic timing system (Globus, Codogne, Italy) [70,73,75]
Peña-González et al. [69]	Dribbling speed test	Four cones set up in a 9m x 9m square with an additional cone placed between the start and finish line (4.5m). Participants required to dribble ball around all the cones. Timing gates positioned at start and finish line.	Time (s) to complete course	Photocell system (Witty System; Microgate, Bolzano Italy)
Reina et al. [72], Reina et al. [73], Roldan et al. [74], Sarabia et al. [75]	Standing broad jump	Standing behind a line, participants jumped forward as far as possible taking off and landing with both feet simultaneously.	Distance (m) from start line to heel strike. Normalised to standing height [72,73,75]	Tape measure

Study	Test	Method of Assessment	Outcome Measure	Equipment
Reina et al. [70], Reina et al. [71], Yanci et al. [78]	Illinois agility test	Participants sprint 10m, turn 180° around cone and return to the starting line. Swerve in and out of four cones, sprints 10m, turns 180° around cone and sprints 10m to finish line. Timing gates positioned at start and finish line. Performed without ball [70,71]. Performed with ball [71, 78].	Time (s) to complete course	Electronic timing system (Globus, Codogne, Italy)
Reina et al. [71], Yanci et al. [78], Sarabia et al. [75]	Stop and go test	Participants sprint 10m to first contact mat, stop completely for 2s. A beep sounds and participant sprints 10m to second contact mat where they stop completely for another 2s. A beep sounds and participant sprints 10m to finish line. Performed with and without ball [71]. Performed with ball [78]. Performed without ball [75].	Time (s) to complete course [71]<br>Time (s) taken to cover first 10m, second 10m, final 10m and total time to complete course [75,78].	Electronic timing system (Globus, Codogne, Italy)<br>Two contact mats (59 x 88cm) (CVP-2335; Tapeswitch, Farmingdale, NY)
Reina et al. [71], Sarabia et al. [75]	40m sprint	Participants instructed to perform a 40m linear sprint (standing start) as fast as possible. Timing gates positioned at start, 10m, 25m and 40m. Performed with and without dribbling ball.	Time (s) recorded at 10m, 25m and 40m	Infrared photocells (Globus, Codogne, Italy)
Reina et al. [72], Reina et al. [73], Roldan et al. [74], Sarabia et al. [75]	Vertical jump (CMJ)	Participants jumped as high as possible, bending their knees to approx. 90◦ during take-off and hands on hips or near their torso (if unable to place on hips).	Jump height (m) [72,74]<br>Jump height (cm) [73, 75]	Force platform (9287B; Kistler, Winterthur, Switzerland) [72]<br>Leg stiffness device (Opto Jump Next™, Microgate) [73,75]<br>Equipment NR [74]

Study	Test	Method of Assessment	Outcome Measure	Equipment
Reina et al. [72], Reina et al. [73], Roldan et al. [74], Sarabia et al. [75]	Triple hop for distance	Participants performed three consecutive hops, landing on same leg. Swinging of arms permitted. Both limbs assessed.	Distance (m) from start line to landing heel. Normalised to standing height [72,73,75].	Tape measure
Reina et al. [72], Reina et al. [73], Sarabia et al. [75]	Four bounds for distance	Starting in a standing position, participants performed four forward jumps alternating between left and right legs.	Distance (m) jumped from start to heel strike of fourth bound. Normalised to standing height.	Tape measure
Reina et al. [73], Sarabia et al. [75]	One leg stance	Participants barefoot, focusing on spot on wall at eye level, arms across chest. Time starts as soon as participant lifts foot off the floor and stops if any of the following occur: arms uncrossed, raised foot touches floor, move weight bearing foot or exceeded 20s. Both legs assessed.	Time (s) to keep balance up to maximum 20s.	Stopwatch (Casio HS-80TW-1EF)
Reina et al. [73], Roldan et al. [74], Sarabia et al. [75]	Tandem walk	Participant walked barefoot heel to toe along 5m line as fast as possible with arms across chest.	Time (s) to complete 10 steps [75].<br>Time (s) to complete 5m [73-75].	Stopwatch (Casio HS-80TW-1EF)
Nogueira et al. [68], Reina et al. [73], Sarabia et al. [75]	Side stepping	Participants jumped over two lines (placed 40cm apart) symmetrically performing leg open and close movements. (One cycle = adduction-abduction cycles of legs).	Time (s) to complete 15 cycles.	Equipment NR [68]<br>Contact mat (Tapeswitch CVP-2335) [73,75]

Study	Test	Method of Assessment	Outcome Measure	Equipment
Reina et al. [73], Roldan et al. [74]	20m sprint	Participants instructed to perform a 20m linear sprint (standing start) as fast as possible.	Time (s) at 5m, 10m, 15m and 20m [73].<br>Time (s) at 5m, 10m and 20m [74].	Timing gates (Globus^{TM}) [73]<br>Infrared photocells (Witty system; Microgate, Bolzano, Italy) [74]
Roldan et al. [74]	Rapid heel-toe contacts	Participants seated. Alternate between heel and toe contacts for a total of 25 cycles (one cycle = one heel and one toe contact). Required to perform the test as fast as possible. Only contacts within the area were counted. Both feet assessed.	Time (s) to complete 25 correct cycles.	Tapping platform (35 x 20cm)
Sarabia et al. [75]	Rapid-heel toe placement	Participant seated, barefoot. Touches the corners of a 20 x 30cm rectangle on the floor, alternating heel, and toe in each corner in a clockwise then anticlockwise direction.	Time (s) to complete and number of incorrect contacts on corners recorded.	Stopwatch (Casio HS-80TW-1EF).<br>Contact mat (Tapeswitch CVP-2335)
Sarabia et al. [75]	Side step	Performed barefoot. A starting line and a 10m line perpendicular are marked on the floor. Participants start in a standing position with feet together. Participants perform five repetitions of side steps, aiming to step as wide as possible without using their arms or jumping.	Distance (m)	Tape measure
Sarabia et al. [75]	Running in place	Participant ran on the same spot as fast as possible for 25 cycles. A cycle is right foot contact to next right foot contact.	Time (s) to complete 25 correct cycles.	Contact mat (Tapeswitch CVP-2335)
Sarabia et al. [75]	Hexagon agility test	Hexagon with 60cm sides and 120° angles marked on hard-surface floor. Participants begin in the middle of the hexagon and perform double leg jumps over each side and back to the centre in a clockwise direction for three complete rounds around the hexagon (18 jumps).	Time (s) to complete 3 revolutions around the hexagon.	Stopwatch (Casio HS-80TW-1EF).<br>Marker tape
Sarabia et al. [75]	10m speed skip	Markers were positioned at 0, 10m and 20m, with timing gates positioned at 10m and 20m. Participants performed a skip-hop-step-hop pattern. Participants accelerated over the initial 10m and maintained speed through to second gate (20m).	Time (s) to move from 10m to 20m.	Timing gates (Globus)

Study	Test	Method of Assessment	Outcome Measure	Equipment
Van Biesen et al. [76]	Semi-standardised Tactical Proficiency Table Tennis Test	Players faced an able-bodied opponent in 12 series of five rallies. There were six service types delivered in a standardised sequence: no spin to backhand, backspin to forehand, topspin to middle, left sidespin to backhand, backspin-sidespin combination to forehand and topspin-sidespin combination to middle. Each of these service types could be short or long, so there were 12 service types in total. Each player received all 12 service types five times consecutively, resulting in 60 services per player. An expert rated their performance on a scale from 0-8 points for each return and subsequent rally.	Points per rally<br>(Score out of 40)	Video camera
Van Biesen et al. [77]	400m Pacing Test	Eleven cones are positioned around a 400m running track at marked distances (20m, 40m, 60m, 80m, 120m, 160m, 200m, 250m, 300m, 350m and 400m). Participants are required to complete a 400m run at a constant submaximal pace set at 80% of their personal best time for a 1500m distance. During the first 200m, participants receive guidance through auditory signals and coach feedback. For the remaining 200m, participants are required to maintain the same pace without any feedback.	Split times (s)	Cones<br>Whistle
Yanci et al. [78]	Turning and dribbling test	Sprint 10m, change direction 180° (run around cone), sprint 10m, change direction 180°, sprint 10m to finish line. Performed while dribbling ball.	Time (s) to complete first 10m and total time to complete course	Two photocells (Globus)

2G Reference List

Please note that the reference numbers used in the tables correspond to those presented in the manuscript. However, for ease of reference, a standalone reference list has been created.

18. Hogarth L, Nicholson V, Spathis J, Tweedy S, Beckman E, Connick M, et al. A battery of strength tests for evidence-based classification in Para swimming. J Sports Sci. 2019;37(4):404-13. https://doi.org/10.1080/02640414.2018.1504606

19. Hogarth L, Payton C, Nicholson V, Spathis J, Tweedy S, Connick M, et al. Classifying motor coordination impairment in Para swimmers with brain injury. J Sci Med Sport. 2019;22(5):526-31. https://doi.org/10.1016/j.jsams.2018.11.015

24. Altmann VC, Groen BE, Hart AL, Vanlandewijck YC, Limbeek J, Keijsers NLW. The impact of trunk impairment on performance-determining activities in wheelchair rugby. Scand J Med Sci Sports. 2017;27(9):1005-14. https://doi.org/10.1111/sms.12720

31. Altmann VC, Groen BE, Groenen KH, Vanlandewijck YC, van Limbeek J, Keijsers NL. Construct validity of the trunk impairment classification system in relation to objective measures of trunk impairment. Arch Phys Med. 2016;97(3):437-44. https://doi.org/10.1016/j.apmr.2015.10.096

32. Altmann VC, Groen BE, Hart AL, Vanlandewijck YC, Keijsers NLW. Classifying trunk strength impairment according to the activity limitation caused in wheelchair rugby performance. Scand J Med Sci Sports. 2018;28(2):649-57. https://doi.org/10.1111/sms.12921

33. Beckman EM, Connick MJ, Tweedy SM. How much does lower body strength impact Paralympic running performance? Eur J Sport Sci. 2016;16(6):669-76. https://doi.org/10.1080/17461391.2015.1132775

34. Connick MJ, Beckman E, Vanlandewijck Y, Malone LA, Blomqvist S, Tweedy SM. Cluster analysis of novel isometric strength measures produces a valid and evidence-based classification structure for wheelchair track racing. Br J Sports Med. 2018;52(17):1123-9. https://doi.org/10.1136/bjsports-2017-097558

35. Domínguez-Díez M, Raya-González J, Elvira JLL, Reina R. Multi-joint isometric measurement for the evidence-based assessment of upper limb strength impairment in wheelchair athletes with different health conditions: A preliminary study. Biol Sport. 2022;40(3):723-730. https://doi.org/10.5114/biolsport.2023.119286

36. Hyde A, Hogarth L, Sayers M, Beckman E, Connick MJ, Tweedy S, et al. The impact of an assistive pole, seat configuration, and strength in paralympic seated throwing. Int J Sports Physiol Perform. 2017;12(7):977-83. https://doi.org/10.1123/ijspp.2016-0340

37. Liljedahl JB, Arndt A, Nooijen CF, Bjerkefors A. Isometric, dynamic, and manual muscle strength measures and their association with cycling performance in elite paracyclists. Am J Phys Med Rehabil. 2023;102(5):461-467. https://doi.org/10.1097/PHM.0000000000002014

38. Liu K, Ji L, Ma H, Lu Y. Cluster analysis of multiple impairment measures in evidence-based classification for para-alpine sit skiers. Scand J Med Sci Sports. 2024;34:e14514. https://doi.org/10.1111/sms.14514

39. Mason BS, Altmann VC, Hutchinson MJ, Goosey-Tolfrey VL. Validity and reliability of isometric tests for the evidence-based assessment of arm strength impairment in wheelchair rugby classification. J Sci Med Sport. 2020;23(6):559-63. https://doi.org/10.1016/j.jsams.2019.12.022

40. Mason BS, Altmann VC, Hutchinson MJ, Petrone N, Bettella F, Goosey-Tolfrey VL. Optimising classification of proximal arm strength impairment in wheelchair rugby: A proof of concept study. J Sports Sci. 2021;39(sup1):132-9. https://doi.org/10.1080/02640414.2021.1883291

41. Rosso V, Gastaldi L, Rapp W, Lindinger S, Vanlandewijck Y, Äyrämö S, et al. Balance perturbations as a measurement tool for trunk impairment in cross-country sit skiing. Adapt Phys Activ Q. 2019;36(1):1-76. https://doi.org/10.1123/apaq.2017-0161

42. Vanlandewijck YC, Verellen J, Beckman E, Connick M, Tweedy SM. Trunk strength effect on track wheelchair start: implications for classification. Med Sci Sports Exerc. 2011;43(12):2344-51. https://doi.org/10.1249/MSS.0b013e318223af14

43. Altmann VC, Groen BE, Groeneweg S, van der Weijde G, Keijsers NL. Validation of new measures of arm coordination impairment in wheelchair rugby. J Sports Sci. 2021;39(sup1):91-8. https://doi.org/10.1080/02640414.2021.1882731

44. Connick M, Beckman E, Spathis J, Deuble R, Tweedy SM. How much do range of movement and coordination affect Paralympic sprint performance? Med Sci Sports Exerc. 2015;47(10):2216-23. https://doi.org/10.1249/MSS.0000000000000643

45. Maia AC, Hogarth L, Burkett B, Payton C. Improving the objectivity of the current world para swimming motor coordination test for swimmers with hypertonia, ataxia and athetosis using measures of movement smoothness, rhythm and accuracy. J Sports Sci. 2021;39(sup1):62-72. https://doi.org/10.1080/02640414.2021.1935114

46. Reina R, Iturricastillo A, Castillo D, Roldan A, Toledo C, Yanci J. Is impaired coordination related to match physical load in footballers with cerebral palsy of different sport classes? J Sports Sci. 2021;39(sup1):140-9. https://doi.org/10.1080/02640414.2021.1880740

47. Roldan A, Sabido R, Barbado D, Caballero C, Reina R. Manual dexterity and intralimb coordination assessment to distinguish different levels of impairment in boccia players with cerebral palsy. Front Neurol. 2017;8:582. https://doi.org/10.3389/fneur.2017.00582

48. Bjerkefors A, Rosén JS, Tarassova O, Arndt A. Three-dimensional kinematics and power output in elite para-kayakers and elite able-bodied flat-water kayakers. J Appl Biomech. 2019;35(2):93-100. https://doi.org/10.1123/jab.2017-0102

49. Nicholson VP, Spathis JG, Hogarth LW, Connick MJ, Beckman EM, Tweedy SM, et al. Establishing the reliability of a novel battery of range of motion tests to enable evidence-based classification in Para Swimming. Phys Ther Sport. 2018;32:34-41. https://doi.org/10.1016/j.ptsp.2018.04.021

50. Pineda RC, Krampe RT, Vanlandewijck Y, Van Biesen D. Cognitive-motor multitasking in athletes with and without intellectual impairment. Scand J Med Sci Sports. 2022;32(2):424-434. https://doi.org/10.1111/sms.14088

51. Van Biesen D, McCulloch K, Janssens L, Vanlandewijck YC. The relation between intelligence and reaction time in tasks with increasing cognitive load among athletes with intellectual impairment. Intelligence. 2017;64:45-51. https://doi.org/10.1016/j.intell.2017.06.005

52. Van Biesen D, Mactavish J. Cognitive predictors of performance in well-trained table tennis players with intellectual disability. Adapt Phys Activ Q. 2016;33(4):324-337. https://doi.org/10.1123/APAQ.2015-0122

53. Van Biesen D, Mactavish J, McCulloch K, Lenaerts L, Vanlandewijck YC. Cognitive profile of well-trained athletes with intellectual disabilities. Res Dev Disabil. 2016;53:377-390. https://doi.org/10.1016/j.ridd.2016.03.004

54. Van Biesen D, Jacobs L, McCulloch K, Janssens L, Vanlandewijck YC. Cognitive-motor dual-task ability of athletes with and without intellectual impairment. J Sports Sci. 2018;36(5):513-521. https://doi.org/10.1080/02640414.2017.1322215

55. Van Biesen D, Pineda RC, Van Damme T, Burns J. The impact of intellectual disability and sport expertise on cognitive and executive functions. J Intellect Disabil. 2023;27(1):104-120. https://doi.org/10.1177/17446295211036331

56. Allen PM, Latham K, Ravensbergen RHJC, Myint J, Mann DL. Rifle shooting for athletes with vision impairment: Does one class fit all? Front Psychol. 2019;10:1727. https://doi.org/10.3389/fpsyg.2019.01727

57. Fortin-Guichard, Ravensbergen HJC, Krabben K, Allen PM, Mann DL. The relationship between visual function and performance in para swimming. Sports Med Open. 2022;8(1):1-18. https://doi.org/10.1186/s40798-022-00412-3

58. Krabben K, Ravensbergen RHJC, Orth D, Fortin-Guichard D, Savelsbergh GJP, Mann DL. Assessment of visual function and performance in paralympic judo for athletes with vision impairment. Optom Vis Sci. 2021;98(7):854-863. https://doi.org/10.1097/OPX.0000000000001735

59. Krabben K, Mashkovskiy E, Ravensbergen HJC, Mann DL. May the best sighted win? The relationship between visual function and performance in para judo. J Sports Sci. 2021;39(sup1):188-197. https://doi.org/10.1080/02640414.2020.1851899

60. Latham K, Mann DL, Dolan R, Myint J, Timmis MA, Ryu D, et al. Do visual fields need to be considered in classification criteria within visually impaired shooting? J Sports Sci. 2021;39(sup 1):150-158. https://doi.org/10.1080/02640414.2021.1911425

61. Myint J, Latham K, Mann D, Gomersall P, Wilkins AJ, Allen PM. The relationship between visual function and performance in rifle shooting for athletes with vision impairment. BMJ Open Sport Exerc Med. 2016;2(1):e000080. https://doi.org/10.1136/bmjsem-2015-000080

62. Stalin A, Dalton K. Classification in para skiing: do better performing skiers have better visual functions? Front Sports Act Living. 2023;5:1046318. https://doi.org/10.3389/fspor.2023.1046318

63. Stalin A, Creese M, Dalton KN. Do impairments in visual functions affect skiing performance? Front Neurosci. 2021;15:648648. https://doi.org/10.3389/fnins.2021.648648

64. Daniel LF, Reina R, Gorla, JI, Bastos T, Roldan A. Validity and reliability of a test battery to assess change of directions with ball dribbling in para-footballers with cerebral palsy. Brain Sci. 2020;10(2):74. https://doi.org/10.3390/brainsci10020074

65. Henríquez M, Peña-González I, Albaladejo-García C, Sadrangani KP, Reina R. Sex differences in change of direction deficit and asymmetries in footballs with cerebral palsy. Scand J Med Sci Sports. 2023;33(8):1519-1530. https://doi.org/10.1111/sms.14383

66. Hogarth L, Burkett B, Van de Vliet P, Payton C. Maximal fully tethered swim performance in para swimmers with physical impairment. Int J Sports Physiol Perform. 2020;15(6):816-24. https://doi.org/10.1123/ijspp.2019-0515

67. Hogarth L, Oh YT, Osborough C, Osborough C, Formosa D, Hunter A, et al. Passive drag in para swimmers with physical impairments: Implications for evidence-based classification in Para swimming. Scand J Med Sci Sports. 2021;31(10):1932-40. https://doi.org/10.1111/sms.14014

68. Nogueira CD, de Sá KSG, de Faria FR, Borges M, de Athayde Costa A, Gorla JI. Validation of split jump and the side-stepping jump coordination tests for cerebral palsy football athletes. Retos: nuevas tendencias en educación física, deporte y recreación. 2022(46):425-30.

69. Peña-González I, Roldan A, Toledo C, Urbán T, Reina R. Change-of-direction ability of para-footballers with cerebral palsy under a new evidence-based and sport-specific classification system. Int J Sports Physiol Perform. 2021;16(2):267-72. https://doi.org/10.1123/IJSPP.2019-0656

70. Reina R, Sarabia JM, Yanci J, García-Vaquero MP, Campayo-Piernas M. Change of direction ability performance in cerebral palsy football players according to functional profiles. Front Physiol. 2016;6:409. https://doi.org/10.3389/fphys.2015.00409

71. Reina R, Sarabia JM, Caballero C, Yanci J. How does the ball influence the performance of change of direction and sprint tests in para-footballers with brain impairments? implications for evidence-based classification in CP-Football. PloS one. 2017;12(11):e0187237. https://doi.org/10.1371/journal.pone.0187237

72. Reina R, Iturricastillo A, Sabido R, Campayo-Piernas M, Yanci J. Vertical and horizontal jump capacity in international cerebral palsy football players. Int J Sports Physiol Perform. 2018;13(5):1-603. https://doi.org/10.1123/ijspp.2017-0321

73. Reina R, Iturricastillo A, Castillo D, Urbán T, Yanci J. Activity limitation and match load in para-footballers with cerebral palsy: an approach for evidence-based classification. Scand J Med Sci Sports. 2019;30(3):496-504. https://doi.org/10.1111/sms.13583

74. Roldan A, Henríquez M, Iturricastillo A, Castillo D, Yanci J, Reina R. To what degree does limb spasticity affect motor performance in para-footballers with cerebral palsy? Front Physiol. 2022:2510. https://doi.org/10.3389/fphys.2021.807853

75. Sarabia JM, Roldan A, Henríquez M, Reina R. Using decision trees to support classifiers' decision-making about activity limitation of cerebral palsy footballers. Int J Environ Res Public Health. 2021;18(8):4320. https://doi.org/10.3390/ijerph18084320

76. Van Biesen D, Mactavish J, Vanlandewijck Y. Tactical proficiency among table tennis players with and without intellectual disabilities. Eur J Sport Sci. 2;14(5):103-409. https://doi.org/10.1080/17461391.2013.825645

77. Van Biesen D, Hettinga FJ, Mcculloch K, Vanlandewijck YC. Pacing ability in elite runners with intellectual impairment. Med Sci Sports Exerc. 2017;49(3):588-594. https://doi.org/10.1249/MSS.0000000000001115

78. Yanci J, Castillo D, Iturricastillo A, Aracama A, Roldan A, Reina R. Performance analysis in football-specific tests by para-footballers with cerebral palsy: Implications for evidence-based classification. Int J Sports Physiol Perform. 2021;16(9):1328-34. https://doi.org/10.1123/ijspp.2020-0370
